# Cav2.3 channels contribute to dopaminergic neuron loss in a model of Parkinson’s disease

**DOI:** 10.1038/s41467-019-12834-x

**Published:** 2019-11-08

**Authors:** Julia Benkert, Simon Hess, Shoumik Roy, Dayne Beccano-Kelly, Nicole Wiederspohn, Johanna Duda, Carsten Simons, Komal Patil, Aisylu Gaifullina, Nadja Mannal, Elena Dragicevic, Desirée Spaich, Sonja Müller, Julia Nemeth, Helene Hollmann, Nora Deuter, Yassine Mousba, Christian Kubisch, Christina Poetschke, Joerg Striessnig, Olaf Pongs, Toni Schneider, Richard Wade-Martins, Sandip Patel, Rosanna Parlato, Tobias Frank, Peter Kloppenburg, Birgit Liss

**Affiliations:** 1 Institute of Applied Physiology, University of Ulm, Ulm, Germany; 2 Institute for Zoology, Biocenter, CECAD, University of Cologne, Cologne, Germany; 3 Oxford Parkinson’s Disease Centre, Department of Physiology, Anatomy and Genetics, University of Oxford, Oxford, United Kingdom; 4 Institute of Human Genetics, University Medical Center Hamburg-Eppendorf, Hamburg, Germany; 5 Department of Pharmacology and Toxicology, Institute of Pharmacy, Center for Molecular Biosciences, University of Innsbruck, Innsbruck, Austria; 6 Institute of Physiology, CIPMM, University of the Saarland, Homburg, Germany; 7 Institute for Neurophysiology, University of Cologne, Cologne, Germany; 8 Department of Cell and Developmental Biology, UCL, London, UK; 9 Department of Neurology, University Medicine Göttingen, Göttingen, Germany; 10 New College, University of Oxford, Oxford, UK

**Keywords:** Cell death in the nervous system, Ion channels in the nervous system

## Abstract

Degeneration of dopaminergic neurons in the substantia nigra causes the motor symptoms of Parkinson’s disease. The mechanisms underlying this age-dependent and region-selective neurodegeneration remain unclear. Here we identify Cav2.3 channels as regulators of nigral neuronal viability. Cav2.3 transcripts were more abundant than other voltage-gated Ca^2+^ channels in mouse nigral neurons and upregulated during aging. Plasmalemmal Cav2.3 protein was higher than in dopaminergic neurons of the ventral tegmental area, which do not degenerate in Parkinson’s disease. Cav2.3 knockout reduced activity-associated nigral somatic Ca^2+^ signals and Ca^2+^-dependent after-hyperpolarizations, and afforded full protection from degeneration in vivo in a neurotoxin Parkinson’s mouse model. Cav2.3 deficiency upregulated transcripts for NCS-1, a Ca^2+^-binding protein implicated in neuroprotection. Conversely, NCS-1 knockout exacerbated nigral neurodegeneration and downregulated Cav2.3. Moreover, NCS-1 levels were reduced in a human iPSC-model of familial Parkinson’s. Thus, Cav2.3 and NCS-1 may constitute potential therapeutic targets for combatting Ca^2+^-dependent neurodegeneration in Parkinson’s disease.

## Abstract (web summary)

Voltage-gated Ca^2+^ channels are thought to contribute to neurodegeneration of dopaminergic neurons. Here the authors find that the R-type channel Cav2.3 in substantia nigra dopaminergic neurons may contribute to neurodegeneration in a model of Parkinson’s disease, in contrast to the neuroprotective action of the neuronal Ca^2+^ sensor NCS-1.

## Introduction

Parkinson’s disease is a complex movement disorder that affects millions of people worldwide^1,2^. Its primary motor symptoms are caused by the progressive degeneration of dopaminergic midbrain neurons, particularly those within the substantia nigra (SN)^1,3^. Aging is a major risk factor for the disease, and although most cases are sporadic, a not insignificant proportion show monogenic inheritance patterns^4,5^. The pathogenic mechanisms underlying Parkinson’s disease remain unresolved and curative therapies are currently not available^6,7^.

Dopaminergic midbrain neurons display autonomous pacemaker activity, which is crucial for somatodendritic and axonal striatal dopamine release and voluntary movement control^8–11^. In SN dopaminergic neurons, this activity generates oscillatory increases in free cytosolic Ca^2+^ levels, which are thought to impart mitochondrial stress and render these neurons more vulnerable to degeneration by Parkinson’s disease stressors^12–14^. This stressful Ca^2+^-driven mode of action distinguishes dopaminergic neurons in the SN (and other vulnerable neurons^3,15^) from neighboring pacemaking dopaminergic neurons in the ventral tegmental area (VTA), which are spared in Parkinson’s disease^1,3,16^. The metabolically demanding Ca^2+^ oscillations, particularly those in distal dendrites of SN dopaminergic neurons, are sensitive to inhibitors of L-type voltage-gated Ca^2+^ channels, such as isradipine^13,14^.

Consistent with a role for activity-related Ca^2+^ signals in triggering neuronal demise in Parkinson’s disease is epidemiological evidence, correlating use of blood–brain barrier permeable L-type voltage-gated Ca^2+^ channel blockers with a reduced risk for developing the disease later in life^17,18^. These blockers are well-established drugs for treating high blood pressure^17^. But a recent phase-III clinical trial with isradipine to test its potential for neuroprotection in Parkinson’s disease patients (ClinicalTrials.gov NCT02168842)^19^ was negative^20^. Indeed, in Parkinson’s disease animal models, there is not full agreement regarding the extent of neuroprotection by L-type voltage-gated Ca^2+^ channel inhibition^21^. Notably, recent studies have identified T-type voltage-gated Ca^2+^ channels to be important for stressful activity-related Ca^2+^ oscillations particularly in proximal dendrites of SN dopaminergic neurons, and also for their vulnerability to degenerative stressors^14,22–25^. Whether other voltage-gated Ca^2+^ channels contribute to degeneration of SN dopaminergic neurons in Parkinson’s disease is unclear^26,27^.

L-type voltage-gated Ca^2+^ channels in the brain and in SN dopaminergic neurons in particular are built from Cav1.2 and Cav1.3 pore-forming α_1_-subunits^28^. These channels are emerging as key regulators of dopaminergic excitability. On the one hand, they stabilize pacemaker robustness thus sustaining metabolic demand^13,22,29,30^. But on the other, they can also inhibit spontaneous activity in an indirect feedback loop^22,30^. This feedback mechanism involves Cav1.3, which serves as a Ca^2+^ source for the neuronal Ca^2+^ sensor, NCS-1. NCS-1 is a Ca^2+^-binding protein that has been linked to a variety of neuronal functions in health and disease^31–33^. In SN dopaminergic neurons, NCS-1 binds in a Ca^2+^-dependent fashion to inhibitory dopamine D2-autoreceptors^30,34,35^. This interaction prevents D2-autoreceptor desensitization thereby promoting dopamine autoinhibition^30,32^. NCS-1 is therefore suggested to protect SN dopaminergic neurons from activity-related Ca^2+^ overload, and degeneration^31,36^ but direct supporting evidence is currently lacking.

Here, we combine single-cell molecular techniques, brain slice patch-clamp recordings and Ca^2+^ imaging with pharmacological and genetic tools to analyze the role of voltage-gated Ca^2+^ channels and NCS-1 for dopaminergic neuronal viability in Parkinson’s disease. We identify R-type voltage-gated Ca^2+^ channels (Cav2.3) as crucial contributors to somatic activity-related Ca^2+^ signaling in mature SN dopaminergic neurons, and to their selective degeneration in an in vivo model of Parkinson’s disease. Moreover, we identify a neuroprotective role for NCS-1.

## Results

### Cav2.3 is the most abundant Cav subtype in SN dopaminergic neurons

**Fig. 1:**
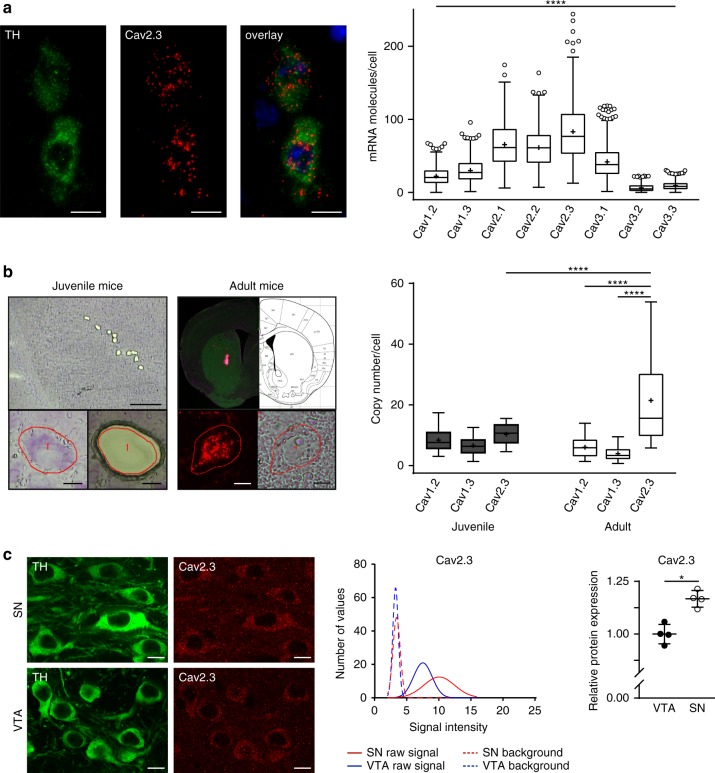
Cav2.3 is abundantly expressed in mature SN dopaminergic neurons. **a** Left: Representative images showing Cav2.3 (red) and tyrosine hydroxylase (TH; green) RNAScope fluorescence signals (combined with nuclear DAPI staining, blue) of individual SN dopaminergic neurons from an adult wildtype mouse. Scale bar: 10 µm. Right: Absolute mRNA transcript numbers per cell in adult SN dopaminergic neurons for distinct voltage-gated Ca^2+^ channel α-subunits, as indicated (Cav1.2: *n* = 667, Cav1.3: *n* = 537, Cav2.1: *n* = 425, Cav2.2: *n* = 461, Cav2.3: *n* = 435, Cav3.1: *n* = 657, Cav3.2: *n* = 573, Cav3.3: *n* = 498). **b** Left: Juvenile and adult mouse brain sections before and after UV-laser microdissection, in brightfield and fluorescent mode, respectively, and striatal injection site documentation of an in vivo retrogradely traced adult mouse, aligned with the mouse brain atlas. Scale bars: 250 µm and 10 µm, respectively. Right: Cell-specific UV-laser microdissection and reverse transcription quantitative PCR-based transcript molecule quantification in single mouse SN dopaminergic neurons upon aging for Cav1.2^#^ (juvenile: *n* = 26; adult: *n* = 25), Cav1.3^#^ (juvenile: *n* = 24; adult: *n* = 24) (^#^data partly modified from ref. ^37^), and Cav2.3 (juvenile: *n* = 13; adult: *n* = 27). **c** Left: Confocal images showing Cav2.3 antibody staining (red) of TH-positive (green) neurons in SN and VTA of an adult wildtype mouse, respectively. Scale bar: 10 µm. Middle: Histogram showing the immunosignal intensity distributions of plasma membrane Cav2.3 signal, and respective background signal intensities for all analyzed TH-positive SN (red) and VTA (blue) neurons, exemplary of one C57BL/6J mouse (SN: *n* = 145; VTA: *n* = 128). Right: Mean Cav2.3 immunosignal quantification in SN and VTA dopaminergic neurons for all analyzed mice (*n* = 4). Antibody specificity was confirmed on Cav2.3 knockout mice. Tukey's box plots are shown. Significances are indicated by asterisks: *p < 0.05, **p < 0.01, ***p < 0.001, ****p < 0.0001. All data are detailed in Supplementary Tables 1, 4A, 5A/B and Supplementary Fig. 6. Source data are available as a Source Data file

To probe the role of voltage-gated Ca^2+^ channels in the vulnerability of SN dopaminergic neurons, we performed quantitative RNAScope analysis to compare numbers of mRNA molecules encoding Cav α_1_-subunits in adult mice (Fig. 1a and Supplementary Table 1). mRNA for L-type (Cav1.2 and Cav1.3), P/Q-type (Cav2.1), N-type (Cav2.2), R-type (Cav2.3) and T-type (Cav3.1-3.3) voltage-gated Ca^2+^ channels were readily detected. Unexpectedly, mRNA for Cav2.3 R-type voltage-gated Ca^2+^ channels, which have yet to be studied in the context of neurodegeneration, were significantly higher than those for the other isoforms in adult SN dopaminergic neurons (Cav2.3 > Cav2.1 ≥ Cav2.2 > Cav3.1 > Cav1.3 > Cav1.2 > Cav3.3 > Cav3.2).

We also compared expression of α_1_-subunits in an independent approach by reverse transcription quantitative polymerase chain reaction (RT-qPCR) of single microdissected SN dopaminergic neurons. In these experiments, we used both juvenile and adult neurons. As shown in Fig. 1b (and Supplementary Table 4A), the expression of Cav1.2, Cav1.3 and Cav2.3 was similar in SN dopaminergic neurons from juvenile mice (PN13). In adult mice, transcripts for Cav1.3 and Cav1.2 were modestly downregulated as previously described^37^. In contrast, Cav2.3 expression was markedly upregulated during aging, such that it was the most abundant in adult neurons, consistent with the RNAScope analyses.

To extend Cav2.3 expression analysis to the protein level, we performed immunocytochemical analysis of Cav2.3 in adult mice (Fig. 1c and Supplementary Table 5A). We confirmed the specificity of the Cav2.3 antibody using Cav2.3 knockout mice in western blots and midbrain sections (Supplementary Fig. 6b/d and Supplementary Table 5B). Cav2.3 protein-derived immunofluorescence signals in the plasma membranes of SN dopaminergic neurons were readily detected. Notably, signal intensities were significantly higher in vulnerable SN dopaminergic neurons compared to that in VTA dopaminergic neurons, which are much less affected in Parkinson’s disease. Together these data prompted us to investigate the role of R-type voltage-gated Ca^2+^ channels, built by Cav2.3 pore-forming α_1_-subunits, in SN dopaminergic neuron function and viability.

### Cav2.3 contributes to activity-related Ca^2+^ oscillations

Previous work has linked Ca^2+^ entry upon autonomous pacemaking to Ca^2+^-dependent stress and degeneration of SN dopaminergic neurons, and showed that activity-related Ca^2+^ transients in dendritic compartments of SN dopaminergic neurons are inhibited by L-type and also T-type voltage-gated Ca^2+^ channel blockers^13,14^. We combined ratiometric Ca^2+^ imaging with perforated patch-clamp electrophysiology in brain slices to examine the role of Cav2.3 in activity-related Ca^2+^ dynamics in somata of dopaminergic midbrain neurons. We took two approaches.

**Fig. 2:**
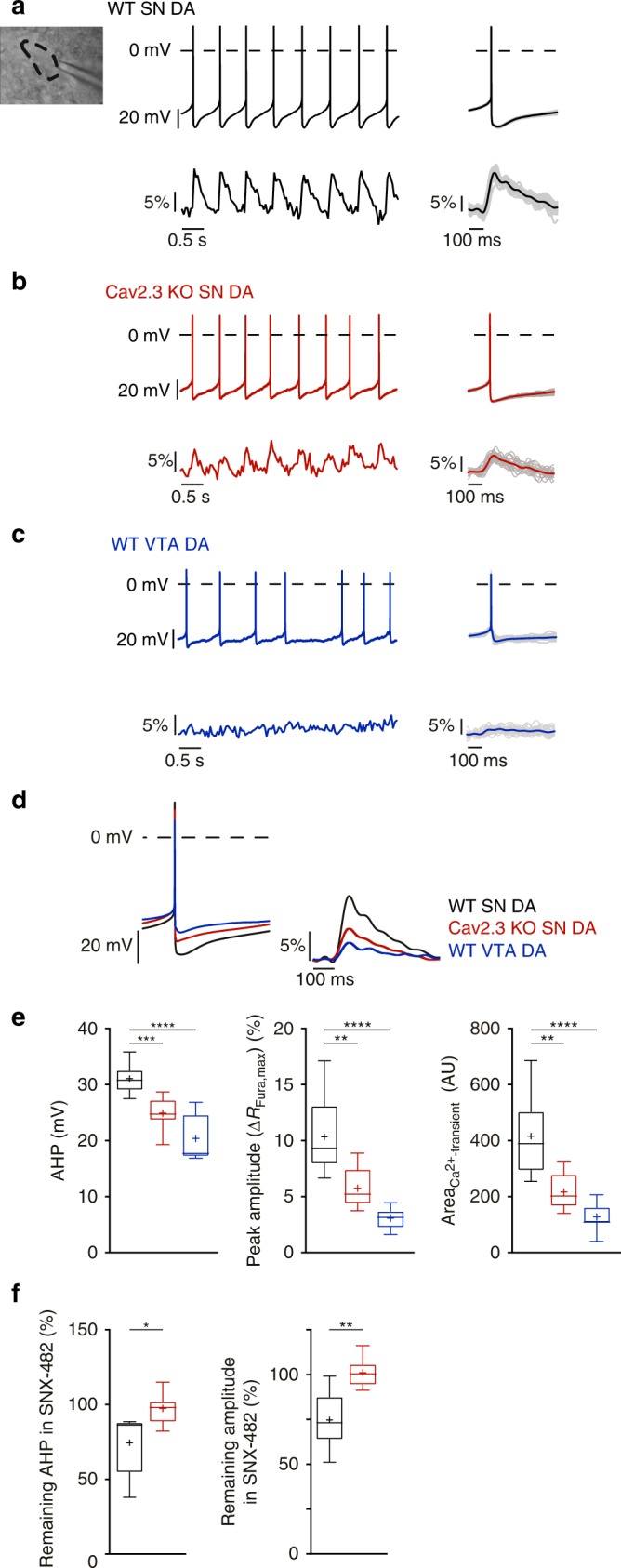
Cav2.3 contributes to somatic action potential-related Ca^2+^ oscillations in adult mouse SN dopaminergic neurons. **a**–**c** Neurons were recorded in the perforated patch-clamp configuration while somatic Ca^2+^ dynamics were simultaneously imaged (insert **a**). Left: Continuous recordings of a wildtype SN dopaminergic (DA) neuron (**a**), Cav2.3 knockout SN dopaminergic neuron (**b**), and wildtype VTA dopaminergic neuron (**c**) illustrating the action potential (AP) firing and the associated Ca^2+^ oscillations. Right: Mean of 20 APs and associated mean Ca^2+^ oscillations for the neurons from the left. Individual traces are superimposed in grey. **d** Average spikes (left) and Ca^2+^ transients (right) of wildtype SN dopaminergic (black trace, *n* = 15), Cav2.3 knockout SN dopaminergic (red trace, *n* = 12), and wildtype mesolimbic VTA dopaminergic (blue trace, *n* = 7) neurons. **e** Plots showing AP after-hyperpolarizations (AHP, left), peak Ca^2+^ amplitudes (as Δ*R*_Fura,max_, middle) and area under the curve (right, AU arbitrary units) of AP-induced Ca^2+^ transients during pacemaking at ~1.5 Hz. **f** Remaining AHP and amplitude of the action potential evoked Ca^2+^ signals (see Methods) in SN dopaminergic neurons of Cav2.3 wildtype (*n* = 6) and Cav2.3 knockout (*n* = 8) mice during the presence of 100 nM SNX-482, relative to those before SNX-482 application. This low concentration likely does not completely inhibit Cav2.3 and was used as SNX-482 can inhibit other channels at higher concentrations. A-type K^+^ currents were blocked throughout the whole experiment by 4 mM 4-AP. Tukey's boxplots are shown. Significances are indicated by asterisks: *p < 0.05, **p < 0.01, ***p < 0.001, ****p < 0.0001. Data values and comparison of all AP parameters are detailed in Supplementary Table 6A–D and Supplementary Figs. 1/2. Source data are available as a Source Data file

In the first approach, we compared somatic Ca^2+^ signals and the electrical properties of SN dopaminergic neurons in brain slices of adult wildtype and Cav2.3 knockout mice. Knockout of Cav2.3 was confirmed by western blotting and immunocytochemical analysis (Supplementary Fig. 6b/d and Supplementary Table 5B). As shown in Fig. 2, SN dopaminergic neurons recorded from wildtype mice displayed spontaneous action potentials as expected. These action potentials were associated with transient rises in cytosolic Ca^2+^ concentration in the soma. In Cav2.3 knockout animals, spontaneous electrical activity of SN neurons was largely unaffected (although we did note small changes in spike waveform, Supplementary Fig. 1 and Supplementary Table 6B). However, the amplitude of the activity-related Ca^2+^ oscillations (at a frequency of ~1.5 Hz) was significantly reduced by ~50% (Fig. 2a, b, d, e and Supplementary Table 6A). VTA dopaminergic neurons from wildtype animals displayed much smaller somatic Ca^2+^ oscillations (Fig. 2c–e and Supplementary Table 6A), in agreement with previous work^13,14^. Ca^2+^-dependent action potential after-hyperpolarizations (AHPs), which are driven in SN dopaminergic neurons by Ca^2+^-sensitive small conductance K^+^ channels (SK)^38–40^, were also significantly reduced in SN dopaminergic neurons of Cav2.3 knockout mice, consistent with the reduction in the Ca^2+^ signals (Fig. 2e, Supplementary Fig. 1c and Supplementary Table 6B).

In a second approach, we examined the effect of acutely inhibiting Cav2.3 with SNX-482. The use of SNX-482 is complicated as it also potently inhibits A-type K^+^ channels in SN dopaminergic neurons^41–43^ and at higher concentrations probably also other Ca^2+^ channels^44^. We therefore blocked K^+^ channels with 4-AP (4 mM), and limited the concentration of SNX-482 to 100 nM, which causes only a partial block of Cav2.3 ^43^. In line with the reduced Ca^2+^ transients upon Cav2.3 deletion, bath application of SNX-482 reduced evoked Ca^2+^ transients by about 25% in wildtype animals (Fig. 2f and Supplementary Table 6C). Like Cav2.3 deficiency, SNX-482 also reduced Ca^2+^-dependent AHPs (Fig. 2f, Supplementary Fig. 2 and Supplementary Table 6D). These SNX effects were specific to Cav2.3 channel inhibition, because SNX-482 was without effect in SN dopaminergic neurons of Cav2.3 knockout mice (Fig. 2f and Supplementary Table 6C/D). In whole-cell voltage-clamp experiments, SNX-482 (100 nM) inhibited voltage-gated Ca^2+^ currents in wildtype mouse SN dopaminergic neurons by ~30% (Supplementary Fig. 3 and Supplementary Table 7).

Taken together, these genetic and pharmacological data identify a significant contribution of Cav2.3 to action potential-associated Ca^2+^ oscillations in the somata of adult SN dopaminergic neurons and to Ca^2+^-dependent AHPs. The established link between activity-related Ca^2+^ oscillations and vulnerability of SN dopaminergic neurons to Parkinson’s disease stressors^13,14,21^ raises the possibility that Cav2.3 contributes to their preferential degeneration.

### Cav2.3 KO protects SN dopaminergic neurons in a Parkinson’s model

**Fig. 3:**
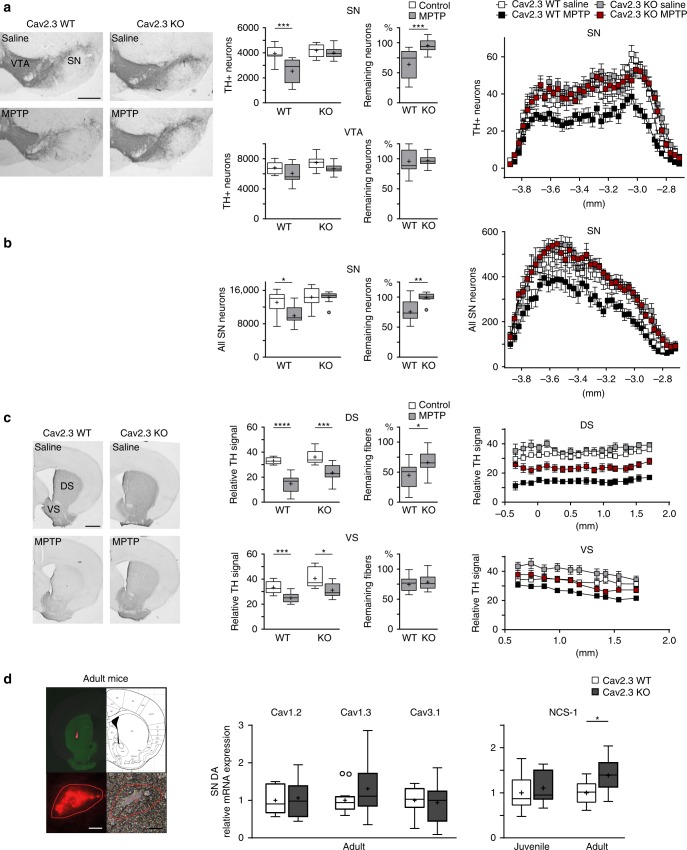
Full and selective protection of SN dopaminergic neurons in Cav2.3 knockout mice in a Parkinson’s disease mouse model. **a** Left: Tyrosine hydroxylase (TH) immunostaining of coronal midbrain sections of Cav2.3 wildtype and Cav2.3 knockout mice, repeatedly treated with MPTP/probenecid or saline as controls, as indicated. Middle: Stereological quantification of SN dopaminergic and VTA dopaminergic neurons (Cav2.3 wildtype saline: *n* = 9; Cav2.3 wildtype MPTP: *n* = 13; Cav2.3 knockout saline: *n* = 9; Cav2.3 knockout MPTP: *n* = 10), and relative remaining neurons in MPTP-treated mice. Right: Mean absolute counted numbers of SN dopaminergic neurons in all analyzed sections for all animals, section position according to bregma. Error bars: SEM. **b** Middle: Automated counting of all SN neurons (Cav2.3 wildtype saline: *n* = 9; Cav2.3 wildtype MPTP: *n* = 12; Cav2.3 knockout saline: *n* = 9; Cav2.3 knockout MPTP: *n* = 10), and relative remaining neurons in MPTP-treated mice. Right: Mean numbers of all SN neurons determined by automated neuron counts for all sections for all animals, section position according to bregma. Error bars: SEM. **c** Left: TH immunostaining of striatal sections of Cav2.3 wildtype and Cav2.3 knockout mice, repeatedly treated with MPTP/probenecid or saline as controls, as indicated. Middle: Optical density quantification of TH signal in dorsal striatum (DS) and ventral striatum (VS) (Cav2.3 wildtype saline: *n* = 9; Cav2.3 wildtype MPTP: *n* = 13; Cav2.3 knockout saline: *n* = 9; Cav2.3 knockout MPTP: *n* = 12), and relative remaining TH signal in MPTP-treated mice. Right: Mean intensities for all analyzed sections for all animals, section position according to bregma. Error bars: SEM. **d** Left: Striatal injection site documentation of an in vivo retrogradely traced adult mouse, aligned with the mouse brain atlas, and a labeled SN dopaminergic neuron, before UV-laser microdissection in fluorescent and brightfield mode. Scale bars: 10 µm. Middle: Cell-specific relative mRNA levels of Cav1.2 (wildtype: *n* = 8; knockout: *n* = 7), Cav1.3 (wildtype: *n* = 16; knockout: *n* = 17) and Cav3.1 (wildtype: *n* = 10; knockout: *n* = 10) in single SN dopaminergic neurons from adult Cav2.3 wildtype and Cav2.3 knockout mice. Right: Cell-specific relative mRNA levels of NCS-1 in single SN dopaminergic neurons from juvenile (wildtype: *n* = 10; knockout: *n* = 10) and adult (wildtype: *n* = 10; knockout: *n* = 10) Cav2.3 wildtype and Cav2.3 knockout mice. Data are given relative to the respective controls. Tukey's boxplots are shown. Significances are indicated by asterisks: *p < 0.05, **p < 0.01, ***p < 0.001, ****p < 0.0001. Data values and additional bootstrapping analysis are detailed in Supplementary Tables 8A/B, 4B/C and Supplementary Figs. 4/5. Source data are available as a Source Data file

We next explored the role of Cav2.3 in neurodegeneration using a neurotoxin-based Parkinson’s disease mouse model. To do this, we compared neuronal viability in wildtype and Cav2.3 knockout mice, subjected to chronic low-dose 1-methyl-4-phenyl-1,2,3,6-tetrahydropyridine (MPTP)/probenecid treatment, which is still the standard model for preclinical testing of neuroprotective Parkinson’s disease therapies in animals^45,46^. We compared the patterns and degrees of neurotoxin-induced loss of SN and VTA dopaminergic midbrain neurons and their axonal striatal terminals by densitometry, unbiased stereology and automated cell counting after tyrosine hydroxylase (as marker for dopaminergic neurons) and hematoxylin immunostaining (Fig. 3, Supplementary Fig. 4a/b/c, and Supplementary Table 8A/B). Knockout of Cav2.3 in SN dopaminergic neurons was confirmed by western blotting and immunocytochemical analysis (Supplementary Fig. 6b/d and Supplementary Table 5B).

We first performed immunostaining of tyrosine hydroxylase and stereology in Cav2.3 knockout mice and wildtype littermates. As shown in Fig. 3a, MPTP treatment resulted in about 40% loss of tyrosine hydroxylase-positive SN dopaminergic neurons from wildtype animals when compared to saline controls. This loss was selective, as MPTP had little effect on numbers of VTA dopaminergic neurons (Fig. 3a and Supplementary Table 8A). In stark contrast, we observed no loss of SN dopaminergic neurons in Cav2.3 knockout animals after MPTP treatment (Fig. 3a and Supplementary Table 8A), thus uncovering a prominent neurodegenerative role for Cav2.3 in SN dopaminergic neurons.

In an independent approach, we performed hematoxylin staining and automated counting of the total neuronal population in the SN (vulnerable dopaminergic and unaffected GABAergic neurons). This analysis confirmed a significant reduction in SN neuron number in the wildtype but not the Cav2.3 knockout animals after MPTP treatment (Fig. 3b, Supplementary Fig. 5b and Supplementary Table 8A).

We also performed densitometric analysis^47^ of tyrosine hydroxylase expression in the dorsal and ventral striatum. These regions harbor the axonal projections of dopaminergic neurons from the SN and VTA, respectively. As shown in Fig. 3c (and Supplementary Table 8B), the tyrosine hydroxylase signal was significantly higher in the dorsal striatum from the Cav2.3 knockout mice compared to wildtype controls after MPTP treatment. In contrast, no such differences were found in the ventral striatum, consistent with Cav2.3 deficiency driving selective protection of highly vulnerable nigro-striatal SN dopaminergic neurons upon neurotoxin insult.

To determine whether the neuroprotective effects of Cav2.3 knockout on SN dopaminergic neurons might be secondary to changes in the functional expression of L-type or T-type voltage-gated Ca^2+^ channels that contribute to dendritic Ca^2+^ oscillations, we quantified Cav1.2, Cav1.3 and Cav3.1 expression in SN dopaminergic neurons (Fig. 3d and Supplementary Table 4B). mRNA levels of all three Cav isoforms were unchanged upon Cav2.3 knockout. In marked contrast, mRNA levels of the neuronal Ca^2+^ sensor NCS-1 were elevated by ~40% in SN dopaminergic neurons from adult but not juvenile Cav2.3 knockout mice (Fig. 3d and Supplementary Table 4C).

Taken together, these data identify Cav2.3 as mediator of SN dopaminergic neuron vulnerability to a degenerative stressor.

### NCS-1 KO enhances SN dopaminergic neuron loss in a Parkinson’s model

The increase in NCS-1 levels in Cav2.3 knockout mice, coupled with our previous study identifying NCS-1 as the functional linker between activity-related Ca^2+^ entry and sensitization of inhibitory dopamine D2-autoreceptors^30^, prompted us to test whether NCS-1 might protect SN dopaminergic neurons from degeneration. We therefore applied the same chronic MPTP Parkinson’s disease model to NCS-1 knockout mice and wildtype controls.

**Fig. 4:**
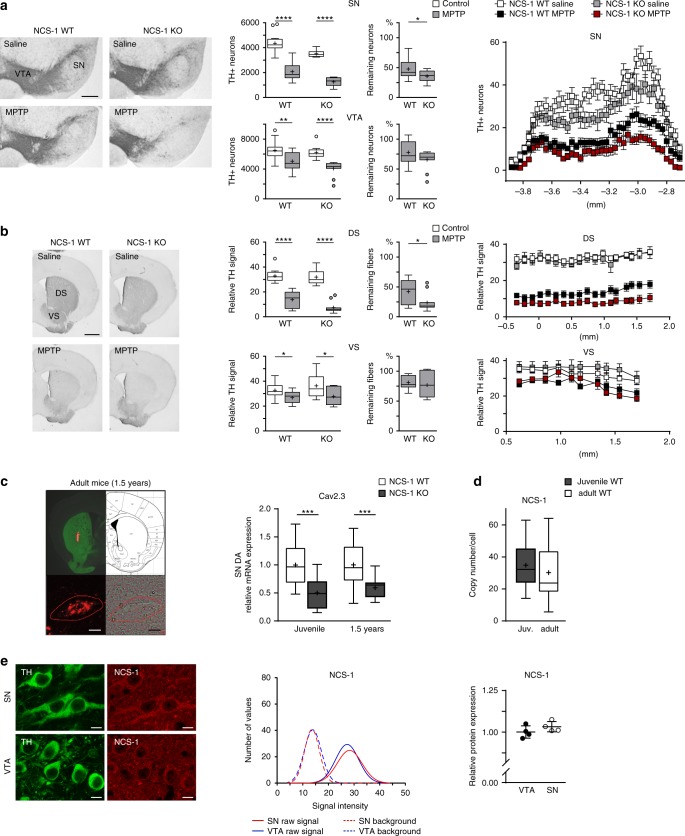
Higher vulnerability of SN dopaminergic neurons from NCS-1 knockout mice in a Parkinson’s disease mouse model. **a** Left: Tyrosine hydroxylase (TH) immunostaining of coronal midbrain sections of NCS-1 wildtype and NCS-1 knockout mice, repeatedly treated with MPTP/probenecid or saline as controls, as indicated. Middle: Stereological quantification of SN dopaminergic and VTA dopaminergic neurons (NCS-1 wildtype saline: *n* = 14; NCS-1 wildtype MPTP: *n* = 13; NCS-1 knockout saline: *n* = 10; NCS-1 knockout MPTP: *n* = 11), and relative remaining neurons in MPTP-treated mice. Right: Mean absolute counted numbers of SN dopaminergic neurons in all analyzed sections for all animals, section position according to bregma. Error bars: SEM. **b** Left: TH immunostaining of striatal sections of NCS-1 wildtype and NCS-1 knockout mice, repeatedly treated with MPTP/probenecid or saline as controls, as indicated. Middle: Optical density quantification of TH signal in dorsal striatum (DS) and ventral striatum (VS) (NCS-1 wildtype saline: *n* = 14; NCS-1 wildtype MPTP: *n* = 13; NCS-1 knockout saline: *n* = 10; NCS-1 knockout MPTP: *n* = 11), and relative remaining TH signal in MPTP-treated mice. Right: Mean intensities for all analyzed sections for all animals, section position according to bregma. Error bars: SEM. **c** Left: Striatal injection site documentation of an in vivo retrogradely traced aged (1.5 years) mouse, aligned with the mouse brain atlas, and a labeled SN dopaminergic neuron, before UV-laser microdissection in fluorescent and brightfield mode. Scale bars: 10 µm. Right: Cell-specific relative mRNA levels of Cav2.3 in single SN dopaminergic neurons from juvenile (wildtype: *n* = 14; knockout: *n* = 20) and aged (1.5 years; wildtype: *n* = 15; knockout: *n* = 14) NCS-1 wildtype and NCS-1 knockout mice. **d** Cell-specific absolute UV-laser microdissection and reverse transcription quantitative PCR-based transcript molecule quantification in single mouse SN dopaminergic neurons for NCS-1 (juvenile: *n* = 13; adult: *n* = 14). **e** Left: Confocal images showing NCS-1 antibody staining (red) of TH-positive (green) neurons in SN and VTA of an adult wildtype mouse, respectively. Scale bar: 10 µm. Middle: Histogram showing the immunosignal intensity distributions of cytoplasmic NCS-1 signal, and respective background signal intensities for all analyzed TH positive SN (red) and VTA (blue) neurons, exemplary for one C57BL/6J mouse (SN: *n* = 157; VTA: *n* = 169). Right: Mean NCS-1 immunosignal quantification in SN and VTA dopaminergic neurons for all analyzed mice (*n* = 4). Antibody specificity was confirmed on NCS-1 knockout mice. Tukey's boxplots are shown. Significances are indicated by asterisks: *p < 0.05, **p < 0.01, ***p < 0.001, ****p < 0.0001. Data values and additional bootstrapping analysis are detailed in Supplementary Tables 8C/D, 4D/E, 5A/B and Supplementary Figs. 4, 6. Source data are available as a Source Data file

Knockout of NCS-1 in SN dopaminergic neurons was confirmed by western blotting and immunocytochemical analysis (Supplementary Fig. 6a/c and Supplementary Table 5B). As shown in Fig. 4a, b (Supplementary Fig. 4d/e/f and Supplementary Table 8C/D), MPTP-induced loss of SN dopaminergic neurons was significantly greater in the NCS-1 knockout mice compared to controls. Similar results were obtained when analyzing axonal projections in the dorsal striatum. In contrast, no differences to MPTP treatment were observed in the VTA or the ventral striatum. These data uncover a region-selective protective effect of NCS-1 on SN dopaminergic neuron viability.

Given the reciprocal effects of Cav2.3 and NCS-1 deficiency on SN dopaminergic neurodegeneration, and the elevated NCS-1 levels in adult SN dopaminergic neurons from Cav2.3 knockout mice, we quantified Cav2.3 mRNA levels in individual SN dopaminergic neurons of NCS-1 knockout and wildtype mice. Intriguingly, Cav2.3 mRNA levels were significantly lower (~50%) in SN dopaminergic neurons of NCS-1 knockout mice compared to those of control mice, suggesting that expressions of Cav2.3 and NCS-1 are linked (Fig. 4c and Supplementary Table 4D). No differences were observed in NCS-1 mRNA levels between young and adult mice (Fig. 4d and Supplementary Table 4E), and NCS-1 protein levels between SN and VTA dopaminergic neurons (Fig. 4e and Supplementary Table 5A).

**Fig. 5:**
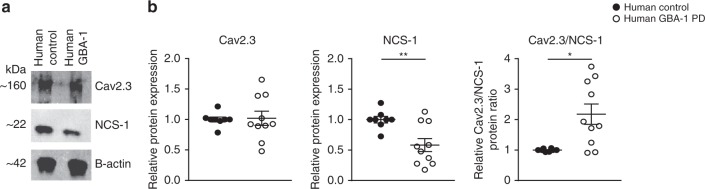
Lower NCS-1 protein expression in human iPSC-derived dopaminergic neurons from Parkinson’s disease patients compared to controls. **a** Western blot analysis of Cav2.3 and NCS-1 (normalized to β-actin) protein levels in iPSC-derived SN dopaminergic-like neurons from control and GBA-1 Parkinson’s disease patients. **b** Cav2.3 and NCS-1 protein expression (relative to that of β-actin), as well as Cav2.3/NCS-1 ratios in iPSC-derived dopaminergic neurons derived from control (*n* = 8) and GBA-1 Parkinson’s disease (*n* = 10) samples. Data were generated from six differentiations in total with four different control lines and three different GBA-1 lines (see Supplementary Table 9A). Antibody specificities were confirmed on respective knockout mice. Error bars: SEM. Significances are indicated by asterisks: *p < 0.05, **p < 0.01, ***p < 0.001, ****p < 0.0001. All data are detailed in Supplementary Table 9A/B and Supplementary Fig. 7. Source data are available as a Source Data file

In our final analysis, we compared NCS-1 and Cav2.3 protein levels in a human model of Parkinson’s disease. For these experiments, we derived dopaminergic neurons from induced pluripotent stem cells (iPSCs) that were obtained from Parkinson’s disease patients heterozygous for a point mutation (N370S) in the GBA gene coding for a lysosomal glucocerebrosidase (Supplementary Table 9A)^48^. Mutations in this gene constitute the highest genetic risk factor associated with idiopathic Parkinson’s disease^49,50^. Differentiated neurons were positive for class III beta-tubulin and for tyrosine hydroxylase and showed the typical pacemaker-activity of dopaminergic neurons (Supplementary Fig. 7)^51–53^. Both, NCS-1 and Cav2.3 proteins were readily detectable in these neurons by western blotting (Fig. 5a). Specificity of antibodies was confirmed using the respective knockout mice (Supplementary Fig. 6a and refs. ^54–56^). For Cav2.3, no significant difference in protein expression was detected between human dopaminergic neurons from healthy controls and Parkinson’s disease patients (Fig. 5b and Supplementary Table 9B). In contrast, NCS-1 protein levels were about 40% lower in the diseased neurons (Fig. 5b and Supplementary Table 9B).

NCS-1 thus emerges as protective factor during SN dopaminergic degeneration, of likely relevance to Parkinson’s disease.

## Discussion

Here, we identify Cav2.3 as mediator of SN dopaminergic neuron loss in an in vivo model of Parkinson’s disease. Together with an identified protective role of NCS-1 for these neurons, our data highlight de-regulated Ca^2+^ signaling as a culprit in the disease. Cav2.3 and NCS-1 thus emerge as potential targets for neuroprotective therapy.

Voltage-gated Ca^2+^ entry is a key determinant of neuronal function. Ca^2+^ entry during pacemaking in vulnerable but not in resistant dopaminergic neurons is coupled to Ca^2+^ uptake by mitochondria, and to ATP production, particularly under increased metabolic demand^21,36,57^. Such homeostasis sustains neuronal activity, dopamine release and thus movement, but it comes at a metabolic cost. Thus, SN dopaminergic neurons, with their activity-related Ca^2+^ oscillations, and their large, arborized axonal structures, are energetically living on the edge^36,58,59^. Parkinson’s disease stressors such as aging, mitochondrial complex-I dysfunction, or mutations in PARK genes likely disrupt this delicate balance and tip them over the edge^1,36,58^. Such a scenario might explain the higher vulnerability of SN dopaminergic neurons to degeneration compared to those in the VTA in Parkinson’s disease^1,36^. Thus, understanding the exact sources of activity-related Ca^2+^ load and the downstream effectors in dopaminergic neurons is key to understanding how their viability is maintained.

Our data show that Cav2.3 is the most abundantly expressed voltage-gated Ca^2+^ channel subtype in adult SN dopaminergic neurons. This channel has not yet been linked with metabolic stress in these neurons or with their degeneration in Parkinson’s disease. Our data using both, Cav2.3 knockout mice and the Cav2.3 inhibitor SNX-482, indicate that Cav2.3 contributes significantly to Ca^2+^ fluxes and Ca^2+^ currents upon pacemaking in the somata of SN dopaminergic neurons. SNX-482 inhibits voltage-gated A-type K^+^ channels with similar affinity as to Cav2.3^43^, and these channels are functionally expressed in SN dopaminergic neurons^41,42^. But A-type channel blockade is unlikely to contribute to observed inhibition of Ca^2+^ signals by SNX-482, because (i) experiments were performed in the presence of 4-amino-pyridine, which fully inhibits A-type K^+^ channels and (ii) we observed no inhibition of Ca^2+^ signals by SNX-482 in SN dopaminergic neurons from Cav2.3 knockout animals. Therefore, the congruent effects of knockout and pharmacological block of Cav2.3 leads us to conclude that the observed effects in the Cav2.3 knockout mice are rather caused by the loss of the functional channel than compensatory mechanisms.

Previous work showed that Ca^2+^ transients in SN dopaminergic neurons were inhibited by the L-type voltage-gated Ca^2+^ channel blocker isradipine and Cav1.3 shRNA by about 50%^14^. This is similar to the degree of inhibition we observed here upon interfering with Cav2.3. T-type Ca^2+^ channels also contribute to Ca^2+^ fluxes in SN dopaminergic neurons^14^ and to rotenone-induced cell death in iPSC-derived dopaminergic neurons^23,25^. Notably, these previous Ca^2+^ recordings were made in dendrites^13,14^ whereas our measurements were made in the soma, pointing strongly to subcellular differences in Ca^2+^ channel activity in SN dopaminergic neurons. Indeed, somatic Ca^2+^ transients are only modestly sensitive to isradipine^37^. Of relevance, L-type Ca^2+^ channel blockers failed to protect neurons in the latter model of Parkinson’s^23^, and they also failed in modifying disease progression in human Parkinson’s patients^20^.

SNX-482-sensitive currents have recently been described in juvenile rat SN dopaminergic neurons^27^ contributing to ~10% of voltage-gated Ca^2+^ currents. In adult mouse SN dopaminergic neurons, the relative contribution of Cav2.3 remains unclear. But our experiments showing an age-dependent upregulation of Cav2.3 expression (Fig. 1) coupled with the reported downregulation of L-type expression^37^ and reduction of L-type currents in SN dopaminergic neurons^60^ suggest a significant role for R-type currents. Currently, it remains also unclear to which extent Cav-mediated Ca^2+^ oscillations^13,14,21^ in dopaminergic neurons are coupled and amplified by Ca^2+^-induced Ca^2+^ release from intracellular Ca^2+^ stores^61^. Cav2.3 and RyR3 are functionally coupled in pancreatic delta-cells^62^, raising the possibility that this may also occur in neurons.

Independent evidence that Ca^2+^ fluxes in SN dopaminergic neurons are de-regulated upon Cav2.3 knockout or pharmacological Cav2.3 inhibition stems from our observations that Ca^2+^-dependent AHPs are correspondingly reduced. These AHPs in SN dopaminergic neurons are sensitive to inhibition of small conductance K^+^ channels^40,63^. Our data strongly suggest functional coupling with Cav2.3 channels. This is similar to coupling of Cav2.3 and big conductance K^+^ channels underlying AHPs in hippocampal pyramidal neurons^64^.

Pacemaking in VTA dopaminergic neurons was associated with only modest Ca^2+^ signals in the somata (similar to that described for dendrites^14^) and small AHPs. Thus, loss or blockade of Cav2.3 shifts the properties of dopaminergic neurons in the SN that are vulnerable in Parkinson’s disease toward those in the VTA which are resistant^13,40,65^.

We used the chronic MPTP/probenecid Parkinson’s disease model (injection interval 3.5 days) to avoid acute toxicity, described for other (acute/subacute) MPTP protocols. The toxicity mechanism of MPTP and MPP^+^ (the neurotoxic metabolite) has been extensively studied^66,67^. MPP^+^ inhibits the complex I of the mitochondrial respiratory chain and thus mimics the complex I deficiency, described for mitochondria in human SN dopaminergic neurons from Parkinson’s patients^66,68^.

Although our transgenic mouse model identifies a clear phenotype upon Cav2.3 loss, possible compensation is important to consider particularly for global knockouts. Indeed, we had described that loss of Cav1.3 upon knockout in mice is functionally compensated by upregulation of Cav3.1 in SN dopaminergic neurons^22^. Here, we found no such evidence for compensatory increases in Cav3.1, Cav1.2 or Cav1.3 mRNA levels in SN dopaminergic neurons of Cav2.3 knockout mice. Thus, the neuroprotection we observe in these animals is unlikely to be explained by changes in expression of other Ca^2+^ channels.

We did however detect an increase in levels of NCS-1 mRNA in SN dopaminergic neurons from Cav2.3 knockout mice. Notably, this increase in Cav2.3 knockout mice was manifest only in SN dopaminergic neurons from adult mice and thus age-dependent. In contrast, NCS-1 levels remained stable in wildtype mice, both age-dependent and region-selective. Ca^2+^-dependent regulation of NCS-1 expression has been described in yeast^69^. We hypothesize that functional expression of protective NCS-1 in SN dopaminergic neurons might get upregulated in response to Parkinson’s disease stressors. This view is supported, as NCS-1 stimulates mitochondrial function and neuronal survival promotion in general^70,71^, and as NCS-1 mRNA levels are elevated in remaining human SN dopaminergic neurons from post mortem Parkinson’s disease brains^30^. In contrast, the lower NCS-1 levels detected in iPSC-derived dopaminergic neurons from GBA-1 Parkinson’s disease patients, offers an explanation for their accelerated degeneration.

Previous work suggested that NCS-1 function in SN dopaminergic neurons could have neuroprotective effects^30–32^. Consistent with this, knockout of NCS-1 resulted in more severe dopaminergic neuronal loss in our Parkinson’s disease model. Importantly, this elevated loss was restricted to the SN, despite global NCS-1 knockout. Thus, elevated levels of NCS-1 in SN dopaminergic neurons of adult Cav2.3 knockout mice might contribute to the observed neuroprotection.

We also found that SN dopaminergic neurons from NCS-1 knockout mice displayed lower Cav2.3 mRNA levels than wildtype mice. We speculate that the Cav2.3 downregulation reflects a compensatory response to combat Cav2.3-mediated degeneration in NCS-1-depleted SN neurons. In this context, Cav2.3 mRNA levels are also lowered upon overexpression of mutant human α-synuclein (PARK1) in dorsal motor nucleus tissue of the vagus nerve^72^. Cholinergic neurons in this area display Ca^2+^-associated pacemaker activity, similar to SN dopaminergic neurons, and Lewy-body protein aggregates in Parkinson’s disease. But for unknown reasons, these neurons are less vulnerable to degeneration^3,72,73^. Based on our findings, downregulation of Cav2.3 in response to Parkinson’s disease stressors offers a feasible explanation.

Collectively, our data strongly suggest opposing roles for Cav2.3 and NCS-1 in Parkinson’s disease. Cav2.3 is neurodegenerative whereas NCS-1 is protective for SN dopaminergic neurons. Whether this involves any direct functional or molecular interactions between the two proteins must be clarified in future experiments.

Importantly, NCS-1 expression is reduced in iPSC-derived dopaminergic neurons from familial Parkinson’s disease patients heterozygous for the N370S mutation in the *GBA1* gene. Previous studies using iPSC-derived dopaminergic neurons from Parkinson’s patients with *GBA1* mutations identified disrupted Ca^2+^ homeostasis and increased vulnerability to stress responses that were rescued by isogenic correction^74^. Age-dependent dysregulation of Ca^2+^ homeostasis has also been described in patient fibroblasts carrying the N370S mutation in the GBA gene^48^. Notably, NCS-1 levels are higher in the surviving dopaminergic neurons from post mortem idiopathic Parkinson’s disease patients^30^. We suggest that the relative activity of Cav2.3 and NCS-1 within the complex Ca^2+^ signaling and PARK gene network in SN dopaminergic neurons may contribute to define their viability during stress.

Although epidemiological evidence links use of L-type Ca^2+^ channel blockers to reduced risk of Parkinson’s disease, isradipine afforded no protection in Parkinson’s patients in a recently concluded phase III clinical trial^20^. Plasma levels reached during treatment with a maximal tolerable dose of isradipine might have been insufficient to fully inhibit L-type voltage-gated Ca^2+^ channels in SN dopaminergic neurons^21,37^. Alternatively, inhibition of L-type voltage-gated Ca^2+^ channels might preferentially be protective only under distinct conditions, e.g. before motor symptoms manifest, or in response to transiently elevated dopamine levels^30^ during dopamine replacement therapy^6,21^. Inhibition of Cav2.3—alone or in combination with inhibition of L-type and/or T-type voltage-gated Ca^2+^ channels^14,23^—could form the basis of a neuroprotective strategy for Parkinson’s disease in the future. However, the only available Cav2.3 inhibitor (SNX-482) is unsuitable for neuroprotection in a clinical setting due to off target effects^28,43,75^. Thus, development of high affinity, brain-permeable, and selective Cav2.3 channel blockers is warranted^28,75^.

Detailed information for all methodological approaches are given in the Supplementary Methods.

### Reporting summary

Further information on research design is available in the Nature Research Reporting Summary linked to this article.

## Supplementary information

Supplementary Information

Reporting Summary

Source Data

## Data Availability

All data generated or analyzed during this study are included in this published article and Supplementary Information file, or available from the authors upon request. Source data underlying Figs. 1–5 are available as a Source Data file.
