# Supplementary material for: Cav2.3 channels contribute to dopaminergic neuron loss in a model of Parkinson’s disease
Source: Nat Commun. 2019 Nov 8;10:5094. doi: 10.1038/s41467-019-12834-x (PMC6841684; doi:10.1038/s41467-019-12834-x)
Supplement: Supplementary file 1 — Supplementary Information [file 41467_2019_12834_MOESM1_ESM.pdf]

# Supplementary Information

# Cav2.3 channels contribute to dopaminergic neuron loss in a model of Parkinson’s disease

**Benkert et al.**

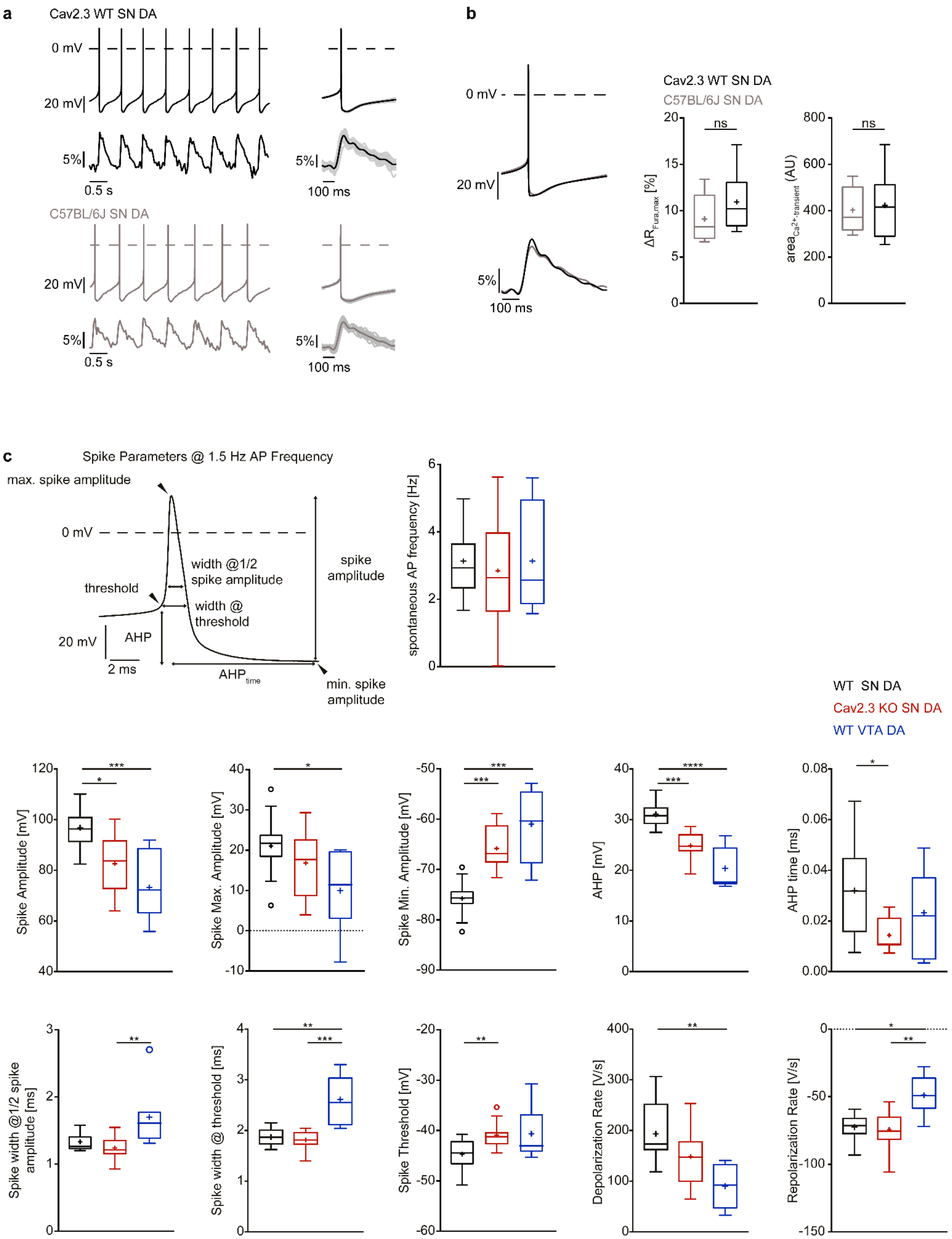
a
Cav2.3 WT SN DA
0 mV
20 mV
5%
0.5 s
100 ms
C57BL/6J SN DA
20 mV
5%
0.5 s
100 ms
b
0 mV
20 mV
5%
100 ms
Cav2.3 WT SN DA
C57BL/6J SN DA
ns
ns
$\Delta R_{Fura,max}$ [%]
$area_{Ca^{2+}\text{-}transient}$ (AU)
c
Spike Parameters @ 1.5 Hz AP Frequency
max. spike amplitude
0 mV
width @1/2 spike amplitude
threshold
width @ threshold
spike amplitude
20 mV
AHP
2 ms
$AHP_{time}$
min. spike amplitude
spontaneous AP frequency [Hz]
WT SN DA
Cav2.3 KO SN DA
WT VTA DA
Spike Amplitude [mV]
Spike Max. Amplitude [mV]
Spike Min. Amplitude [mV]
AHP [mV]
AHP time [ms]
Spike width @1/2 spike amplitude [ms]
Spike width @ threshold [ms]
Spike Threshold [mV]
Depolarization Rate [V/s]
Repolarization Rate [V/s]

**Supplementary Figure 1.**

**Action potential parameters of mouse SN dopaminergic neurons, analyzed in $Ca^{2+}$ imaging experiments. a, Left:** Continuous recordings of a Cav2.3 wildtype littermate SN dopaminergic neuron (upper), and C57BL/6J SN dopaminergic (lower) illustrating the action potential (AP) firing and the associated $Ca^{2+}$ oscillations. **Right:** Mean of 20 APs and associated mean $Ca^{2+}$ oscillations for the neurons from the left. Individual traces are superimposed in grey. **b, Left:** Average spikes (upper) and $Ca^{2+}$ transients (lower) of SN dopaminergic neurons from Cav2.3 wildtype littermate (black trace, n=10), and C57BL/6J mice (grey trace, n=5). **Right:** Summary plots showing the peak amplitudes and area under the curves of action potential-induced $Ca^{2+}$ oscillations during pacemaking at ~1.5 Hz. **c, Insert:** Schematic representation of analyzed $Ca^{2+}$ dependent and $Ca^{2+}$ independent action potential parameters. **Graphs:** Analysis of action potential parameters as indicated in SN dopaminergic neurons of wildtype (black trace, n=15) and Cav2.3 knockout (red trace, n=12), as well as wildtype VTA dopaminergic neurons (blue trace, n=7). Tukeys boxplots are shown. Significances are indicated by asterisks: * $p < 0.05$ ** $p < 0.01$, *** $p < 0.001$, **** $p < 0.0001$. All data and statistics are detailed in Supplementary Table 6A/B.

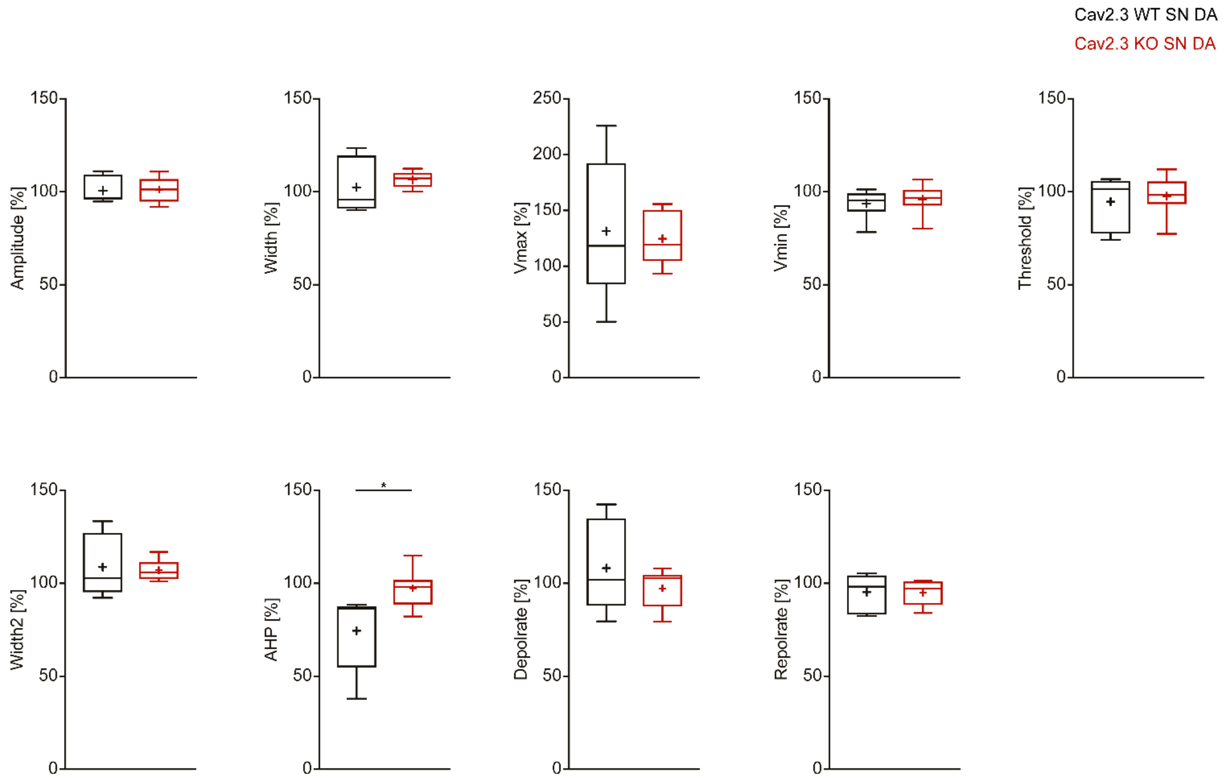


**Supplementary Figure 2.**

**Relative changes in evoked spike parameters of mouse SN dopaminergic neurons in SNX-482, analyzed in $Ca^{2+}$ imaging experiments.** Analysis of evoked spike parameters as indicated in SN dopaminergic neurons of Cav2.3 wildtype (n=6) and Cav2.3 knockout mice (n=8). Significances are indicated by asterisks: * $p < 0.05$ ** $p < 0.01$, *** $p < 0.001$, **** $p < 0.0001$. Tukeys boxplots are shown. All data and statistics are detailed in Supplementary Table 6D.

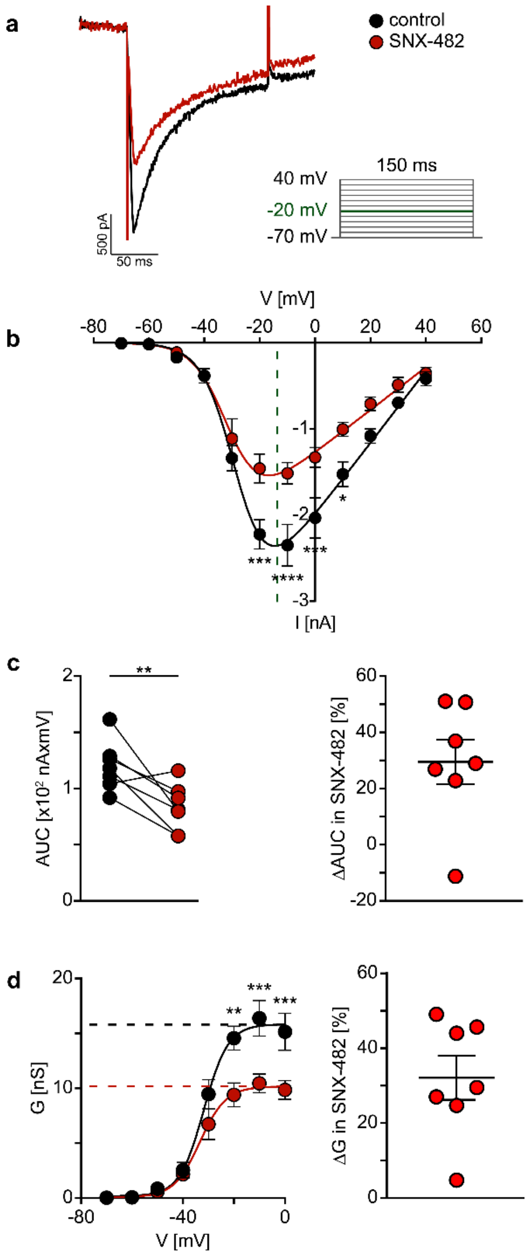


**Supplementary Figure 3.**

**SNX-482 inhibits about 30% of voltage activated $Ca^{2+}$ currents in mouse SN dopaminergic neurons. a,** Exemplary traces at -20 mV of voltage step protocol (insert) recordings before and in SNX-482 (100 nM). ACSF solution contained in mM: 125 NaCl, 25 NaHCO3, 2.5 KCl, 1.25 NaH2PO4, 2.058 MgCl2, 1.8 CaCl2, 2.5 glucose, 5 CsCl, 15 Tetraethylammonium, 2.5 4-Aminopyridine, 600 nM TTX, 20 µM CNQX, 4 µM SR 95531, and 10 µM DL-AP5. Internal solution: 180 N-Methyl-D-glucamine, 40 HEPES, 0.1 EGTA, 4 MgCl2, 5 Na-ATP, 1 Lithium-GTP, 0.1% neurobiotine. **b,** Current

to voltage (I-V) relation analysis (fit of mean values) of SN DA neurons before and in SNX-482 (n=7). Dotted line indicates mV at maximal current. **c,** Area under the I-V curve (AUC) before and in SNX-482 (left), and ΔAUC for each individual SN DA neuron (right). **d,** Mean maximal conductance (G) before and in SNX-482 (fit of mean values, left) and ΔG for each individual SN dopaminergic neuron (right). Error bars: SEM. Significances in (b+c) were determined using 2-way ANOVA with Sidak's multiple comparison test, in (d) according to Mann-Whitney U test, and are indicated by asterisks: * $p < 0.05$, ** $p < 0.01$, *** $p < 0.001$, **** $p < 0.0001$. All data are detailed in Supplementary Table 7.

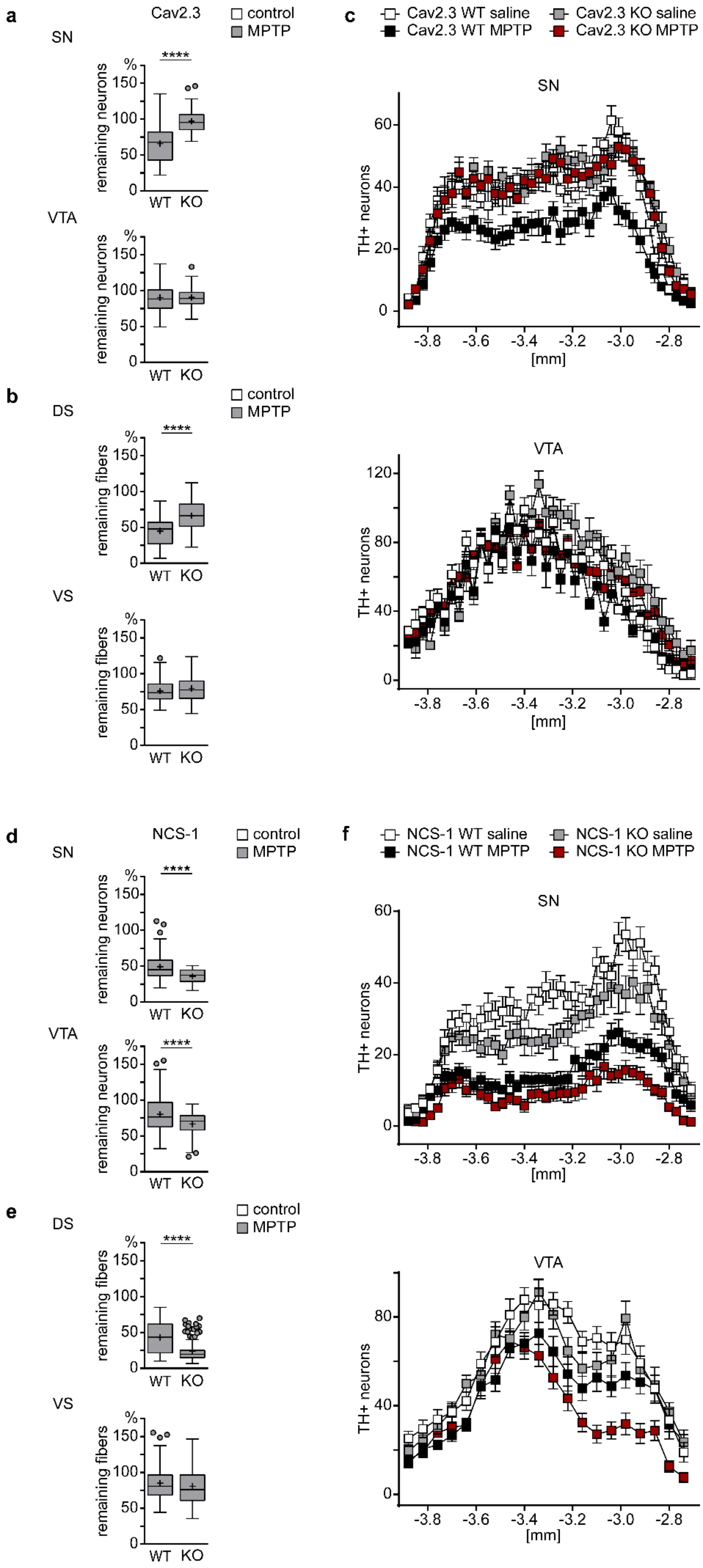

a
Cav2.3
control
MPTP
SN
****
remaining neurons
WT KO
VTA
remaining neurons
WT KO
b
control
MPTP
DS
****
remaining fibers
WT KO
VS
remaining fibers
WT KO
c
Cav2.3 WT saline
Cav2.3 WT MPTP
Cav2.3 KO saline
Cav2.3 KO MPTP
SN
TH+ neurons
[mm]
VTA
TH+ neurons
[mm]
d
NCS-1
control
MPTP
SN
****
remaining neurons
WT KO
VTA
****
remaining neurons
WT KO
e
control
MPTP
DS
****
remaining fibers
WT KO
VS
remaining fibers
WT KO
f
NCS-1 WT saline
NCS-1 WT MPTP
NCS-1 KO saline
NCS-1 KO MPTP
SN
TH+ neurons
[mm]
VTA
TH+ neurons
[mm]

**Supplementary Figure 4.**

**Full and selective protection of SN dopaminergic neurons from degeneration in Cav2.3 knockout mice, and higher vulnerability of SN dopaminergic neurons in NCS-1 knockout mice, in the chronic neurotoxin Parkinson's disease mouse-model (MPTP). a/d,** Relative mean remaining numbers of SN and VTA dopaminergic neurons in MPTP treated animals, with respect to mean signals of respective saline treated animal groups, with bootstrapping analysis approach (Cav2.3 wildtype MPTP: n=117; Cav2.3 knockout MPTP: n=90; NCS-1 wildtype MPTP: n=182; NCS-1 knockout MPTP: n=110). **b/e,** Mean of relative remaining TH signals in DS and VS in MPTP treated animals. Signals were normalized to mean signals of respective saline treated animal groups, with bootstrapping analysis approach (Cav2.3 wildtype MPTP: n=117; Cav2.3 knockout MPTP: n=108; NCS-1 wildtype MPTP: n=182; NCS-1 knockout MPTP: n=110). Tukeys boxplots are shown **c/f,** Mean absolute counted numbers of SN and VTA dopaminergic neurons for all analyzed sections for all animals, section position according to bregma. Error bars: SEM. Significances are indicated by asterisks: * $p < 0.05$ ** $p < 0.01$, *** $p < 0.001$, **** $p < 0.0001$. All data and statistics are detailed in Supplementary Table 8A-D.

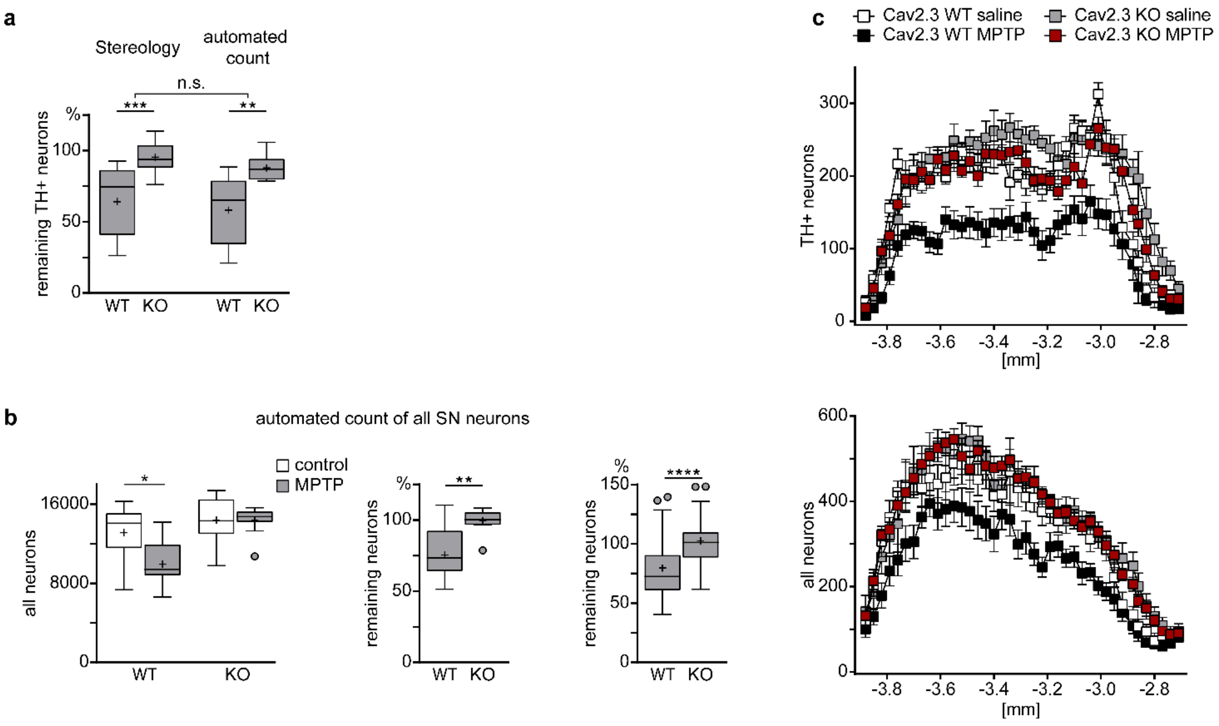


**Supplementary Figure 5.**

**Automated cell counting confirms full and selective protection of SN neurons from degeneration in Cav2.3 knockout mice in the chronic neurotoxin Parkinson's disease mouse-model (MPTP). a,** Remaining TH-positive SN neurons in Cav2.3 wildtype and Cav2.3 knockout mice after chronic MPTP, analyzed via unbiased stereology (estimate; Cav2.3 wildtype: N=13; Cav2.3 knockout: N=10) and via automated cell count after algorithm training (absolute neuron number count; Cav2.3 wildtype: N=12; Cav2.3 knockout: N=10). Detected MPTP-induced SN dopaminergic neuron loss in Cav2.3 wildtype as well as in Cav2.3 knockout mice is similar in both approches. **b, Left:** Automated count of all hematoxylin stained SN neurons (dopaminergic and GABAergic) in saline and MPTP treated Cav2.3 wildtype and Cav2.3 knockout mice (Cav2.3 wildtype saline: N=9; Cav2.3 wildtype MPTP: N=12; Cav2.3 knockout saline: N=9; Cav2.3 knockout MPTP: N=10) detects a significant MPTP induced loss of SN neurons only in Cav2.3 wildtype but not in Cav2.3 knockout mice. **Middle/right:** Remaining numbers of SN neurons in MPTP treated Ca2.3 wildtype and Cav2.3 knockout mice, in respect to respective saline treated animal groups, without (middle; Cav2.3 wildtype MPTP: N=12; Cav2.3 knockout MPTP: N=10) and with

bootstrapping analysis (right; Cav2.3 wildtype MPTP: n=108; Cav2.3 knockout MPTP: n=90). Tukeys bosplots are shown. **c,** Numbers of TH-positive SN neurons (upper) and all SN neurons (lower) for all analyzed sections for all animals plotted over the caudo-rostral axis, section position according to bregma. Neuron numbers were determined via automated counting. Error bars: SEM. Significances are indicated by asterisks: * $p < 0.05$ ** $p < 0.01$, *** $p < 0.001$, **** $p < 0.0001$. All data and statistics are detailed in Supplementary Table 8A.

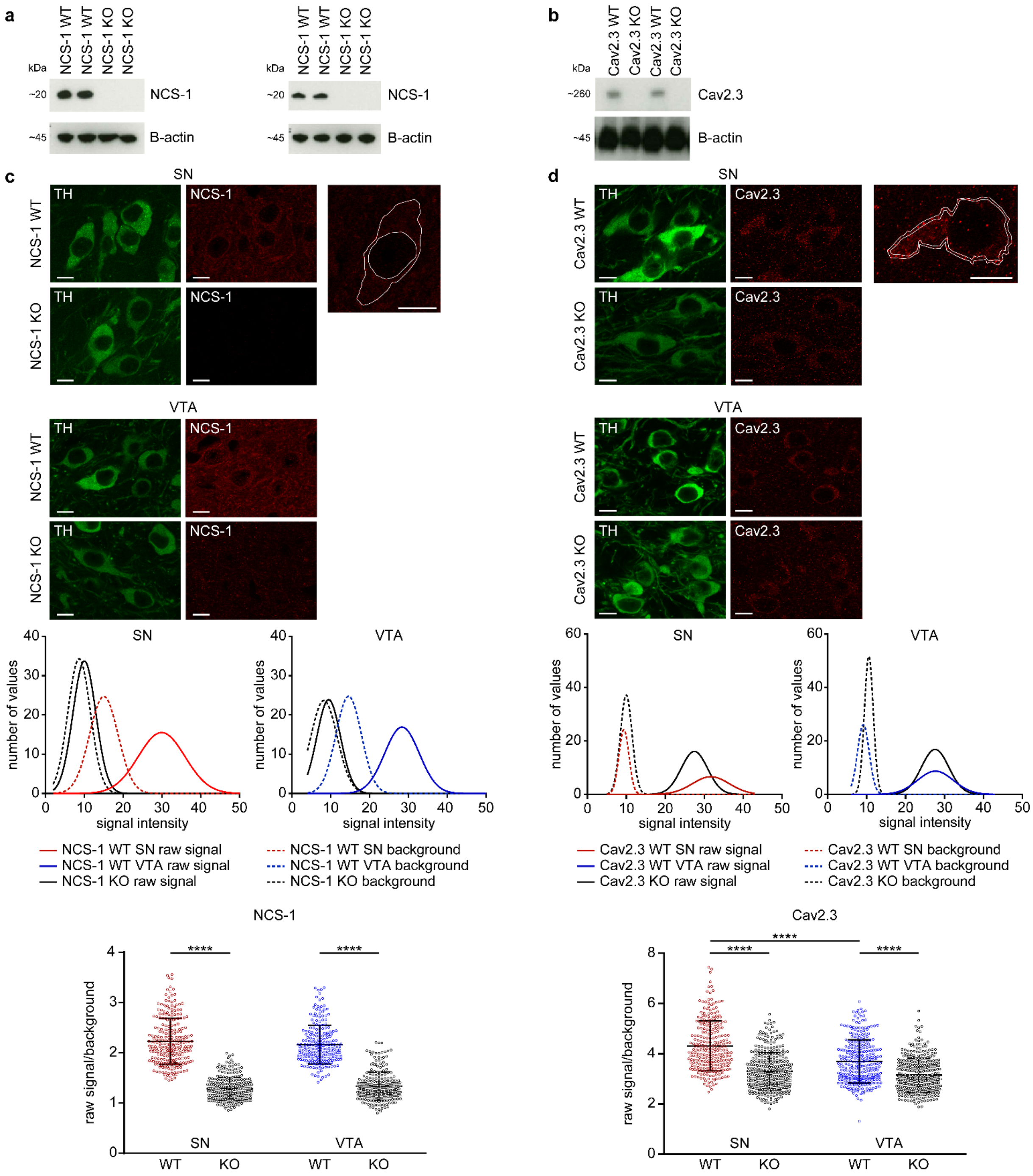


**Supplementary Figure 6.**

**Specificity-test of all NCS-1 and Cav2.3 antibodies used in this study for semi-quantitative analysis by using the respective wildtype and knockout mice. a/b,** Representative Western blots of NCS-1 (a; 10506-2-AP Proteintech, left, and abcam ab129166, right) and Cav2.3 (b; 27225-1-

AP Proteintech) antibodies used for staining shown in Fig. 1c, Fig. 4e and Fig. 5. Tests were performed by using whole brain protein lysates from NCS-1 wildtype (N=2) and NCS-1 knockout (N=4) as well as Cav2.3 wildtype (N=3) and Cav2.3 knockout (N=3) mice (see supplementary online methods for details). **c, Upper:** Representative confocal images showing NCS-1 (10506-2-AP Proteintech) antibody staining (red) of TH-positive (green) neurons in SN and VTA of an adult NCS-1 wildtype and NCS-1 knockout mice, respectively. High magnification of one dopaminergic neuron from left shows the marked region of interest (ROI) defined as cytoplasm. Scale bar: 10 µm. **Lower:** Representative histograms showing the immunosignal intensity distributions of cytoplasmic NCS-1 signal, and respective background signal intensities for all analyzed TH-positive SN (red, left) or VTA (blue, right) dopaminergic neurons, exemplary for one NCS-1 wildtype and one NCS-1 knockout mouse. Scatter plots summarize data for all analyzed animals (N=3 each, SN NCS-1 wildtype: n=257, SN NCS-1 knockout: n=263, VTA NCS-1 wildtype: n=226, VTA NCS-1 knockout: n=240) expressed as ratio between raw signal and background on NCS-1 wildtype and NCS-1 knockout for both regions. Wildtype and knockout sections were processed in parallel, and SN and VTA neurons analysis was performed on the same sections (Note that signals in TH-positive neurons from wildtype are significantly higher than in knockout, indicating the specificity of the analyzed signal in Fig. 4e). **d, Upper:** Representative confocal images showing Cav2.3 antibody staining (red) of TH-positive (green) neurons in SN and VTA of an adult Cav2.3 wildtype and Cav2.3 knockout mice. High magnification of one dopaminergic neuron from left shows the marked region of interest (ROI) defined as membrane signal. Scale bar: 10 µm. **Lower:** Representative histograms showing the immunosignal intensity distributions of plasma membrane Cav2.3 signal, and respective background signal intensities for all analyzed TH-positive SN (red, left) or VTA (blue, right) dopaminergic neurons, exemplary of one Cav2.3 wildtype and one Cav2.3 knockout mouse. Scatter plots summarize data for all analyzed animals (N=3 each, SN Cav2.3 wildtype: n=264, SN Cav2.3 knockout: n=344, VTA Cav2.3 wildtype: n=287, VTA Cav2.3 knockout: n=346) expressed as ratio between raw signal and background on Cav2.3 wildtype and Cav2.3 knockout for both regions. Wildtype and knockout sections were processed in parallel, and SN and VTA neurons analysis was performed in parallel on the same sections (Note that signals in TH-positive neurons from wildtype are significantly higher than in knockout, indicating the specificity of the analyzed signal in Fig. 1c). Error bars: SD. Significances are indicated by

asterisks: * $p < 0.05$ ** $p < 0.01$, *** $p < 0.001$, **** $p < 0.0001$. All data and statistics are detailed in Supplementary Table 5B.

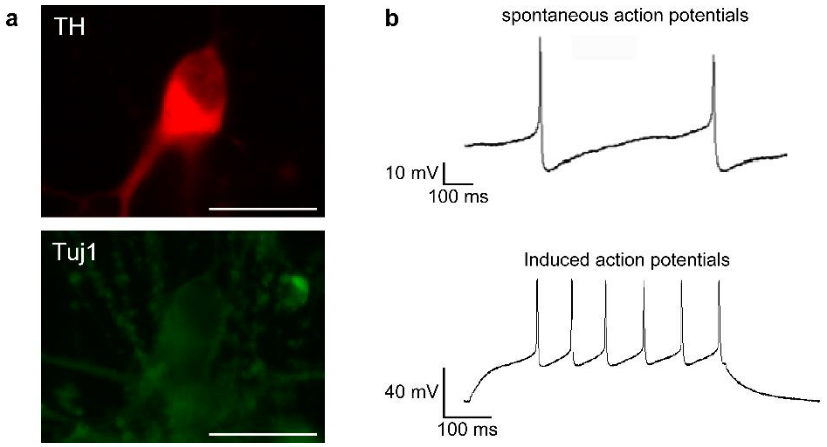


**Supplementary Figure 7.**

**Phenotypic identity of human iPSC-derived, differentiated dopaminergic neurons. a,** Representative immunofluorescence images of a differentiated dopaminergic neuron in culture (at 50 days *in vitro*) showing expression of the dopaminergic neuron marker tyrosine hydroxylase (TH, red), and the neuronal marker class III beta-tubulin (Tuj1, green). Scale bars: 20 µm. **b,** Representative electrophysiological whole cell current-clamp recording of an iPSC derived differentiated TH- and Tuj1-positive neuron, showing the typical action potential waveform and low-frequency activity of SN dopaminergic neurons (upper: spontaneous pacemaker activity; lower: action potentials upon injection of +40 pA current from a holding potential of -70 mV).

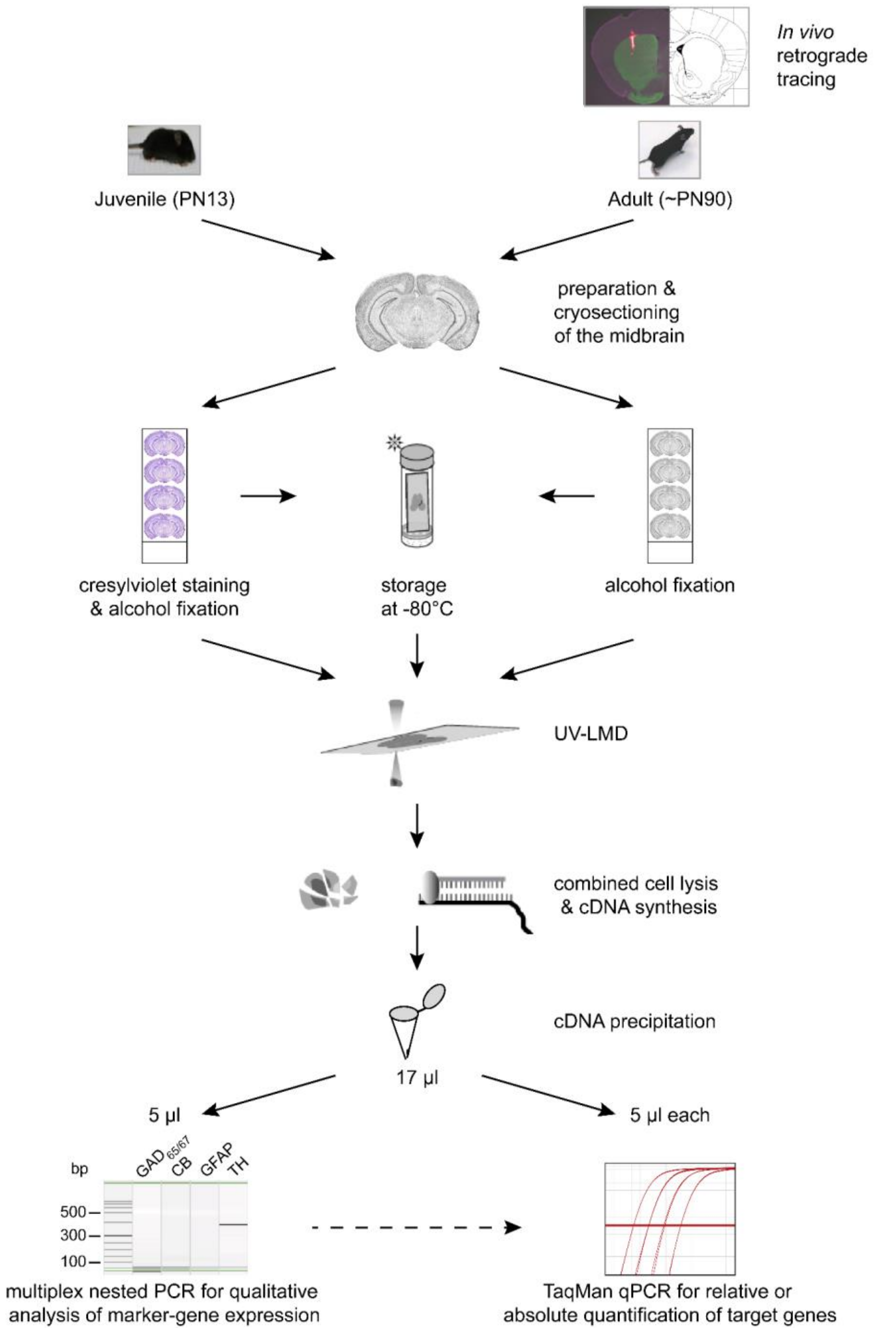


**Supplementary Figure 8.**

**General workflow of UV-LMD & RT-qPCR analysis**. SN dopaminergic neurons of adult mice were retrogradely traced with fluorescent retrobeads *in vivo*. Midbrains were prepared and cut into 12 µm coronal cryosections, fixed with ethanol and - for juvenile mice - stained with cresyl-violet

(CV). 10 individual SN neurons were isolated using UV-LMD. After combined cell lysis and cDNA synthesis, cDNA samples were purified via ethanol precipitation[1] and dissolved in 17 µl water (5Prime). To verify the homogeneity of each sample, 5 µl cDNA (equivalent to ~3 cells) was used for a multiplex nested PCR of a panel of positive and negative marker genes[2-4]. Only samples expressing the correct marker gene profile (positive for TH and negative for GAD65/67, CB and GFAP) were further analyzed via TaqMan qPCR. For each TaqMan qPCR, 5 µl of a cDNA sample was used as template (equivalent to ~3 cells). Relative or absolute expression levels were determined by running respective standard curve DNA samples in parallel (see next paragraph, cited methods papers, and cartoon (B) for further details). Workflow modified from[5], graphics created by Julia Benkert.

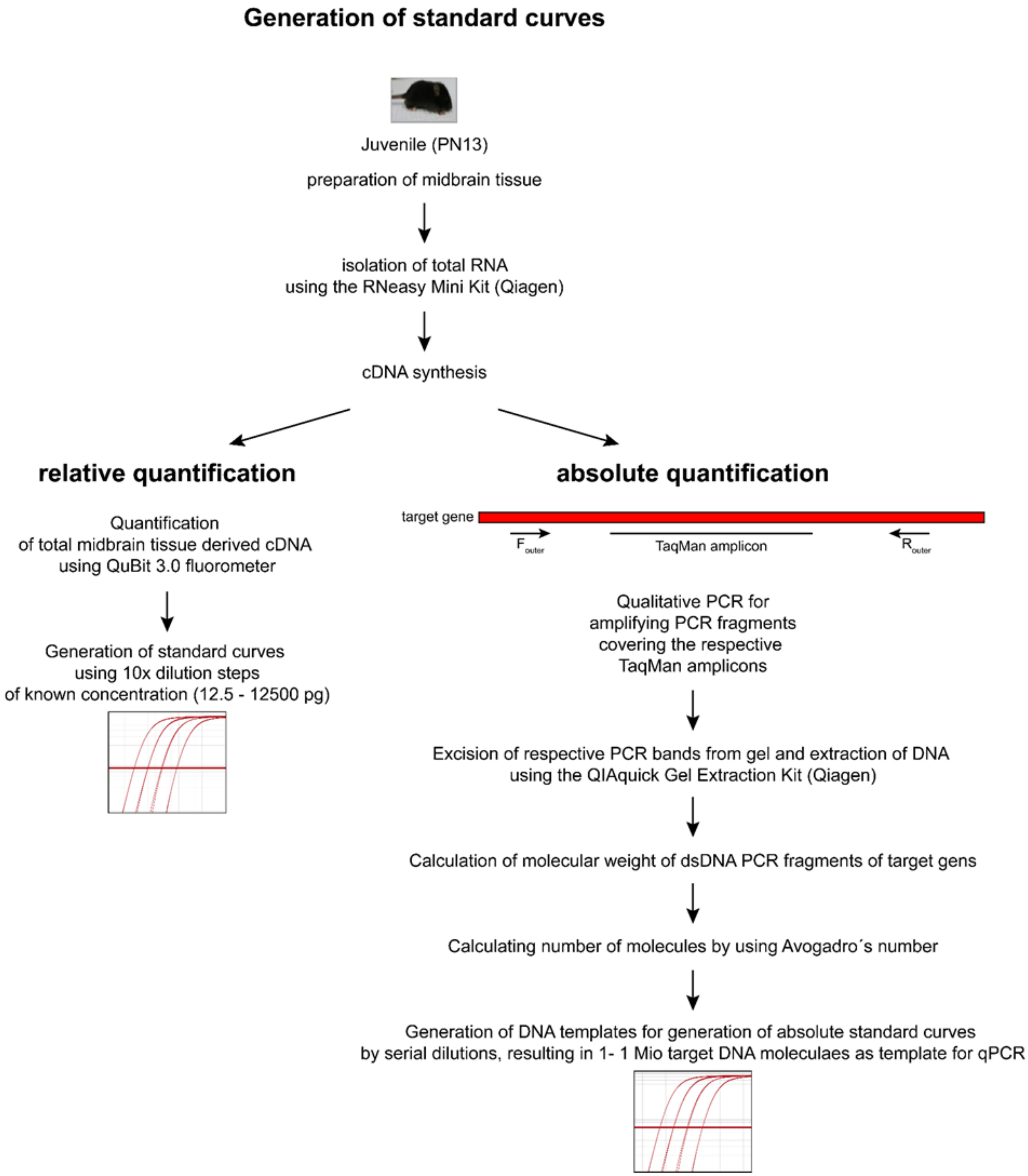


**Supplementary Figure 9.**

**General workflow of UV-LMD Workflow of standard curve generation for relative and absolute quantification of cDNA via qPCR.** For generation of standard curves, total RNA was isolated from

murine midbrain tissue using the RNeasy Mini Kit and reverse-transcribed into cDNA and purified, according to [5,6]. For generation of relative standard curves, cDNA concentration was determined using a QuBit 3.0. cDNA samples for generation of relative standard curves were generated by serial dilutions of this cDNA, containing 12.5 to 12,500 pg cDNA as templates. Respective dilution-ranges used for relative qPCR quantifications were determined for each analyzed gene while establishing the respective TaqMan assays. These samples for generation of relative standard curves were run in parallel with UV-LMD samples.

For generation of standard curves allowing absolute quantification of cDNA molecule numbers, a PCR fragment for each individual gene was amplified from mouse midbrain tissue derived cDNA, covering the respective location of the TaqMan assay. These PCR products were separated and purified by gel electrophoresis, band-excision and DNA-extraction, using the QIAquick Gel Extraction Kit. Purified PCR products were quantified using a Qubit 3.0. cDNA molecule numbers were calculated by calculating the molecular weights of dsDNA fragments and Avogadro's number, according to the formula: *Number of molecules = c x* $(N_A/MW)$,

*with c: concentration,* $N_A$*: Avogadro constant* $N_A = 6.02214086 \times 10^{23}\ mol^{-1}$*, MW: molecular weight of dsDNA fragment*

dsDNA was diluted according to the desired number of DNA molecules as templates in the respective standard curve qPCRs that were run in parallel with the UV-LMD samples[7]. Graphics created by Julia Benkert.

## Supplementary Methods

### Animals and chronic MPTP treatment

Juvenile (~PN13), adult (~PN90) and aged (~PN550) male C57BL/6J, NCS-1 knockout and NCS-1 wildtype mice[8] were bred at Ulm University. Cav2.3 knockout and Cav2.3 wildtype mice[9] (obtained from Toni Schneider) were bred at Ulm University as well as at the University of Cologne. The NCS-1 knockout (obtained from Olaf Pongs) is back-crossed at least 10 times into C57BL/6J[8], leading to a 99.9% analogy with C57BL/6J[10] and losing the 129/SvJ original background. Cav2.3 knockout is back-crossed only 4 times into C57BL/6J as they do not breed well, likely due to the presence of Cav2.3 channels in sperm[11], leading to a 75% C57BL/6J and 25% 129/SvJ mixed background. Data derived from littermate NCS-1 wildtype and Cav2.3 wildtype were not significantly different from those of C57BL/6J, wildtype data were pooled for some experiments. To obtain sufficiently large cohorts with animals of the same age for the MPTP experiments, animals from litters bred in parallel under identical housing conditions were pooled. Two independent breeding cohorts each were analyzed with this strategy. The MPTP induced degeneration of about 50% of SN dopaminergic neurons in the wildtype animals of this study is similar to that induced in other wildtype strains or in C57BL/6J mice[12-15]. To probe for differences in MPTP sensitivities due to different genetic backgrounds, we compared effects on wildtype strains from this study and other studies[12]. We robustly detect similar MPTP sensitivities in different wildtype strains and of different cohorts.

MPTP injections and perfusions were carried out at the University of Göttingen. Numbers of mice were calculated in accordance with biometric sample size estimation to detect a biologically relevant difference between compared groups. These calculations are a legal requirement to obtain approval from our federal ethical review committee (Regierungspräsidium Tübingen), to analyze a sufficiently large cohort of animals, but to prevent use of more animals than necessary (3R principle directive[16]). All animal procedures were approved by the German Regierungspräsidium Tübingen (Ref: 35/9185.81-3; TV-No. 921, 1043 and 1291, Reg. Nr. o.147) or the German Landesamt für Natur, Umwelt und Verbraucherschutz Nordrhein-Westfalen (Ref: 84-02.04.2016.A505) and carried out in accordance with the approved guidelines. Mice were injected 10 times with MPTP hydrochloride (20 mg/kg saline, subcutaneously, Sigma) and probenecid (250 mg/kg PBS buffer, intraperitoneally, Thermo Fisher) for 5 weeks every 3.5 days[17], according to the respective safety protocols and guidelines[18]. Control mice were injected with saline and probenecid only. One week after the last injections, mice were sacrificed and PFA-perfused for immunohistochemistry[12].

The chronic low-dose MPTP/probenecid model was chosen as it best simulates the progressive degeneration found in the human Parkinson's disease, and thus is generally accepted as the gold standard for preclinical testing of neuroprotective strategies for Parkinson's disease in rodents and monkeys[18-22]. MPTP induces selective loss of particularly SN dopaminergic neurons and Parkinson symptoms in animals and humans. Probenecid retards renal excretion and thus reduces biological variations[17]. Advantages of this chronic MPTP neurotoxin model, compared to the 6-OHDA neurotoxin Parkinson's disease-model are that MPTP is less invasive and better mimics the progression and pathology of Parkinson's disease[19,23]. 6-OHDA (in contrast to MPTP) cannot cross the blood-brain-barrier, which necessitates its direct injection into the SN or the striatum,

and consequently there is a larger variability of its outcome in dependence e.g. on the exact injection sites and 6-OHDA dose. This makes it more difficult to standardize conditions between laboratories[3,24]. Furthermore, the 6-OHDA model is less chronic (thus less chance to activate neuroprotective responses due to the Parkinson's disease-stressor), as neuronal death occurs over a brief time course (similar as in the acute MPTP models), and Lewy-bodies (as present in Parkinson's disease and the chronic MPTP model), are normally not found[19-23,25].

### Human samples

Informed consent was obtained from all Parkinson's disease-patients and control-subjects involved in this study before cell donation. The Hampstead Research Ethical committee previously approved the consent forms. All available information on the human donors is summarized in Supplementary Table 9A.

### *In vivo* retrograde tracing

For retrograde labeling of nigrostriatal SN dopaminergic and mesolimbic VTA dopaminergic neurons of adult mice[26], red fluorescent latex retrobeads (Lumafluor) were injected unilaterally into the dorsal striatum (DS) (coordinates: Bregma (y-axis) 0.98 & -0.1 mm, lateral (x-axis) 1.9 & 2.70 mm, ventral (z-axis) -3.2 mm; 2x 60 nl) or bilaterally into the ventral striatum (VS, Nucleus accumbens core / medial shell) (coordinates: Bregma (y-axis) 1.6 mm, lateral (x-axis) +/-0.8 mm, ventral (z-axis) -4.0 mm, 2x 6 nl) of anesthetized mice. Retrobead injection was performed under stereotactic control (Kopf Instruments) with a NanoFil syringe attached to a micropump (UMP3 with SYS-Micro4 Controller; World Precision Instruments) at a rate of 50 nl/min for DS and 30 nl/min for VS. Animals were sacrificed 7 days (DS) or 14 days (VS) after retrobead injection for $Ca^{2+}$ imaging or UV-laser-microdissection and reverse transcription quantitative PCR analysis. Injection sites were verified according to the mouse brain atlas[26,27].

### Combined $Ca^{2+}$ Imaging and electrophysiology

Electrophysiological recordings were combined with $Ca^{2+}$ imaging[3,28]. SN dopaminergic neurons were identified according to their sag component / slow Ih-current, broad action potentials, and post hoc by TH-immunohistochemistry, mesolimbic VTA dopaminergic neurons (from C57BL/6J mice) were identified by retrograde tracing and/or electrophysiological fingerprints, and post hoc by TH-immunohistochemistry[3,29,30]. Biocytin-streptavidin labeling was combined with TH-immuno-histochemistry[28]. Only electrotypical SN dopaminergic neurons and Nucleus Accumbens core / medial shell VTA dopaminergic neurons[29] were analyzed in this study.

Animals were anesthetized with isoflurane (B506; AbbVie Deutschland GmbH and Co KG, Ludwigshafen, Germany) and subsequently decapitated. The brain was rapidly removed and a block of tissue containing the mesencephalon was immediately dissected. Coronal slices (250 - 300 µm) containing the SN were cut with a vibration microtome (HM-650 V; Thermo Scientific, Walldorf, Germany) under cold (4°C), carbogenated (95% $O_2$ and 5% $CO_2$), glycerol-based modified artificial cerebrospinal fluid (GACSF). GACSF contained (in mM): 250 Glycerol, 2.5 KCl, 2 $MgCl_2$, 2 $CaCl_2$, 1.2 $NaH_2PO_4$, 10 HEPES, 21 $NaHCO_3$, 5 glucose adjusted to pH 7.2 (with NaOH) resulting in an osmolarity of ~310 mOsm. Brain slices were transferred into carbogenated artificial cerebrospinal fluid (ACSF). First, they were kept for 20 min in a 35°C 'recovery bath' and

then stored at room temperature (24°C) for at least 30 min prior to recording. ACSF contained (in mM): 125 NaCl, 2.5 KCl, 2 $MgCl_2$, 2 $CaCl_2$, 1.2 $NaH_2PO_4$, 21 $NaHCO_3$, 10 HEPES, and 5 Glucose adjusted to pH 7.2 (with NaOH) resulting in an osmolarity of ~310 mOsm. For the experiments, brain slices were transferred to a recording chamber (~1.5 ml volume) and continuously superfused with carbogenated ACSF at a flow rate of ~2 $ml \cdot min^{-1}$. Experiments were carried out at ~32°C. Recordings and electroporation were performed with an EPC10 amplifier (HEKA, Lambrecht, Germany) and a modified ELC03-XS amplifier with improved capacity compensation (NPI Electronic, Tamm, Germany) controlled by the software PatchMaster (version 2.32; HEKA). In parallel data were sampled at 10 kHz with a CED 1401 using Spike2 (version 7) (both Cambridge Electronic Design, UK) and low-pass filtered at 2 kHz with a four-pole Bessel filter**.** The liquid junction potential between intracellular and extracellular solution was compensated (14.6 mV; calculated with Patcher's Power Tools plug-in for Igor Pro 6 (Wavemetrics, Portland, OR, USA)).

Perforated patch recordings were performed using protocols modified from Horn & Marty[31] and Akaike & Harata[32]. Electrodes with tip resistances between 3 and 5 MOhm were fashioned from borosilicate glass (0.86 mm inner diameter; 1.5 mm outer diameter; GB150- 8P; Science Products) with a vertical pipette puller (PP-830; Narishige, London, UK). Patch recordings were performed with ATP and GTP free pipette solution containing (in mM): 128 Kgluconate, 10 KCl, 10 HEPES, 0.1 EGTA, 2 $MgCl_2$ and adjusted to pH 7.3 (with KOH) resulting in an osmolarity of ~300 mOsm. ATP and GTP were omitted from the intracellular solution to prevent uncontrolled permeabilization of the cell membrane[33]. The patch pipette was tip filled with internal solution and back filled with 0.02% tetraethylrhodamine-dextran (D3308, Invitrogen, Eugene, OR, USA) and amphotericin-containing internal solution (~200-250 $\mu g \cdot ml^{-1}$; G4888; Sigma-Aldrich, Taufkirchen, Germany) to achieve perforated patch recordings. Amphotericin was dissolved in dimethyl sulfoxide (final concentration: 0.1 - 0.3%; DMSO; D8418, Sigma-Aldrich)[34]; and was added to the modified pipette solution shortly before use. The used DMSO concentration had no obvious effect on the investigated neurons. During the recordings access resistance (*R*a) was constantly monitored and experiments were started after $R_a$ and the action potential amplitude were stable (~15 – 20 min). Recordings with $R_a$s > 50MΩ were not considered for analysis of intrinsic electrophysiological parameters. In the analyzed recordings $R_a$s were comparable, did not change significantly over recording time, and were not significantly different between the distinct experimental groups. The mean $R_a$ of all recordings was 41.3 ± 2.8 MΩ (n = 48). For the analysis of the action-potential waveform between the three different experimental groups (n=34; Supplementary Figure 1) the mean $R_a$s for the different dopaminergic neuron groups were not significantly different (SN dopaminergic wildtype: 40.9 ± 2.6 MΩ, n = 15; SN dopaminergic Cav2.3 knockout: 41.1 ± 3.3 MΩ, n = 12; VTA dopaminergic wildtype: 41.6 ± 3.8 MΩ, n = 7; n.s. ANOVA). A change to the whole-cell configuration was indicated by a sudden change in *R*a and diffusion of tetraethylrhodamine-dextran into the neuron. Such experiments were rejected. To block GABAergic and glutamatergic synaptic input the ACSF contained $10^{-4}$ M picrotoxin (P1675; Sigma-Aldrich), 5 x $10^{-5}$ M D-AP5; A5282; Sigma-Aldrich), and $10^{-5}$ M CNQX (C127; Sigma-Aldrich).

The imaging setup consisted of an Imago/SensiCam CCD camera with a 640x480 chip (Till Photonics) and a Polychromator IV (Till Photonics) coupled via an optical fiber into the upright microscope. Fura-2 was excited at 340 nm, 360 nm or 380 nm (410 nm dichroic mirror; DCLP410, Chroma). Emitted fluorescence was detected through a 440 nm long-pass filter. Data were

acquired as 80x60 frames using 8x8 on-chip binning. Images were recorded in arbitrary units (AU) and stored and analyzed as 12-bit grayscale images. Before establishing the perforated-patch clamp recordings fura-2 was loaded into the neurons by electroporation (1 V with 1 ms pulse duration at 65 Hz for 10 – 15 s). The loading pipette contained intracellular saline and 3.6 mM fura-2 (pentapotassium salt, F1200, Life Technologies). Loading was monitored at 360 nm excitation to reach comparable loading states (129 ± 9 AU, n = 48).

To measure $Ca^{2+}$ dynamics, pairs of frames excited with 340 nm and 380 nm were taken at 25 Hz (pacemaker activity) or 10 Hz (current induced actionpotentials, APs). The mean AU value within a region of interest (ROI) from the soma was determined. The ROI was adjusted for each cell. For background subtraction for the whole-time series an adjacent, second ROI was chosen. Data were analyzed as normalized fura-2 F340/F380 ratios. Mean amplitudes of 20 oscillations were calculated for each analyzed neuron. $Ca^{2+}$ imaging experiments were designed to assess if the actionpotential (AP) induced fluctuations of free intracellular $Ca^{2+}$ (referred to as $Ca^{2+}$ oscillations) are reduced in SN dopaminergic neurons from Cav2.3 knockout compared to Cav2.3 wildtype mice due to SNX-482. To achieve this, the relevant experimental conditions (e.g. indicator loading, AP-frequency) were carefully controlled to ensure equal conditions in all analyzed neurons. Since AP-associated $Ca^{2+}$ dynamics are strongly frequency dependent, the AP frequency was adjusted for $Ca^{2+}$ imaging in all recorded neurons to a similar value of ~1.5 Hz[3]. The distributions of spontaneous frequencies between the experimental groups were similar for a given cell type and accordingly the mean currents that were necessary to adjust the frequency to ~1.5 Hz were not significantly different (Supplementary Table 6B/D).

For pharmacology, 100 nM SNX-482 (Tocris) was bath applied (in ACSF) for at least 15 min before and during recordings. As SNX-482 besides Cav2.3 also inhibits voltage-sensitive A-type Kv4.3 $K^+$ channels with high nanomolar affinity[35], and these channels are prominently expressed in SN dopaminergic neurons and crucial for pacemaker frequency[7,36], SNX-482 $Ca^{2+}$ imaging experiments were performed in the presence of the A-type $K^+$ channel blocker 4-aminopyridine (4 mM, Sigma) for full channel inhibition. Since SNX-482 may also inhibit other voltage-gated $Ca^{2+}$ channels at higher concentrations[37], we have used a low concentration of 100 nM that should only inhibit Cav2.3 - but that does not fully inhibit Cav2.3 channels. For pharmacological experiments, neurons were hyperpolarized to ~ -70 mV and depolarizing currents (50 - 70 ms, 50 - 80 pA) were injected to induce two action potentials.

**Whole cell voltage-clamp recordings**

Whole cell voltage-clamp recordings were performed in vital mouse brain slices[2,4] Chemicals if not stated otherwise were obtained from Sigma Aldrich. Mice (PN11-13) were anaesthetized with isoflurane and decapitated. Brains were removed and coronal brain slices were prepared in ice-cold ACSF using a Vibroslice™ (Campden Instruments). ACSF contained in mM: 125 NaCl, 25 NaHCO3, 2.5 KCl, 1.25 NaH2PO4, 2 CaCl2, 2 MgCl2 and 25 glucose, and was gassed with Carbogen (95% O2, 5% CO2, pH7.4, osmolarity was 300 -310 mOsm/Kg.). Slices were allowed to recover for 30 min at room temperature (22-25°C) before use for electrophysiology.

For recordings, brain-slices were transferred into the recording chamber of an Axioscope 2 FS plus (Zeiss, Germany) electrophysiological set-up equipped with CF 8/4 camera (Kappa opto-

electronics GmbH, Germany). Recordings were carried out in a modified ACSF solution containing in mM: 125 NaCl, 25 NaHCO3, 2.5 KCl, 1.25 NaH2PO4, 2.058 MgCl2, 1.8 CaCl2, 2.5 glucose, 5 CsCl, 15 Tetraethylammonium, 2.5 4-Aminopyridine, 600 nM TTX (Tocris), 20 µM CNQX (Tocris), 4 µM SR 95531 (Tocris) and 10 µM DL-AP5 (Tocris), pH adjusted to 7.4, osmolarity was 300 -315 mOsm/Kg.
For electrophysiological data acquisition, an EPC10plus patch-clamp amplifier with PatchMaster software (v2x73, HEKA Elektronik) was used. Data were digitalised with 2 kHz, and filtered with Bessel Filter 1: 10 kHz; Bessel Filter 2: 5 kHz. All recordings were performed at a bath temperature of 35°C ± 1. Temperature was controlled using the Badcontroler V (Luigs & Neumann). Patch pipettes were pulled from borosilicate glass (GC150TF-15, Warner Instruments) using a DMZ Universal Puller (Zeitz-Instrumente GmbH). Patch pipettes (2.5-3.5 MΩ,) were filled with internal solution containing in mM: 180 N-Methyl-D-glucamine, 40 HEPES, 0.1 EGTA, 4 MgCl2, 5 Na-ATP, 1 Lithium-GTP, 0.1% neurobiotine tracer (Vector Laboratories); pH was adjusted to 7.35 with H2SO4, osmolarity was 285 – 295 mOsm/Kg. Neurons were filled with neurobiotin during the recording, fixed with a 4% PFA solution and stained for TH (rabbit anti-TH, 1:1000, Cat#: 657012, Merck Millipore) and neurobiotin (Streptavidin Alexa Fluor conjugate 647, 1:1000, Cat# S21374, Thermo Fisher Scientific). Only TH positive cells were used for the statistical analysis.

Cav-currents were activated by a voltage step protocol from a holding potential of -70 mV (10 mV steps, for 150 ms, 7 s interpulse-interval, see Supplementary Figure 3a). Current were leak subtracted on-line using the P/4 subtraction. The series resistance was compensated on 60-90%. Liquid junction potentials were not compensated. After control recordings, SNX-482 (100 nM) was washed in for 10 minutes prior to recordings. Two recordings each were averaged for further analysis.

Recordings from voltage step protocols were fitted with a modified Boltzmann function (formula 1), in GraphPad

(1) $I = G_{max}(V-V_{rev})/(1+\exp((V_{0.5}-V)/k))$,

where I is the peak current amplitude, $G_{max}$ is the maximal conductance [µS], V is the respective test potential [mV], $V_{rev}$ the reversal potential (135 mV calculated via Nernst equation), $V_{0.5}$ is the half-maximal activation voltage [mV], and k is the slope factor. Each individual cell was fitted, and also mean values for all cells were fitted.

Conductance was fitted by a Boltzmann sigmoidal function (formula 2) in GraphPad Prism.

(2) $G = G_{max}(1+\exp((V_{0.5}-V)/k))$,

where $G_{max}$ is the maximal conductance [nS], $V_{0.5}$ is the half-maximal activation voltage [mV], and k is the slope factor.

Area under the curves (AUC) were calculated using the integral function in GraphPad Prism.

**RNAScope® *in situ* hybridization**

In situ hybridization experiments were performed on fresh frozen mouse brain tissue using the RNAScope® technology (Advanced Cell Diagnostics, ACD), according to the manufacturer's protocol under RNase-free conditions. Briefly, 12 µm coronal cryosections were prepared[5], mounted on SuperFrost® Plus glass slides and dried for one hour at -20°C. Directly before starting the RNAScope procedure, sections were fixed with 4% PFA for 15 min at 4°C and dehydrated using an increasing ethanol series (50%, 75%, 100%, 100%), for 5 min each. After treatment with protease IV (ACD, Cat# 322340) for 30 min at room temperature, sections were hybridized with the respective target probes for 2 h at 40°C in a HybEZ II hybridization oven (ACD). Target probe signals were amplified using the RNAScope Fluorescent Multiplex Detection Kit (ACD, Cat# 320851). All amplifier solutions were drop on respective sections, incubated at 40°C in the HybEZ hybridization oven and washed twice with wash buffer (ACD) between each amplification step, for 2 min each. Nuclei were counterstained with DAPI ready-to-use solution (ACD, included in Kit) and slides were coverslipped with HardSet mounting medium (VectaShield, Cat# H-1400) and dried overnight.

Target probes were obtained from the library of validated probes provided by Advanced Cell Diagnostics (ACD). Details of all used target probes (RNAScope assays) used for analysis are provided in Supplementary Table 1A. The here used target probes detected but did not discriminate between all known splice variants of the respective target genes, i.e. voltage-gated $Ca^{2+}$ channel $\alpha_1$ subunits. Target genes, visualized with Atto550 fluorophore, were co-stained with Tyrosine hydroxylase (TH), and visualized with AlexaFluor488, as a marker for dopaminergic neurons. The gene peptidyl-prolyl isomerase B (PPIB) was used as positive control.

Fluorescent images of midbrain sections were acquired by a Leica CTR6 LED microscope using a Leica DFC365FX camera as z-stacks, covering the full depth of cells at 63x magnification. Z-stacks were reduced to maximum intensity Z-projections using Fiji (http://imagej.net/Fiji) and images were analyzed by utilizing a custom-designed algorithm (Wolution, Munich, Germany). The algorithm delineates cell shapes according to the TH marker gene signal and quantifies the area of fluorescent staining. According to Advanced Cell Diagnostics (ACD), target probe hybridization results in a small fluorescent dot for each mRNA molecule, allowing quantification of absolute number of mRNA molecules independent from fluorescent signal intensity.

**Cryosectioning, laser-microdissection, reverse transcription**

Coronal 12 µm cryosections were cut and mounted on PEN-membrane slides (Mirodissect)[2,38]. UV-laser-microdissection (UV-LMD) of SN dopaminergic neurons from cresyl-violet-stained midbrain sections from juvenile mice, or from retrogradely traced adult mouse midbrain sections, was carried out using an LMD7000 system (Leica Microsystems). UV-LMD, cell lysis, cDNA synthesis, precipitation, multiplex-nested PCR (for marker-gene analysis of murine samples) and quantitative real-time PCR (qPCR) of UV-LMD samples were performed[2,5,6,38]. Work-flow of the whole procedures is summarized in Supplementary Figure 1 and 2.

Reverse transcription was carried out in a volume of 5 µl, with 0.5% NP-40 (Roche), 5 U SuperRNasin (Thermo Fisher Scientific), 0.5 mM dNTPs (GE Healthcare Life Sciences), 5 µM

random hexamer primers (Roche Applied Science), 500 ng poly-inosine (Sigma), 10 mM DTT (Thermo Fisher Scientific), and 60 U of SuperScriptII (Thermo Fisher Scientific), in 1x first-strand buffer (Thermo Fisher Scientific), supplied with the SuperScriptII, directly without a distinct RNA isolation step in a one-tube procedure (0.5 ml RNase-free thin-walled PCR reaction tube from Axygen), at 38°C for 2 h on a Thermomixer (Eppendorf) and at 39°C overnight in a ThermoStat (Eppendorf)[1,5,6].

**Multiplex-nested PCR and qPCR analysis**

A multiplex-nested primer approach was used only for qualitative PCRs. Qualitative multiplex-nested PCR and quantitative qPCRs were carried out[2-6], using a GeneAmp PCR System 9700 thermocycler (Thermo Fisher Scientific) and the following PCR conditions. For multiplex PCR (HotStarTaq Master Mix, HotStarTaq Polymerase, Qiagen, 125 U, 50 µl total volume): 15 min 95°C; 35 cycles: 30 s 94°C, 1 min 58°C, 3 min 72°C; 7 min, 72°C. For nested PCRs (REDTaq Ready Mix, Taq Polymerase, Merck, 62.5 U, 25 µl total volume): 2.5 min 94°C; 35 cycles: 30 s 94°C, 1 min 58°C, 1 min 72°C; 7 min 72°C. For real-time qPCR a 7900HT Fast Real-Time PCR System or QuantStudio3 System were used (both Thermo Fisher Scientific) with the following cycle-conditions (QuantiTect Probe PCR Master Mix, HotStarTaq polymerase, Qiagen, 20 µl total volume): 2 min 50°C; 15 min 95°C; 50 cycles: 15 s 94°C, 1 min 60°C.

Only samples expressing a correct qualitative marker gene profile, defined by qualitative multiplex-nested PCR, were further analyzed by qPCR. The correct qualitative marker gene profile was defined as positive for TH, and negative for calbindin d28k (CB), glial fibrillary acidic protein (GFAP), and glutamic acid decarboxylase ($GAD_{65/67}$).

Details of all primers, qPCR assays (TaqMan®), and standard curve parameters used for analysis are provided in Supplementary Table 2A & 3. For qPCR assays, we tested at least three different TaqMan assays (i.e. a probe, flanked by two primers), targeting the same gene of interest, for each gene we analyze. For each individual gene (in Supplementary Table 3) only the assays are given that resulted in the most optimal performance over four orders of magnitude of template molecules (ideal slope: -3.32), the most robust reproducibility, and the highest sensitivity. All qPCR amplicons are very small (between 57-73 bp) (Supplementary Table 3). For absolute quantification of Cav1.2, Cav1.3, Cav2.3, and NCS-1 cDNA molecule numbers (data given in Figure 1b, Figure 4d and Supplementary Table 4A/E), defined amounts of cDNA molecules were utilized to generate absolute real-time PCR standard curves[3,7], and further illustrated in cartoon (B), next page.

To obtain DNA for generation of absolute standard curves, cDNA fragments covering the respective TaqMan® assay locations (used for qPCR) were amplified by PCR (primer sequences are given in Supplementary Table 2B, REDTaq Ready Mix, Taq Polymerase, Merck, 125 U, 50 µl total volume): 3 min 94°C; 35 cycles: 30 s 94°C, 1 min 63°C, 1 min 72°C; 7 min 72°C) and after gel electrophoresis, PCR products were purified (QIAquick Gel Extraction Kit, Qiagen), and quantified (with a Qubit 3.0 fluorometer). DNA molecule numbers were calculated by determining the molecular weight of the individual dsDNA fragments, and by using Avogadro's number. DNA samples containing 1,000,000 molecules down to 1 molecule (in 10-fold diluted steps) were used to generate absolute cDNA standard curves (pipetted in parallel with the respective SN

dopaminergic neuron derived cDNA-samples). These curves were used to calculate absolute cDNA molecule numbers, as further illustrated in cartoon (B), next page.

Absolute quantification of cell-specific cDNA levels is a much more complex, difficult, and time-consuming approach[3,7]; however only with this approach, the comparison of expression levels between different genes and different ages is valid. Thus we applied it only when necessary to prove a hypothesis (i.e. Figure 1b and Figure 4d but not Figure 3d and Figure 4c). Note again that all qPCR amplicons - for relative and for absolute quantification - are between 57-73 bp only (Supplementary Table 3).

Relative qPCR quantification data are given as cDNA amount [pg/cell] in respect to midbrain-tissue cDNA standard curves. Data were calculated according to formula (3)[6]:

$$(3)\ cDNA\ amount\ per\ cell\ [\frac{pg}{cell}] = \frac{S^{[(Ct-Y_{intercept})/slope]}}{No_{cells} \cdot cDNA\ fraction}$$

With S = serial dilution factor of the standard curve (i.e. 10), $No_{cells}$ = number of SN DA neurons per sample (i.e. 10 here), cDNA fraction = fraction of the cDNA reaction used as template in the qPCR reaction (i.e. 5/17) and the Y-intercept and slope of the relative standards (see Supplementary Table 3). Relative Cav1.2, Cav1.3, Cav3.1, Cav2.3 and NCS-1 cDNA levels in knockout mice were normalized to 1 for all respective wildtype data for each age.

Absolute qPCR data are given as cDNA molecules per cell, derived from absolute cDNA standard curve values for Y-intercept and slope (see Supplementary Table 3). Data were calculated according to formula (4)[6] (with S, $No_{cells}$, and cDNA fraction, as defined above):

$$(4)\ cDNA\ molecules\ per\ cell = \frac{S^{[(Ct-Y_{intercept})/slope]}}{No_{cells} \cdot cDNA\ fraction}$$

**Histological analysis and immunostaining**

Briefly[12], after perfusion and post-fixation in 4% PFA in PBS (pH 7.4) at 4°C overnight, the brains were stored in 0.05% $NaN_3$ in PBS at 4°C. Before cutting, brains were incubated for 1 h in cutting solution (10% sucrose and 0.05% $NaN_3$ in PBS) at 4°C. 30 µm coronal midbrain sections for stereology and for immunofluorescence and 100 µm coronal striatal sections for striatal fiber densitometric analysis were cut with a vibratome (VT 1000S, Leica). Free-floating slices were washed three times in PBS for 10 min each with shaking (300 rpm, microplate shaker, VWR) and treated for 2 h with a blocking solution (10% normal goat serum, 0.2% BSA and 0.5% Triton X-100 in PBS). After washing the slices with PBS, the primary antibody was applied in solution (1% goat serum, 0.2% BSA and 0.5% Triton X-100 in PBS).

Rabbit anti-TH for immunohistochemistry was incubated overnight at room temperature, the other primary antibodies used for immunofluorescence were incubated at 4°C while shaking to better preserve antigen integrity (300 rpm, Microplate shaker, VWR). Slices were washed three times in 0.2% Triton X-100 in PBS for 10 min and incubated with the respective secondary antibody for 2 h (for immunohistochemistry) or for 3 h (for immunofluorescence) at room temperature while shaking (300 rpm, Microplate shaker, VWR) in the solution described above.

TH immunostaining for stereology and densitometry was visualized using a VECTASTAIN® ABC system based on Horseradish peroxidase (HRP) detection (cat#: PK-6100, Vector Laboratories)

and as a substrate 3,3'-Diaminobenzidine (DAB) (cat#: SK-4100, Vector Labs). The slices were mounted on glass slides, dehydrated, and fixed by VectaMount Permanent Mounting Medium (cat#: H-5000, Vector Labs) with a coverslip for stereological and densitometric analysis.

The following primary antibodies were used: for stereology and densitometry anti-TH (made in rabbit, 1:5000, Cat#: 657012, Merck Millipore) and for semi-quantitative immunofluorescence anti-TH (made in mouse, 1:1000, Cat#: MAB318, Merck Millipore), anti-Cav2.3 (made in rabbit, 1:3000, Cat#: 27225-1-AP, Proteintech) and anti-NCS-1 (made in rabbit, 1:250, Cat#: 10506-2-AP, Proteintech).

Specificity of NCS-1 antibodies (Proteintech 10506-2-AP and abcam ab129166, respectively used for immunofluorescence in mouse brain and for Western blot on iPSCs) was confirmed by Western blots on total mouse brain lysates, and for anti-NCS-1 (Proteintech 10506-2-AP used for immunofluorescence) also by immunofluorescence on sections from NCS-1 wildtype and NCS-1 knockout mice, respectively (Supplementary Figure 6). Brains were homogenized in 1x RIPA buffer (#9806, Cell Signalling) added with 1 mM PMSF (#8553, Cell Signalling) by an electric homogenizer (T10 basic, Ultra Turrax) until the tissue was completely homogenized. After centrifugation at 4°C and 21,9 g (Heraeus Multifuge X3 Centrifuge, Thermo Fisher Scientific) for 20 min, the supernatant was isolated. The pellet was extracted again and supernatant collected. For these Western blots we used 10 µg of total brain lysates and the NCS-1 antibodies were diluted 1:4000 and 1:500 (Proteintech 10506-2-AP and abcam ab129166, respectively). As a loading control we used anti beta-actin antibody (1:5000, as-15, abcam).

The specificity of the Cav2.3 antibody used for immunofluorescence (Proteintech 27225-1-AP) was tested by Western blots and by immunofluorescence on sections from Cav2.3 wildtype and Cav2.3 knockout mice (Supplementary Figure 6). For these Western blots we used 30 µg of total brain lysates pre-heated at 30°C before loading and the Cav2.3 antibody was diluted 1:4000. As a loading control we used anti beta-actin antibody (1:5000, as-15, abcam).

The specificity of the Cav2.3 antibody used for the Western blots on iPSCs was already previously proved by Western blots in brain microsomal membrane lysates from Cav2.3 wildtype and Cav2.3 knockout mice, and published[9,39,40]. Note that the Cav2.3 antibody used for iPSC derived Western blot data was generated against human Cav2.3 protein.

The following secondary antibodies were used: for stereology and densitometry biotinylated anti-rabbit (made in goat, 1:1000, Cat#: BA1000, Vector Laboratories) and for immunofluorescence Alexa Fluor 488 anti-rabbit (made in goat, 1:1000, Cat#: A-11034, Thermo Fisher Scientific) and Alexa Fluor 546 anti-mouse (made in goat, 1:1000, Cat#: A-11003, Thermo Fisher Scientific).

Immunofluorescent stained sections were mounted on glass slides and the coverslip was fixed with VectaShield Mounting hard-set medium with DAPI (Cat#: H-1500, Vector Laboratories) for confocal imaging.

For automated counting of neurons, the sections were incubated in xylene to remove the coverslips and rehydrated by immersion in ethanol series (100%, 90%, 70%, 50%) and $H_2O$ (5 min each). After drying for 5 min, the slides were incubated with Vector hematoxylin QS (2 min) for counterstaning of the nuclei (Vector Hematoxylin QS Kit, Cat#: H-3404, Vector Laboratories), after dehydration and xylene, coverslips fixed by Vectamount.

**Optical densitometry, stereological and automated counting**

Optical densitometry and stereological analysis were performed[12]. Regions of interest namely dorsal striatum (DS), ventral striatum (VS), SN and VTA were identified according to established anatomical landmarks as by K. Franklin and G. Paxinos mouse brain atlas[27]. Analysis of TH-signal in dorsal and ventral striatum is less quantitative than stereological analysis of the remaining TH-positive neuron bodies in SN and VTA, and allows only a semiquantitative - but compared to high resolution stereology very quick - analysis of potential effects.

Relative optical densities of striatal TH-immunoreactivity were determined using the digital imaging software (Fiji). Images were acquired with the LMD Software 8.1.0.6156 (Leica) at the LMD7000 microscope. Striatal TH signal was quantified in 18 DS and 10 VS serial sections, covering the whole caudo-rostral axis (Bregma -0.3 to 1.7 for DS and Bregma 0.6 to 1.7 for VS). Respective signals from cortices were subtracted as background.

Total numbers of TH-labeled neurons in SN and VTA were determined with high-resolution stereology[12], using an unbiased optical fractionator method (StereoInvestigator software; MBF Bioscience). 40 serial TH-stained sections were analyzed, covering the whole caudo-rostral axis (Bregma -3.8 to -2.7).

Estimated total number of TH-positive neurons (N) was calculated for each animal according to equation (5):

(5) $N = \sum Q^{-} \cdot \frac{t}{h \cdot asf \cdot ssf}$

with $\Sigma Q^{-}$ = number of counted neurons, t = mean section thickness, h = counting frame height (i.e. 11 µm), asf = area sampling fraction (i.e. 0.44), and ssf = section sampling fraction (i.e. 1 for SN and 2 for VTA). Sampling grid dimensions were 75 x 75 µm (x,y-axes) and counting frame size were 50 x 50 µm (x,y-axes). Reliability of the estimation was evaluated by the Gundersen coefficient (CE, m = 1) according to equation (6):

(6) $CE = \frac{\sqrt{s^2 + VAR_{SRS}}}{s^2}$

(7) $VAR_{SRS} = \frac{3(A - s^2) - 4B + C}{240}$ for m=1

with $A = \sum_{i=1}^{n} (Q_i^-)^2$; $B = \sum_{i=1}^{n-1} Q_i^- Q_{i+1}^-$; $C = \sum_{i=1}^{n-2} Q_i^- Q_{i+2}^-$

with $s^2$ = variance due to noise and $VAR_{SRS}$ = variance due to systematic random sampling, according to equation (7) for m = 1. CE values were all ≤0.05 for all analyzed animals. Caudo-rostral axes were generated by plotting the mean relative optical densities, or the mean absolute counted number of neurons for each analyzed section for each animal. The experimenter was blinded for stereological analysis.

For automated neuron counting, stained sections were digitized with a whole slide scanner (3D-Histech Pannoramic 250 Flash III, Sysmex Deutschland GmbH, Norderstedt, Germany). The digital images were acquired at 0.25 µm/pixel resolution using 9 focal layers with 1 µm intervals between layers. The layers were combined using extended focus mode. The digital slides were uploaded on Aiforia® Cloud platform (Fimmic Oy, Helsinki, Finland). The Aiforia platform was used for supervised training of a convolutional neural network (CNN) algorithms to detect all neurons or

only TH-positive neurons similarly as in Penttinen et al.[41]. The training was based on nuclear/cell morphology and TH signal. We used 5079 and 4064 neurons to train the CNNs to count all neurons and TH-positive neurons only, respectively. To validate the trained CNNs, we compared the absolute counts of TH-positive neurons per animal with the respective stereological estimates (see Supplementary Figure 5 and Supplementary Table 8A) in SN regions. SN and VTA regions were manually annotated directly on the digital images in Aiforia Cloud. In case of a flawed section the mean value of counted neurons from the section before and the section after was taken, to avoid methodological bias, as with automated counting all neuron were counted, compared to a fraction only and a calculated estimate by stereology.

**Semi-quantitative analysis by confocal immunofluorescence**

The quantitative analysis of NCS-1 and Cav2.3 antibody immunofluorescence signals was performed in mouse TH-positive SN and VTA neurons[36,42]. High-resolution images of midbrain sections, immunostained by either NCS-1 or Cav2.3 together with TH and DAPI (4',6-diamidino-2-phenylindole) were acquired by a Zeiss LSM 710 confocal microscope (Carl Zeiss, Germany) using a 63x oil objective. Settings were adjusted by the ZEN software (Carl Zeiss, Germany).

Fluorochromes were excited using an argon laser at 405 nm, 488 nm and 546 nm for DAPI, NCS-1 or Cav2.3 and TH respectively, avoiding signal saturation and keeping confocal settings identical for all analyzed neurons / animals. The LUT (Look Up Table) function provided by the Range Indicator in the Zeiss software was used to ensure that the fluorescence signal for quantification was not saturated. In particular, the Range Indicator allows to visualize saturating pixels, i.e. pixel values above a threshold of 200, in red. We ensured that the acquisition had no saturated pixels by reducing the laser power, detector gain and amplifier offset according to the method information related to the Zeiss LSM710 ZEN software. In this way we ensured to be within the dynamic range of the detector critical for semi-quantitative analysis (0-255 for 8-bit images and 0-65536 for 16-bit images).

For NCS-1 we used the following parameters: master gain = 655 for NCS-1, and 800 for TH, 750 for DAPI; digital Gain = 1; shutter speed = 4; pinhole diameter = 29.9, 0.6 AU; one directional acquisition with 1912 X 1912 frame size; bit depth = 12. For Cav2.3 we used: master gain = 800 for Cav2.3, and 800 for TH, 750 for DAPI; digital Gain = 1; shutter speed = 4; pinhole diameter = 29.9, 0.6 AU; one directional acquisition with 1912 X 1912 frame size; bit depth = 12. All the images were converted to 8-bit (0-255), and only TH-positive neurons showing a visible nucleus were used for the analysis. TH immunoreactivity was used to identify and mark TH-positive cytoplasmic regions of interest (ROIs). The DAPI staining was used to identify and mark the nucleus of TH-positive cells, used as background noise ROI. The NCS-1 signal intensities were measured in the cell body / cytoplasm ROI (note that some minor expression is described for (peri)nuclear regions). Cav2.3 signal intensities were measured in the plasma membrane ROI by using the Fiji software[43] (https://fiji.sc/). We determined average signal intensities / pixel of the antibody fluorescent signal in the plasma membranes or cell bodies of individual TH-positive SN and VTA neurons. These values are independent from the respective areas of the neurons. The individual signal intensities were normalized to the background signal in the respective cell nuclei for all analyzed neurons, and given relative to that of VTA DA neurons (=1). The high background signal for NCS-1 and its overlapping Gaussian curve with the signal can be explained by a possible

signal of NCS-1 in the perinuclear space as it has been shown in dopaminergic like PC12 neurons and HeLa cells[44] and also in cardiomyocytes[45]. For each mouse, seven coronal midbrain sections (30 µm after fixation, covering the full caudo-rostral axis) and about 20-25 TH-positive neurons per section (130-150 TH-positive DA neurons per animal) were analyzed for the SN as well as for the VTA respectively (N=4 mice).

**Differentiation of human iPSC and western blot analysis**

Induced pluripotent stem (iPS) cells were derived from dermal fibroblasts from human donors from the discovery clinical cohort established by the Oxford Parkinson´s disease Centre (OPDC) (see Supplementary Table 9A)[46] and differentiated into dopaminergic neuronal cultures according to a modified protocol[47,48]. Basal media are as follows (Life Technologies unless stated otherwise): KO DMEM KSR = Knockout DMEM, Knockout serum replacement, 1X non-essential amino acids, 2 mM L-glutamine, 10 µM 2-mercaptoethanol (Sigma); NNB = Neurobasal medium, 0.5X N2 supplement, 0.5X B27 supplement, 2mM L-glutamine; NB = Neurobasal medium, 1X B27 supplement, 2mM L-glutamine. Differentiation factors added to basal media are as follows: 100 nM LDN-193189 (Sigma), 10 µM SB-431542 (Tocris Bioscience), 100 ng/ml recombinant sonic hedgehog C24II (R&D Systems), 2 µM purmorphamine (Calbiochem), 100 ng/ml fibroblast growth factor 8a (R&D Systems), 3 µM CHIR-99021 (Tocris Bioscience), 20 ng/ml brain-derived neurotrophic factor (Peprotech), 20 ng/ml glial cell line-derived neurotrophic factor (Peprotech), 1 ng/ml transforming growth factor β3 (Peprotech), 10 µM DAPT (abcam), 200 µM ascorbic acid (Sigma), 500 µM dibutyryl cAMP (Sigma). Medium containing differentiation and neurotrophic factors was fully changed every two days with half change every other day until day 20. Cells were then dissociated with StemPro Accutase (Life Technologies) and re-plated onto Geltrex at 300,000 cells/cm$^2$ in 6 well plate or T25. Cultures were treated at day 22 with 1 µg/ml mitomycin C in NB medium for 1 hr to remove proliferating cells and washed with neurobasal medium before returning to fresh maturation medium. After a full medium change three days later to remove dead cells, medium was half changed every 2-3 days for the remaining period of maturation up to DIV35. All cell cultures were maintained at 37°C, 5% $CO_2$.

Cells were extracted with cold PBS and centrifuged at 500 x g for 5 min. Cell pellets were snap frozen and stored at -80°C until required. Frozen cell pellets were lysed in RIPA buffer on ice (TRIS 1M pH8, iGEPAL, Sodium Deoxycolate 10%, protease inhibitor cocktail (Sigma) 1 tablet in 10 ml). Cell lysates were briefly sonicated (10 sec) then centrifuged 10 min at 4°C at full speed. Supernatant protein concentration was quantified by BCA and diluted into appropriate concentration in Laemmli sample buffer (4X). Extracted samples were boiled for 5 min at 90°C, proteins were separated by SDS-polyacrylamide gel electrophoresis (precast gel 4-15%) with a homemade running buffer (144 g glycine; 30.3 g TRIS Base 10 g SDS for 1 L of 10X buffer) and transferred onto PVDF membrane using Trans-Blot Turbo Transfer System (Bio-Rad) (High molecular weight protocol, midi membrane). Blots were stained with Ponceau S for 1 min.

The western blot was blocked in 5% Milk in PBS-T (0.1%) for 1 h at room temperature before incubation with the primary antibody (rabbit anti-NCS-1, 1:500, Abcam ab129166; and rabbit anti-$Ca_V$2.3 common (anti-Nast 197B, self-designed, obtained from Toni Schneider)[49], 1:500; and mouse anti- beta actin HRP conjugated, 1:10000, Abcam) at 4°C, overnight. It was then washed 2 times for 10 min in PBS-Tween (0.05%) and once for 10 min in TBS-Tween (0.05%) before

incubation for one hr at room temperature with secondary antibody (goat anti-rabbit IgG (H+L)-HRP Conjugate, 1:20000, Biorad) diluted in 5% Milk in PBS-T (0.1%). Finally, the blot was washed again as before and imaged on the ChemiDoc-Touch (Bio-Rad) with ECL substrate (Millipore) 1:1. Densitometric analysis of each protein was performed and normalized to actin prior to the calculation of Cav2.3 / NCS-1 ratios. Due to differentiation variability, all 4 control lines (see Supplementary Table 9A) were included in every "differentiation batch/experiment" and normalized to 1 for all respective control data in each differentiation.

Immuno-staining for the established markers neuron-specific class III beta-tubulin (Tuj1, mouse anti-Tuj1 antibody, Biolegend, Cat# 801202, 1:500) and tyrosine hydroxylase (TH, rabbit anti-TH, Sigma, Cat# Ab152, 1:500) has been performed[46], and electrophysiological properties were assessed in order to attest to differentiation into dopaminergic neurons (Supplementary Figure 7). In our experimental design we chose to use multiple independent lines from different control individuals and Parkinson' disease patients to evaluate the effect of the GBA *N370S* mutation on dopaminergic neurons across six separate differentiations.

**Data analysis**

Data analysis and graphical illustrations were performed using FitMaster software (v2x90.2, HEKA Electronics), GraphPad Prism 7 (GraphPad Software, Inc.), Adobe Illustrator CC2015.3 (Adobe Systems Software), Igor Pro 6 (Wavemetrics Inc.), Spike2 (CED), Neuroexplorer (Nex Technologies), SDS 2.4 (Applied Biosystems), StereoInvestigator (MBF Bioscience), and Fiji (https://imagej.net/Fiji)[43] software.

For normalization of MPTP data, remaining TH-positive fibers or neurons respectively, in the MPTP treated groups were calculated as the percent of the corresponding saline treated group (particularly important for NCS-1 knockout, as knockout controls had lower numbers of SN dopaminergic neurons than wildtype (compare Figure 4a, and Supplementary Table 8C). In an alternative bootstrapping approach, each individual MPTP value was compared to each individual saline value of the respective groups for calculation of % of remaining TH signal intensity and neuron numbers (see Supplementary Figure 4, and Supplementary Table 8A-D).

Sample sizes for all mouse data were chosen in advised and in agreement with the Institute of Epidemiology and Medical Biometry, Ulm University, and in agreement with EU regulations (Principles of Replacement, Reduction and Refinement (3R's), Directive 2010/63/EU) and as approved by the German "Regierungspräsidium". No particular procedure was required for randomization/allocating wildtype and knockout mice to the respective experimental groups.

Data were tested for outliers via the ROUT function of GraphPad Prism 7. Tests for statistically significant differences were performed in GraphPad Prism 7 using statistical tests as indicated in the text. Parametric statistical tests were only chosen if data were normally distributed. Normal distribution was tested with D`Agostino-Pearson omnibus normality test. Unless otherwise stated, unpaired tests were used. Individual test are given in the Supplementary Tables 1-9. Statistical significances are indicated as $p < 0.05$ (*), $p < 0.01$ (**), $p < 0.001$ (***) and $p < 0.0001$ (****). In graphs, data are given as mean ± SD or SEM as indicated. In boxplots, horizontal lines indicate the median of the data, + sign indicates the mean of the data; boxes indicate the $25^{th}$ and $75^{th}$ percentile and whiskers were calculated according to the Tukey method. For experiments

with low n-numbers more conservative non-parametric tests were applied, even if data were normally distributed. For comparisons of multiple groups, non-parametric Kruskal Wallis test with Dunn's multiple comparisons test or two-way ANOVA with Sidak's multiple comparison test were performed as indicated.

**Data availability statement**

Most data presented are included in the article and supplementary information. All datasets generated are available from the corresponding author on request.

**Supplementary Table 1A/B.**

**Single cell RNAScope assay information and data.** Data & statistics of genes as indicated for graphs shown in Figure 1a. n represents number of analyzed dopaminergic neurons derived from N individual mice. Significances according to Kruskal Wallis test with Dunn's multiple comparisons test (significant values in bold).

**A) Details of target probes from Advanced Cell Diagnostics (ACD) for RNAScope based mRNA quantification.**

| Gene | Cat No. (ACD) | Assay target region according to respective Genbank accession no. (NCBI) |
|---|---|---|
| Tyrosine hydroxylase | 317621 | 483 – 1603 of NM_009377.1 |
| Cav1.2 (*Cacna1c*) | 445451 | 4846 – 6605 of NM_009781.4 |
| Cav1.3 (*Cacna1d*) | 502591 | 522 – 1994 of NM_001302637.1 |
| Cav2.1 (*Cacna1a*) | 493141 | 5705 – 6727 of NM_007578.3 |
| Cav2.2 (*Cacna1b*) | 468811 | 1295 – 2850 of NM_001042528.2 |
| Cav2.3 (*Cacna1e*) | 449211 | 2319 – 3317 of NM_009782.3 |
| Cav3.1 (*Cacna1g*) | 459761 | 1263 – 2886 of NM_009783.3 |
| Cav3.2 (*Cacna1h*) | 459751 | 2287 – 4243 of NM_021415.4 |
| Cav3.3 (*Cacna1I*) | 459781 | 1259 – 2600 of M_001044308.2 |

| | **B) Number of mRNA molecules per SN DA neuron (adult wildtype)** | | | | | |
|---|---|---|---|---|---|---|
| analyzed mRNA | **mean** | **±SD** | **±SEM** | **median** | **95% CI** | **n/N** |
| **Cav1.2** | 22.22 | 12.26 | 0.47 | 20.53 | 19.67-21.60 | 667/4 |
| **Cav1.3** | 30.06 | 16.37 | 0.71 | 27.33 | 25.73-29.47 | 537/4 |
| **Cav2.1** | 65.40 | 30.19 | 1.47 | 61.40 | 57.13-64.53 | 425/3 |
| **Cav2.2** | 61.17 | 26.84 | 1.25 | 61.07 | 58.47-64.27 | 461/3 |
| **Cav2.3** | 82.97 | 40.86 | 1.96 | 76.67 | 72.00-80.93 | 435/4 |
| **Cav3.1** | 41.79 | 23.03 | 0.90 | 38.27 | 35.87-40.53 | 657/4 |
| **Cav3.2** | 6.40 | 5.37 | 0.22 | 4.87 | 4.33-5.47 | 573/4 |
| **Cav3.3** | 9.34 | 6.14 | 0.28 | 8.37 | 7.67-9.33 | 498/4 |
| **p-values** | **<0.0001**; Cav1.2 vs. Cav1.3: **<0.0001**, Cav1.2 vs. Cav2.1: **<0.0001**, Cav1.2 vs. Cav2.2: **<0.0001**, Cav1.2 vs. Cav2.3: **<0.0001**, Cav1.2 vs. Cav3.1: **<0.0001**, Cav1.2 vs. Cav3.2: **<0.0001**, Cav1.2 vs. Cav3.3: **<0.0001**, Cav1.3 vs. Cav2.1: **<0.0001**, Cav1.3 vs. Cav2.2: **<0.0001**, Cav1.3 vs. Cav2.3: **<0.0001**, Cav1.3 vs. Cav3.1: **<0.0001**, Cav1.3 vs. Cav3.2: **<0.0001**, Cav1.3 vs. Cav3.3: **<0.0001**, Cav2.1 vs. Cav2.2: >0.9999, Cav2.1 vs. Cav2.3: **0.0355**, Cav2.1 vs. Cav3.1: **<0.0001**, Cav2.1 vs. Cav3.2: **<0.0001**, Cav2.1 vs. Cav3.3: **<0.0001**, Cav2.2 vs. Cav2.3: **0.0007**, Cav2.2 vs. Cav3.1: **<0.0001**, Cav2.2 vs. Cav3.2: **<0.0001**, Cav2.2 vs. Cav3.3: **<0.0001**, Cav2.3 vs. Cav3.1: **<0.0001**, Cav2.3 vs. Cav3.2: **<0.0001**, Cav2.3 vs. Cav3.3: **<0.0001**, Cav3.1 vs. Cav3.2: **<0.0001**, Cav3.1 vs. Cav3.3: **<0.0001**, Cav3.2 vs. Cav3.3: **0.0252** | | | | | |

**Supplementary Table 2A/B.**
**A) Details of multiplex PCR (outer) and nested PCR (inner) primers for qualitative PCR.**

Work-flow of the procedures is summarized in the supplementary methods and Figures A & B within. F: forward primer, R: reverse primer.

| Gene | Primer | Sequence (5`-3`) | Genbank accession no. (NCBI) | 5`-Position | Amplicon size [bp] |
|---|---|---|---|---|---|
| Mouse Calbindin-d28k (CB) | F (outer) | CGCACTCTCAAACTAGCCG | M21531 | 87 | 891 |
| | R (outer) | CAGCCTACTTCTTTATAGCGCA | | 977 | |
| | F (inner) | GAGATCTGGCTTCATTTCGAC | | 167 | 440 |
| | R (inner) | AGTTCCAGCTTTCCGTCATTA | | 606 | |
| Mouse Glial fibrillary acidic protein (GFAP) | F (outer) | AGAACAACCTGGCTGCGTAT | K01347 | 407 | 786 |
| | R (outer) | GCTCCTGCTTCGAGTCCTTA | | 1192 | |
| | F (inner) | AGAAAGGTTGAATCGCTGGA | | 472 | 517 |
| | R (inner) | CCAGGGCTAGCTTAACGTTG | | 988 | |
| Mouse Glutamate decarboxylase (GAD65) | F (outer) | CATACGCAGACAGCACGTTT | NM_008078.1 | 166 | 905 |
| | R (outer) | AAAAGATTCCATCGCCAGAG | | 1070 | |
| | F (inner) | GGGATGTCAACTACGCGTTT | | 606 | 389 |
| | R (inner) | CACAAATACAGGGGCGATCT | | 994 | |
| Mouse Glutamate decarboxylase (GAD67) | F (outer) | TGACATCGACTGCCAATACC | Z49976 | 731 | 1105 |
| | R (outer) | GGGTTAGAGATGACCATCCG | | 1835 | |
| | F (inner) | CATATGAAATTGCACCCGTG | | 761 | 702 |
| | R (inner) | CGGTGTCATAGGAGACGTCA | | 1462 | |
| Mouse Tyrosine hydroxylase (TH) | F (outer) | CACCTGGAGTACTTTGTGCG | M69200 | 387 | 1139 |
| | R (outer) | CCTGTGGGTGGTACCCTATG | | 1525 | |
| | F (inner) | TGCACACAGTACATCCGTCA | | 936 | 377 |
| | R (inner) | TCTGACACGAAGTACACCGG | | 1312 | |

**B) Details of primers for qualitative PCR for amplifying DNA covering the respective TaqMan assays for generation of absolute standard curves (primers not used for quantitative PCR).**

Work-flow of the procedures is summarized in the supplementary methods and Figure B within.

| Gene | Primer | Sequence (5`-3`) | Genbank accession no. (NCBI) | 5`-Position | Amplicon size [bp] |
|---|---|---|---|---|---|
| mCav1.2 (*Cacna1c*) | F | TCACCACTCTGCTGCAGTTC | NM_001159533.1 | 3318 | 392 |
| | R | GACGAAACCCACGAAGATGT | | 3709 | |
| mCav1.3 (*Cacna1d*) | F | TCGGGACTGGTCTATTCTGG | NM_001083616 | 4863 | 480 |
| | R | TACTTCCCCACCAGTCCTTG | | 5342 | |
| mCav2.3 (*Cacna1e*) | F | TTGGATCTGCTTGTGCCCATG | NM_009782 | 5561 | 414 |
| | R | GGAATTTGAACGCTTATCCCGAA | | 5974 | |
| mNCS-1 (*Ncs1*) | F | CCGAGCATGGGGAAATCCAAC | NM_019681.3 | 251 | 472 |
| | R | TCATGGCAAAGATCCGGTCCAC | | 722 | |

**Supplementary Table 3.**

**Details of TaqMan qPCR assays and standard curve information, used for relative and absolute qPCR-based transcript quantification.**

Note that all amplicons are very small (between 57-73 bp). All probes are 5’-FAM (6-carboxyfluorescein) and 3’-NFQ (non-fluorescent quencher) labelled. P: probe, F: forward primer, R: reverse primer; m: mouse, bp: amplicon size; EB: exon-boundary spanned; T: threshold for analysis; Y: Y-intercept of standard curve. Y, slope and $R^2$ given as mean ± SEM.

| | | | | | | standard curve data | | | | |
|---|---|---|---|---|---|---|---|---|---|---|
| **Gene** | **Assay ID** | **TaqMan assay: primer/probe or consensus sequences** | **Gen-bank No** | **bp** | **EB** | **T** | **Y** | **slope** | **$R^2$** | **n** |
| mCav1.2 (*Cacna1c*) | Mm01188822_m1 | TCGAAGGGTGGCCAGAGCTGCTGTA | NM_001159533.1 | 65 | 26-27 | Absolute quantification: | | | | |
| | | | | | | 0.6 | 39.95 ± 0.06 | -3.35 ± 0.02 | 0.99 ± 0.00 | 4 |
| | | | | | | Relative Quantification: | | | | |
| | | | | | | 0.07 | 42.21 ± 0.24 | -3.52 ± 0.06 | 0.99 ± 0.00 | 6 |
| mCav1.3 (*Cacna1d*) | Mm00551392_m1 | TCCCTCCAGCTGGTGATGATGAGGT | NM_001083616 | 62 | 38-39 | Absolute quantification: | | | | |
| | | | | | | 0.6 | 41.12 ± 0.08 | -3.44 ± 0.02 | 0.99 ± 0.00 | 3 |
| | | | | | | Relative Quantification: | | | | |
| | | | | | | 0.06 | 43.92 ± 0.28 | -3.51 ± 0.08 | 1.00 ± 0.00 | 4 |
| mCav3.1 (*Cacna1g*) | Mm00486549_m1 | GTACCACGAGCAGCCCGAGGAGCTC | NM_009783 | 57 | 9-10 | 0.05 | 40.56 ± 0.09 | -3.34 ± 0.04 | 0.99 ± 0.00 | 4 |
| mCav2.3 (*Cacna1e*) | Mm00494444_m1 | TTCTGGCCTGAGTGGTCGGAGTGGA | NM_009782 | 63 | 43-44 | 0.5 | Absolute quantification: | | | |
| | | | | | | | 39.15 ± 0.13 | -3.37 ± 0.01 | 1.00 ± 0.00 | 3 |
| | | | | | | | Relative Quantification: | | | |
| | | | | | | | 43.32 ± 0.19 | -3.41 ± 0.02 | 0.99 ± 0.00 | 10 |
| mNCS-1 (*Ncs1*) | Mm00490549_m1 | CGAGAACAAGGATGGCAGGATTGAG | NM_019681.3 | 73 | 3-4 | Absolute quantification: | | | | |
| | | | | | | 0.3 | 39.26 ± 0.38 | -3.35 ± 0.05 | 1.00 ± 0.00 | 3 |
| | | | | | | Relative quantification: | | | | |
| | | | | | | 0.07 | 40.89 ± 0.37 | -3.49 ± 0.07 | 1.00 ± 0.00 | 4 |

**Supplementary Table 4A/B/C/D.**

**Single cell qPCR data of mouse SN dopaminergic neurons.** RT-qPCR data & statistics of genes as indicated for graphs shown in Figure 1b (A), Figure 3d (B, C), Figure 4c (D) and Figure 4d (E) given as mean, ±SD, ±SEM, median and 95% confidence interval (CI). n represents number of analyzed SN dopaminergic samples. Significances according to two-way ANOVA with Sidak's multiple comparison test (A) and MWU-test (B-E) (significant values in bold). #data adapted from (PMID:28592699).

| | **A) juvenile vs. adult wildtype mice** | | | | | | | | | | | |
|---|---|---|---|---|---|---|---|---|---|---|---|---|
| | **Juvenile** | | | | | | **adult** | | | | | |
| analyzed mRNA | **mean** | **±SD** | **±SEM** | **median** | **95% CI** | **n** | **mean** | **±SD** | **±SEM** | **median** | **95% CI** | **n** |
| **mCav2.3** | 10.3 | 3.3 | 0.9 | 10.7 | 7.3-13.5 | 13 | 21.4 | 13.9 | 2.7 | 15.6 | 10.7-29.1 | 27 |
| **mCav1.2#** | 8.4 | 3.4 | 0.7 | 7.6 | 6-1-10.4 | 26 | 6.1 | 3.4 | 0.7 | 5.9 | 3.7-8.0 | 25 |
| **mCav1.3#** | 6.5 | 3.0 | 0.6 | 6.3 | 4.3-8.0 | 24 | 3.9 | 2.3 | 0.5 | 3.4 | 2.3-5.3 | 24 |
| **p-value** | Cav1.2 vs Cav1.3: >0.9999; Cav1.2 vs Cav2.3: 0.9996; Cav1.3 vs Cav2.3: 0.9322 | | | | | | Cav1.2 vs Cav1.3: 0.9989; Cav1.2 vs Cav2.3: **<0.0001**; Cav1.3 vs Cav2.3: **<0.0001** | | | | | |

two-way ANOVA: Cav1.2 vs. Cav1.3 vs. Cav2.3: p=0.0959, juv. vs. adult: **p<0.0001**, interaction: **p<0.0001**.

| | **B) mRNA in adult Cav2.3 wildtype vs. Cav2.3 knockout mice** | | | | | | | | | | | | |
|---|---|---|---|---|---|---|---|---|---|---|---|---|---|
| | **Cav2.3 WT** | | | | | | **Cav2.3 KO** | | | | | | |
| analyzed mRNA | **mean** | **±SD** | **±SEM** | **median** | **95% CI** | **n** | **mean** | **±SD** | **±SEM** | **median** | **95% CI** | **n** | **p-value** |
| **Cav1.2** | 1.0 | 0.4 | 0.1 | 0.9 | 0.6-1.5 | 7 | 1.1 | 0.5 | 0.2 | 1.0 | 0.4-1.9 | 7 | >0.9999 |
| **Cav1.3** | 1.0 | 0.3 | 0.1 | 0.9 | 0.8-1.1 | 16 | 1.3 | 0.6 | 0.2 | 1.1 | 0.9-1.5 | 17 | 0.1704 |
| **Cav3.1** | 1.0 | 0.4 | 0.1 | 1.0 | 0.6-1.5 | 10 | 0.9 | 0.5 | 0.2 | 1.0 | 0.2-1.5 | 10 | 0.8534 |

| | **C) NCS-1 mRNA in Cav2.3 wildtype vs. Cav2.3 knockout mice** | | | | | | | | | | | | |
|---|---|---|---|---|---|---|---|---|---|---|---|---|---|
| | **Cav2.3 WT** | | | | | | **Cav2.3 KO** | | | | | | |
| | **mean** | **±SD** | **±SEM** | **median** | **95% CI** | **n** | **mean** | **±SD** | **±SEM** | **median** | **95% CI** | **n** | **p-value** |
| **Juvenile (PN 13)** | 1.0 | 0.4 | 0.1 | 0.9 | 0.6-1.4 | 10 | 1.1 | 0.3 | 0.1 | 1.0 | 0.8-1.5 | 10 | 0.4359 |
| **Adult (PN 90)** | 1.0 | 0.3 | 0.1 | 1.0 | 0.7-1.2 | 10 | 1.4 | 0.4 | 0.1 | 1.4 | 1.1-1.7 | 10 | **0.0288** |

| | **D) Cav2.3 mRNA in NCS-1 wildtype vs. NCS-1 knockout mice** | | | | | | | | | | | | |
|---|---|---|---|---|---|---|---|---|---|---|---|---|---|
| | **NCS-1 WT** | | | | | | **NCS-1 KO** | | | | | | |
| | **mean** | **±SD** | **±SEM** | **median** | **95% CI** | **n** | **mean** | **±SD** | **±SEM** | **median** | **95% CI** | **n** | **p-value** |
| **Juvenile (PN 13)** | 1.0 | 0.3 | 0.1 | 1.0 | 0.6-1.4 | 14 | 0.5 | 0.3 | 0.1 | 0.5 | 0.3-0.7 | 20 | **0.0002** |
| **Aged (PN 550)** | 1.0 | 0.4 | 0.1 | 1.0 | 0.7-1.3 | 15 | 0.6 | 0.2 | 0.1 | 0.6 | 0.4-0.7 | 14 | **0.0007** |

| | **E) juvenile vs. adult wildtype mice** | | | | | | | | | | | | |
|---|---|---|---|---|---|---|---|---|---|---|---|---|---|
| | **Juvenile** | | | | | | **adult** | | | | | | |
| analyzed mRNA | **mean** | **±SD** | **±SEM** | **median** | **95% CI** | **n** | **mean** | **±SD** | **±SEM** | **median** | **95% CI** | **n** | **p-value** |
| **mNCS-1** | 34.8 | 15.0 | 4.2 | 32.2 | 21.9-48.0 | 13 | 30.2 | 16.7 | 4.5 | 23.7 | 17.8-51.7 | 14 | 0.4020 |

**Supplementary Table 5A/B.**

**Semi-quantitative confocal immunofluorescence analysis.** Data & statistics for graphs shown in Figure 1c/4e (A) and Supplementary Figure 6c/d (B) given as mean, ± SD, ±SEM, median and 95% confidence interval (CI). Number of mice analyzed for each group is given by N (upper part of the table), number of total analyzed neurons is given by n (lower part of the table). Significances according to MWU-test and t-test (A) and two-way ANOVA with Sidak`s multiple comparison test (B) (significant values in bold).

| | **A) relative protein expression in SN vs. VTA of wildtype (C57BL/6J) mice** | | | | | | | | | | | | | |
|---|---|---|---|---|---|---|---|---|---|---|---|---|---|---|
| | **wildtype VTA dopaminergic neurons** | | | | | | **wildtype SN dopaminergic neurons** | | | | | | | |
| | **mean** | **±SD** | **±SEM** | **median** | **95% CI** | **N** | **mean** | **±SD** | **±SEM** | **median** | **95% CI** | **N** | **p-value (MWU)** | **p-value (t-test)** |
| **Cav2.3** | 1.00 | 0.05 | 0.02 | 1.00 | 0.95-1.06 | 4 | 1.17 | 0.04 | 0.02 | 1.17 | 1.12-1.22 | 4 | **0.0286** | **0.0015** |
| **NCS-1** | 1.00 | 0.04 | 0.02 | 1.00 | 0.96-1.05 | 4 | 1.03 | 0.03 | 0.02 | 1.02 | 1.01-1.08 | 4 | 0.2000 | 0.2394 |
| | **wildtype VTA dopaminergic neurons** | | | | | | **wildtype SN dopaminergic neurons** | | | | | | | |
| | **mean** | **±SD** | **±SEM** | **median** | **95% CI** | **n** | **mean** | **±SD** | **±SEM** | **median** | **95% CI** | **n** | **p-value (MWU)** | **p-value (t-test)** |
| **Cav2.3** | 1.00 | 0.17 | 0.01 | 0.98 | 0.96-0.99 | 635 | 1.17 | 0.20 | 0.01 | 1.14 | 1.12-1.16 | 584 | **<0.0001** | **<0.0001** |
| **NCS-1** | 1.00 | 0.18 | 0.01 | 0.96 | 0.95-0.98 | 657 | 1.02 | 0.20 | 0.01 | 0.98 | 0.97-0.99 | 605 | 0.0600 | **0.0353** |

| | **B) relative protein expression in TH-positive SN and VTA neurons of wildtype vs. knockout mice** | | | | | | | | | | | |
|---|---|---|---|---|---|---|---|---|---|---|---|---|
| | **Cav2.3 in Cav2.3 wildtype vs. Cav2.3 knockout mice** | | | | | | **NCS-1 in NCS-1 wildtype vs. NCS-1 knockout mice** | | | | | |
| | **mean** | **±SD** | **±SEM** | **median** | **95% CI** | **n/N** | **mean** | **±SD** | **±SEM** | **median** | **95% CI** | **n/N** |
| **SN DA WT** | 4.31 | 1.00 | 0.06 | 4.13 | 3.96-4.31 | 264/3 | 2.23 | 0.46 | 0.03 | 2.13 | 2.08-2.22 | 257/3 |
| **SN DA KO** | 3.30 | 0.74 | 0.04 | 3.18 | 3.07-3.27 | 344/3 | 1.28 | 0.23 | 0.01 | 1.24 | 1.22-1.28 | 263/3 |
| **VTA DA WT** | 3.69 | 0.86 | 0.05 | 3.53 | 3.39-3.73 | 287/3 | 2.16 | 0.38 | 0.03 | 2.08 | 2.02-2.15 | 226/3 |
| **VTA DA KO** | 3.15 | 0.67 | 0.04 | 3.03 | 2.97-3.11 | 346/3 | 1.34 | 0.28 | 0.02 | 1.28 | 1.24-1.32 | 240/3 |
| **p-value** | SN DA WT vs. SN DA KO: **<0.0001**; VTA DA WT vs. VTA DA KO: **<0.0001**; SN DA WT vs. VTA DA WT: **<0.0001**; SN DA KO vs. VTA DA KO: 0.0891 | | | | | | SN DA WT vs. SN DA KO: **<0.0001**; VTA DA WT vs. VTA DA KO: **<0.0001**; SN DA WT vs. VTA DA WT: 0.2085; SN DA KO vs. VTA DA KO: 0.4512 | | | | | |

Cav2.3: two-way ANOVA: WT vs. KO: **p<0.0001**, SN vs VTA: **p<0.0001**, interaction: **p<0.0001**.
NCS-1: two-way ANOVA: WT vs. KO: **p<0.0001**, SN vs VTA: p=0.7531, interaction: **p=0.0081**;

**Supplementary Table 6A/B/C/D.**

**$Ca^{2+}$ imaging in SN and VTA dopaminergic neurons.** Data & statistics for graphs shown in Figure 2 (A, C), Supplementary Figure 1 (B) and Supplementary Figure 2 (D). n represents number of analyzed dopaminergic neurons. Significances according to * Kruskal Wallis test with Dunn's multiple comparisons test (A/B), #one-sample Wilcoxon test and **MWU test (C, D) (significant values in bold).

| | **A) Actionpotential evoked $Ca^{2+}$ transients at 1.5 Hz pacemaking** | | | | | | | | | | | |
|---|---|---|---|---|---|---|---|---|---|---|---|---|
| | **$\Delta R_{Fura,max}$ (%)** | | | | | | **$area_{Ca2+-transient}$ (AU)** | | | | | |
| | **mean** | **±SD** | **±SEM** | **median** | **95% CI** | **n** | **mean** | **±SD** | **±SEM** | **median** | **95% CI** | **N** |
| **SN DA WT** | 10.33 | 2.98 | 0.77 | 9.31 | 8.03-13.06 | 15 | 415.9 | 122.3 | 31.6 | 388.7 | 295.2-502.2 | 15 |
| **SN DA Cav2.3 KO** | 5.75 | 1.68 | 0.49 | 5.22 | 4.40-7.69 | 12 | 216.4 | 61.26 | 17.7 | 201.7 | 166.2-288.0 | 12 |
| **VTA DA WT** | 3.08 | 0.92 | 0.348 | 3.15 | 1.63-4.46 | 7 | 127.3 | 53.27 | 20.1 | 108.5 | 40.5-206.5 | 7 |
| **p-value*** | SN DA WT vs. Cav2.3 KO: **0.0058**; VTA DA WT vs. SN DA WT: **<0.0001**; VTA DA WT vs. SN DA Cav2.3 KO: 0.1143 | | | | | | SN DA WT vs. Cav2.3 KO: **0.0024**; VTA DA WT vs. SN DA WT: **<0.0001**; VTA DA WT vs. SN DA Cav2.3 KO: 0.2929 | | | | | |

| | **B) Actionpotential parameters at 1.5 Hz pacemaking** | | | | | | | | | | | |
|---|---|---|---|---|---|---|---|---|---|---|---|---|
| | **Spike amplitude [mV]** | | | | | | **Spike maximum [mV]** | | | | | |
| | **mean** | **±SD** | **±SEM** | **median** | **95% CI** | **n** | **mean** | **±SD** | **±SEM** | **median** | **95% CI** | **n** |
| **SN DA WT** | 96.8 | 7.7 | 2.0 | 96.3 | 91.1-101.1 | 15 | 21.1 | 6.9 | 1.8 | 21.7 | 18.3-23.9 | 15 |
| **SN DA Cav2.3 KO** | 82.6 | 11.5 | 3.3 | 83.8 | 72.5-92.2 | 12 | 16.8 | 8.3 | 2.4 | 17.7 | 6.7-23.5 | 12 |
| **VTA DA WT** | 73.2 | 13.1 | 5.0 | 72.2 | 55.9-91.9 | 7 | 9.9 | 10.4 | 3.9 | 11.4 | -7.7-20.1 | 7 |
| **p-value*** | SN DA WT vs. Cav2.3 KO: **0.0141**; VTA DA WT vs. SN DA WT: **0.0009**; VTA DA WT vs. SN DA Cav2.3 KO: 0.7244 | | | | | | SN DA WT vs. Cav2.3 KO: 0.5668; VTA DA WT vs. SN DA WT: **0.0275**; VTA DA WT vs. SN DA Cav2.3 KO: 0.4515 | | | | | |

| | **Spike minimum [mV]** | | | | | | **Spike width @ ½ spike amplitude [ms]** | | | | | |
|---|---|---|---|---|---|---|---|---|---|---|---|---|
| | **mean** | **±SD** | **±SEM** | **median** | **95% CI** | **n** | **mean** | **±SD** | **±SEM** | **median** | **95% CI** | **n** |
| **SN DA WT** | -75.8 | 3.2 | 0.8 | -75.7 | -74.3-76.9 | 15 | 1.3 | 0.1 | 0.03 | 1.3 | 1.2-1.4 | 15 |
| **SN DA Cav2.3 KO** | -65.8 | 4.2 | 1.2 | -66.8 | -68.92--60.27 | 12 | 1.2 | 0.2 | 0.05 | 1.2 | 1.1-1.4 | 12 |
| **VTA DA WT** | -61.0 | 7.1 | 2.7 | -60.3 | -72.2--52.9 | 7 | 1.7 | 0.5 | 0.2 | 1.6 | 1.3-2.1 | 7 |
| **p-value*** | SN DA WT vs. Cav2.3 KO: **0.0003**; VTA DA WT vs. SN DA WT: **0.0001**; VTA DA WT vs. SN DA Cav2.3 KO: >0.9999 | | | | | | SN DA WT vs. Cav2.3 KO: 0.3782; VTA DA WT vs. SN DA WT: 0.0601; VTA DA WT vs. SN DA Cav2.3 KO: **0.0015** | | | | | |

| | Spike width @ threshold[ms] | | | | | | Spike threshold [mV] | | | | | |
|---|---|---|---|---|---|---|---|---|---|---|---|---|
| | mean | ±SD | ±SEM | median | 95% CI | n | mean | ±SD | ±SEM | median | 95% CI | n |
| **SN DA WT** | 1.9 | 0.2 | 2.0 | 1.9 | 1.7-2.0 | 15 | -44.7 | 2.9 | 0.8 | -44.5 | -42.1-46.7 | 15 |
| **SN DA Cav2.3 KO** | 1.8 | 0.2 | 0.05 | 1.8 | 1.7-2.0 | 12 | -40.9 | 2.5 | 0.7 | -41.2 | -42.9--40.2 | 12 |
| **VTA DA WT** | 2.6 | 0.5 | 0.2 | 2.6 | 2.0-3.3 | 7 | -40.6 | 5.2 | 2.0 | -43.0 | -45.3--30.7 | 7 |
| **p-value*** | SN DA WT vs. Cav2.3 KO: >0.9999; VTA DA WT vs. SN DA WT: **0.0021**; VTA DA WT vs. SN DA Cav2.3 KO: **0.0007** | | | | | | SN DA WT vs. Cav2.3 KO: **0.0084**; VTA DA WT vs. SN DA WT: 0.2476; VTA DA WT vs. SN DA Cav2.3 KO: >0.9999 | | | | | |

| | AHP [mV] | | | | | | AHP time [s] | | | | | |
|---|---|---|---|---|---|---|---|---|---|---|---|---|
| | mean | ±SD | ±SEM | median | 95% CI | n | mean | ±SD | ±SEM | median | 95% CI | n |
| **SN DA WT** | 31.1 | 2.5 | 0.6 | 30.1 | 29.1-32.5 | 15 | 0.03 | 0.02 | 0.005 | 0.03 | 0.02-0.05 | 15 |
| **SN DA Cav2.3 KO** | 24.9 | 2.6 | 0.8 | 24.7 | 23.5-27.2 | 12 | 0.01 | 0.01 | 0.002 | 0.01 | 0.01-0.02 | 12 |
| **VTA DA WT** | 20.4 | 4.1 | 1.6 | 17.7 | 16.9-26.9 | 7 | 0.02 | 0.02 | 0.007 | 0.02 | 0.003-0.05 | 7 |
| **p-value*** | SN DA WT vs. Cav2.3 KO: **0.0008**; VTA DA WT vs. SN DA WT: **<0.0001**; VTA DA WT vs. SN DA Cav2.3 KO: 0.4777 | | | | | | SN DA WT vs. Cav2.3 KO: **0.0251**; VTA DA WT vs. SN DA WT: 0.5536; VTA DA WT vs. SN DA Cav2.3 KO: >0.9999 | | | | | |

| | Depolarization Rate [V/s] | | | | | | Repolarization Rate [V/s] | | | | | |
|---|---|---|---|---|---|---|---|---|---|---|---|---|
| | mean | ±SD | ±SEM | median | 95% CI | n | mean | ±SD | ±SEM | median | 95% CI | n |
| **SN DA WT** | 193.5 | 55.8 | 14.4 | 173.2 | 160.8-253.0 | 15 | -72.4 | 9.2 | 2.4 | -71.4 | -65.5-77.8 | 15 |
| **SN DA Cav2.3 KO** | 148.8 | 59.3 | 17.1 | 147.6 | 94.9-179.3 | 12 | -74.2 | 14.2 | 4.1 | -75.4 | -82.9-64.0 | 12 |
| **VTA DA WT** | 90.2 | 45.0 | 17.0 | 92.3 | 32.6-140.7 | 7 | -48.9 | 15.1 | 5.7 | -49.1 | -71.9-27.7 | 7 |
| **p-value*** | SN DA WT vs. Cav2.3 KO: 0.2758; VTA DA WT vs. SN DA WT: **0.0019**; VTA DA WT vs. SN DA Cav2.3 KO: 0.1663 | | | | | | SN DA WT vs. Cav2.3 KO: >0.9999; VTA DA WT vs. SN DA WT: **0.0101**; VTA DA WT vs. SN DA Cav2.3 KO: **0.0059** | | | | | |

| | Frequency before corrections [Hz] | | | | | | Injected current [pA] | | | | | |
|---|---|---|---|---|---|---|---|---|---|---|---|---|
| | mean | ±SD | ±SEM | median | 95% CI | n | mean | ±SD | ±SEM | median | 95% CI | n |
| **SN DA WT** | 3.1 | 1.0 | 0.3 | 2.9 | 2.3-3.7 | 15 | -33.2 | 21.5 | 5.6 | -34.0 | -20.0-45.0 | 15 |
| **SN DA Cav2.3 KO** | 2.9 | 1.7 | 0.5 | 2.6 | 1.4-4.2 | 12 | -25.0 | 20.0 | 5.8 | -18.0 | -64.0-3.0 | 12 |
| **VTA DA WT** | 3.1 | 1.6 | 0.6 | 2.6 | 1.6-5.6 | 7 | -8.4 | 9.1 | 3.4 | -7.0 | -26.0-0.0 | 7 |
| **p-value*** | SN DA WT vs. Cav2.3 KO: >0.9999; VTA DA WT vs. SN DA WT: >0.9999; VTA DA WT vs. SN DA Cav2.3 KO: >0.9999 | | | | | | SN DA WT vs. Cav2.3 KO: 0.7287; VTA DA WT vs. SN DA WT: **0.0102**; VTA DA WT vs. SN DA Cav2.3 KO: 0.1858 | | | | | |

| | SN dopaminergic neurons of Cav2.3 wildtype vs. C57BL/6J wildtype mice | | | | | | | | | | | | |
|---|---|---|---|---|---|---|---|---|---|---|---|---|---|
| | SN dopaminergic neurons of Cav2.3 wildtype mice | | | | | | SN dopaminergic neurons of C57BL/6J wildtype mice | | | | | | |
| | mean | ±SD | ±SEM | median | 95% CI | n | mean | ±SD | ±SEM | median | 95% CI | n | p-value** |
| $\Delta R_{Fura,max}$ [%] | 10.9 | 3.1 | 1.0 | 10.2 | 8.0-13.3 | 10 | 9.1 | 2.7 | 1.2 | 8.3 | 6.7-13.4 | 5 | 0.3097 |
| $area_{Ca2+\text{-}transient}$ [AU] | 422.8 | 135.8 | 42.9 | 415.7 | 283.8-555.6 | 10 | 402.2 | 102.6 | 45.9 | 372.0 | 295.2-548.3 | 5 | 0.9530 |
| Spike amplitude [mV] | 98.8 | 8.6 | 2.7 | 99.8 | 88.6-108.2 | 10 | 92.9 | 3.3 | 1.5 | 94.2 | 88.0-96.3 | 5 | 0.0992 |
| Spike minimum [mV] | -76.1 | 3.9 | 1.2 | -76.5 | -70.9-80.6 | 10 | -74.3 | 2.4 | 1.0 | -74.9 | -70.0-76.6 | 5 | 0.2198 |
| Spike maximum [mV] | 22.7 | 7.6 | 2.4 | 22.8 | 19.1-31.0 | 10 | 17.8 | 4.0 | 1.8 | 18.3 | 12.3-22.8 | 5 | 0.0753 |
| Spike width @ ½ spike amplitude [ms] | 1.3 | 0.1 | 0.05 | 1.3 | 1.2-1.5 | 10 | 1.4 | 0.1 | 0.05 | 1.4 | 1.2-1.5 | 5 | 0.5135 |
| Spike threshold[ms] | -45.4 | 3.1 | 1.0 | -46.2 | -42.1--48.1 | 10 | -43.3 | 2.3 | 1.0 | -42.4 | -41.8--47.3 | 5 | 0.3097 |
| Spike width @threshold [mV] | 1.9 | 0.2 | 0.05 | 1.9 | 1.7-2.0 | 10 | 1.9 | 0.2 | 0.08 | 1.9 | 1.7-2.1 | 5 | 0.6787 |
| AHP [mV] | 30.7 | 2.5 | 0.8 | 30.3 | 28.0-32.9 | 10 | 31.8 | 2.1 | 1.0 | 32.4 | 28.4-34.2 | 5 | 0.3710 |
| AHP time [s] | 0.03 | 0.02 | 0.005 | 0.03 | 0.008-0.05 | 10 | 0.04 | 0.02 | 0.007 | 0.04 | 0.03-0.07 | 5 | 0.0753 |
| Depolarization Rate [V/s] | 212.1 | 58.9 | 18.6 | 211.1 | 161.1-268.9 | 10 | 156.3 | 22.0 | 9.9 | 160.8 | 121.6-179.6 | 5 | 0.0753 |
| Repolarization Rate [V/s] | -74.9 | 9.8 | 3.1 | -73.0 | -65.5-85.1 | 10 | -67.4 | 5.5 | 2.5 | -65.6 | -61.1-74.8 | 5 | 0.2065 |

| | C) Remaining $Ca^{2+}$ transients induced by evoked action potentials in SNX-482 | | | | | | |
|---|---|---|---|---|---|---|---|
| | mean | ±SD | ±SEM | median | 95% CI | n | p-value |
| Remaining $Ca^{2+}$ amplitude in SNX-482 in Cav2.3 wildtype [%] | 74.8 | 16.1 | 6.6 | 73.2 | 51.1-99.1 | 6 | **0.0313**# |
| Remaining $Ca^{2+}$ amplitude in SNX-482 in Cav2.3 knockout [%] | 101.1 | 8.2 | 2.9 | 100.5 | 91.3-116.2 | 8 | 0.9453# |

**p-values for remaining $Ca^{2+}$ amplitude in SNX-482: Cav2.3 WT vs Cav2.3 KO: **0.008**

| | D) SN dopaminergic neurons: evoked spikes in SNX-482 | | | | | | | | | | | | |
|---|---|---|---|---|---|---|---|---|---|---|---|---|---|
| | **Cav2.3 wildtype** | | | | | | **Cav2.3 knockout** | | | | | | |
| | **mean** | **±SD** | **±SEM** | **median** | **95% CI** | **n** | **mean** | **±SD** | **±SEM** | **median** | **95% CI** | **n** | **p-value**** |
| **Amplitude [%]** | 100.7 | 7.2 | 2.9 | 96.6 | 94.8-111.2 | 6 | 101.2 | 6.6 | 2.3 | 101.3 | 92.0-110.9 | 8 | 0.9497 |
| **Width [%]** | 102.5 | 14.8 | 6.0 | 95.8 | 90.2-123.6 | 6 | 106.5 | 4.3 | 1.5 | 107.3 | 100.1-112.4 | 8 | 0.3450 |
| **Vmax [%]** | 131.5 | 62.9 | 25.7 | 118.3 | 50.3-225.8 | 6 | 124.7 | 23.5 | 8.3 | 119.3 | 93.5-155.9 | 8 | 0.9497 |
| **Vmin [%]** | 93.7 | 8.1 | 3.3 | 95.4 | 78.4-101.3 | 6 | 96.0 | 7.8 | 2.8 | 96.8 | 80.3-106.7 | 8 | 0.5728 |
| **Threshold [%]** | 94.7 | 14.5 | 5.9 | 101.5 | 74.2-106.9 | 6 | 97.7 | 10.4 | 3.7 | 98.3 | 77.4-112.2 | 8 | 0.9497 |
| **Width2 [%]** | 108.7 | 16.7 | 6.8 | 102.8 | 92.2-133.3 | 6 | 107.1 | 5.7 | 2.0 | 105.9 | 101.1-116.8 | 8 | 0.6620 |
| **AHP [%]** | 74.5 | 20.9 | 8.5 | 86.2 | 38.0-88.6 | 6 | 97.4 | 10.1 | 3.6 | 98.0 | 82.1-115.0 | 8 | **0.0293** |
| **Depolrate [%]** | 108.2 | 24.8 | 10.1 | 101.9 | 79.5-142.4 | 6 | 97.2 | 10.6 | 3.8 | 102.8 | 79.4-108.0 | 8 | 0.4136 |
| **Repolrate [%]** | 95.3 | 10.0 | 4.09 | 98.2 | 82.4-105.5 | 6 | 94.9 | 6.7 | 2.4 | 97.2 | 84.0-101.4 | 8 | 0.9497 |

**Supplementary Table 7.**

**Whole cell voltage-clamp analysis.** Data & statistics for graphs shown in Supplementary Figure 3 given as mean, ± SD, ±SEM, median and 95% confidence interval (CI). n represents number of analyzed SN dopaminergic neurons from N mice. Significances according to MWU- test (significant values in bold).

| | Voltage activated $Ca^{2+}$ currents | | | | | | | | | | | | |
|---|---|---|---|---|---|---|---|---|---|---|---|---|---|
| | control | | | | | | SNX-482 | | | | | | |
| | mean | ±SD | ±SEM | median | 95% CI | n/N | mean | ±SD | ±SEM | median | 95% CI | n/N | p-value (MWU) |
| **AUC [nA*mV]** | 120.3 | 22.3 | 8.4 | 118.1 | 104.2-128.9 | 7/3 | 83.0 | 21.0 | 7.9 | 81.0 | 58.0-97.4 | 7/3 | **0.0070** |
| **ΔAUC (SNX-sensitive area) [%]** | | | | | | | 29.5 | 21.1 | 8.0 | 29.0 | -11.2-51.1 | 7/3 | |
| **ΔGmax (SNX-sensitive conductance) [%]** | | | | | | | 32.1 | 15.5 | 5.9 | 29.5 | 4.8-49.1 | 7/3 | |

I-V curve:
parameters fit of means:

control: $G_{max}$ = 0.042 µS; $V_{rev}$ = 47.77 mV; $V_{0.5}$ = -27.83 mV; k = 5.89

SNX-482: $G_{max}$ = 0.025 µS; $V_{rev}$ = 51.38 mV; $V_{0.5}$ = -30.77 mV; k = 6.08

parameters mean fit of individual fits (mean ± SD):

control: $G_{max}$ = 0.04 ± 0.02 µS; $V_{rev}$ = 49.77 ± 8.14 mV; $V_{0.5}$ = -28.39 ± 5.57 mV; k = 5.10 ± 1.08

SNX-482: $G_{max}$ = 0.03 ± 0.01 µS; $V_{rev}$ = 53.02 ± 10.69 mV; $V_{0.5}$ = -29.86 ± 6.77 mV; k = 4.95 ± 1.87

Max. conductance
parameters fit of means:

control: $G_{max}$ = 15.84 nS; $V_{0.5}$ = -31.85 mV; k = 4.78

SNX-482: $G_{max}$ = 10.18 nS; $V_{0.5}$ = -33.32 mV; k = 5.11

ΔG (SNX- sensitive conductance): 5.66 nS; 35.73 %

parameters mean fit of individual fits (mean ± SD):

control: $G_{max}$ = 16.20 ± 4.36 nS; $V_{0.5}$ = -31.55 ± 5.92 mV; k = 3.81 ± 1.35

SNX-482: $G_{max}$ = 10.70 ± 2.35 nS; $V_{0.5}$ = -31.43 ± 6.34 mV; k = 4.27 ± 2.19

ΔG (SNX- sensitive conductance): 5.49 ± 3.54 nS; 32.11 ± 15.53 %

**Supplementary Table 8A/B/C/D.**

**Densitometry and cell count data.** Data & statistics for graphs shown in Figure 3a/b (A), Figure 3c (B), Figure 4a (C), Figure 4b (D), Supplementary Figure 4 (A-D) and Supplementary Figure 5 (A) given as mean, ± SD, ±SEM, median and 95% confidence interval (CI). n represents number of analyzed mice (except for bootstrapping data). Significances according to *MWU- test (significant values in bold). +t-test was only used for relative remaining fibers and neurons without bootstrapping analysis. Relative remaining TH signals (%) in the dorsal (DS) and ventral striatum (VS) and relative remaining SN dopaminergic and VTA dopaminergic neurons were calculated in addition to normalization to the respective mean values of saline treated group (see in A-D as indicated) as bootstrapping approach (see in A-D as indicated and methods for details). ‘Two-way ANOVA was used to test significant difference between unbiased stereology and automated cell counting.

| | **A) Cell count data of Cav2.3 wildtype vs. Cav2.3 knockout under chronic MPTP** | | | | | | | | | | | | |
|---|---|---|---|---|---|---|---|---|---|---|---|---|---|
| | **Substantia nigra (SN) - stereology of TH-pos. neurons** | | | | | | | | | | | | |
| | **Control (saline)** | | | | | | **MPTP** | | | | | | |
| | **mean** | **±SD** | **±SEM** | **median** | **95% CI** | **n** | **Mean** | **±SD** | **±SEM** | **median** | **95% CI** | **n** | **p-value*** |
| **Cav2.3 WT** | 3959 | 643 | 215 | 3826 | 3637-4622 | 9 | 2535 | 890 | 247 | 2906 | 1566-3542 | 13 | **0.0002** |
| **Cav2.3 KO** | 4170 | 520 | 174 | 4264 | 3475-4833 | 9 | 3990 | 454 | 144 | 3938 | 3618-4286 | 10 | 0.4002 |
| | **SN - automated count of all Hematoxylin-stained neurons (dopaminergic + GABAergic)** | | | | | | | | | | | | |
| | **Control (saline)** | | | | | | **MPTP** | | | | | | |
| | **mean** | **±SD** | **±SEM** | **median** | **95% CI** | **n** | **Mean** | **±SD** | **±SEM** | **median** | **95% CI** | **n** | **p-value*** |
| **Cav2.3 WT** | 13121 | 2726 | 909 | 14078 | 11026-15217 | 9 | 9918 | 2243 | 648 | 9411 | 8492-11343 | 12 | **0.0118** |
| **Cav2.3 KO** | 14392 | 2336 | 779 | 14338 | 12596-16188 | 9 | 14365 | 1426 | 451 | 14757 | 13344-15385 | 10 | 0.9682 |
| | **Ventral tegmental area (VTA) – stereology of TH-pos. neurons** | | | | | | | | | | | | |
| | **Control (saline)** | | | | | | **MPTP** | | | | | | |
| | **mean** | **±SD** | **±SEM** | **median** | **95% CI** | **n** | **Mean** | **±SD** | **±SEM** | **median** | **95% CI** | **n** | **p-value*** |
| **Cav2.3 WT** | 6806 | 825 | 275 | 6731 | 5934-7910 | 9 | 6057 | 1209 | 335 | 5600 | 5238-7683 | 13 | 0.0708 |
| **Cav2.3 KO** | 7480 | 931 | 310 | 7547 | 6679-8285 | 9 | 6689 | 660 | 209 | 6642 | 6071-7167 | 10 | 0.0535 |

| | Remaining neurons [%] | | | | | | | | | | | | |
|---|---|---|---|---|---|---|---|---|---|---|---|---|---|
| | Cav2.3 wildtype MPTP | | | | | | Cav2.3 knockout MPTP | | | | | | |
| | **mean** | **±SD** | **±SEM** | **median** | **95% CI** | **n** | **mean** | **±SD** | **±SEM** | **median** | **95% CI** | **n** | **p-value$^{+}$** |
| **SN - TH stereology** | 64.1 | 23.0 | 6.4 | 74.6 | 40.2-86.7 | 13 | 95.4 | 11.0 | 3.5 | 93.8 | 86.2-109.3 | 10 | **0.0007** |
| **SN - TH automat. count** | 58.2 | 23.8 | 6.9 | 65.2 | 33.8-80.4 | 12 | 87.9 | 8.8 | 2.8 | 86.9 | 79.3-94.3 | 10 | **0.0013** |
| **SN - all neurons count** | 75.5 | 18.5 | 5.3 | 73.4 | 63.7-87.2 | 12 | 99.3 | 8.4 | 2.7 | 100.2 | 93.3-105.4 | 10 | **0.0012** |
| **VTA - TH stereology** | 95.8 | 19.1 | 5.3 | 88.6 | 82.8-121.5 | 13 | 96.7 | 9.5 | 3.0 | 96.1 | 87.8-103.7 | 10 | 0.8880 |
| | **Remaining neurons [%] (bootstrapping approach)** | | | | | | | | | | | | |
| | **Cav2.3 wildtype MPTP** | | | | | | **Cav2.3 knockout MPTP** | | | | | | |
| | **mean** | **±SD** | **±SEM** | **median** | **95% CI** | **n** | **mean** | **±SD** | **±SEM** | **median** | **95% CI** | **n** | **p-value*** |
| **SN - TH stereology** | 65.8 | 25.6 | 2.4 | 67.8 | 57.7-75.9 | 117 | 97.1 | 16.0 | 1.7 | 95.3 | 90.3-99.7 | 90 | **<0.0001** |
| **SN - TH automat. count** | 59.0 | 23.1 | 2.2 | 63.5 | 57.3-70.1 | 108 | 89.2 | 13.4 | 1.4 | 88.2 | 86.1-91.5 | 90 | **<0.0001** |
| **SN - all neurons count** | 79.7 | 28.0 | 2.7 | 72.6 | 66.6-79.3 | 108 | 102.6 | 20.9 | 2.2 | 101.2 | 94.4-104.6 | 90 | **<0.0001** |
| **VTA - TH stereology** | 90.1 | 20.2 | 1.9 | 88.3 | 83.2-92.3 | 117 | 90.7 | 13.7 | 1.4 | 89.1 | 86.5-93.2 | 90 | 0.5003 |

‘p-value for remaining neurons (see Fig. S4a left) Stereology vs. Automated count: 0.2393 (two-way ANOVA)

| | B) Densitometric data for Cav2.3 wildtype vs. Cav2.3 knockout under chronic MPTP | | | | | | | | | | | | |
|---|---|---|---|---|---|---|---|---|---|---|---|---|---|
| | **Dorsal Striatum (DS)** | | | | | | | | | | | | |
| | **control** | | | | | | **MPTP** | | | | | | |
| | **mean** | **±SD** | **±SEM** | **median** | **95% CI** | **n** | **mean** | **±SD** | **±SEM** | **median** | **95% CI** | **n** | **p-value*** |
| **Cav2.3 WT** | 32.9 | 2.7 | 0.9 | 32.8 | 30.2-36.0 | 9 | 14.7 | 7.0 | 1.9 | 16.8 | 8.1-21.3 | 13 | **<0.0001** |
| **Cav2.3 KO** | 36.1 | 5.6 | 1.9 | 33.7 | 31.2-42.3 | 9 | 23.4 | 7.2 | 2.1 | 22.8 | 19.8-30.0 | 12 | **0.0005** |
| | **Ventral Striatum (VS)** | | | | | | | | | | | | |
| | **control** | | | | | | **MPTP** | | | | | | |
| | **mean** | **±SD** | **±SEM** | **median** | **95% CI** | **n** | **mean** | **±SD** | **±SEM** | **median** | **95% CI** | **n** | **p-value*** |
| **Cav2.3 WT** | 33.4 | 4.9 | 1.6 | 32.5 | 28.0-39.5 | 9 | 24.9 | 3.7 | 1.0 | 25.0 | 21.5-28.1 | 13 | **0.0004** |
| **Cav2.3 KO** | 40.5 | 8.1 | 2.7 | 37.1 | 33.2-51.6 | 9 | 31.2 | 5.6 | 1.6 | 29.1 | 26.4-37.3 | 12 | **0.0227** |

| | Remaining fibers [%] | | | | | | | | | | | | |
|---|---|---|---|---|---|---|---|---|---|---|---|---|---|
| | Cav2.3 wildtype MPTP | | | | | | Cav2.3 knockout MPTP | | | | | | |
| | mean | ±SD | ±SEM | median | 95% CI | n | mean | ±SD | ±SEM | median | 95% CI | n | p-value+ |
| **Cav2.3: DS** | 44.7 | 21.4 | 5.9 | 50.8 | 24.5-64.2 | 13 | 66.0 | 19.4 | 5.6 | 65.4 | 57.0-83.9 | 12 | **0.0159** |
| **Cav2.3: VS** | 74.5 | 12.3 | 3.4 | 76.7 | 62.7-86.1 | 13 | 78.2 | 13.5 | 3.9 | 75.8 | 67.3-90.4 | 12 | 0.4817 |
| | **Remaining fibers [%] (bootstrapping approach)** | | | | | | | | | | | | |
| | **Cav2.3 wildtype MPTP** | | | | | | **Cav2.3 knockout MPTP** | | | | | | |
| | **mean** | **±SD** | **±SEM** | **median** | **95% CI** | **n** | **mean** | **±SD** | **±SEM** | **median** | **95% CI** | **n** | **p-value*** |
| **Cav2.3: DS** | 45.0 | 21.0 | 1.9 | 48.0 | 45.6-51.9 | 117 | 66.2 | 21.8 | 2.1 | 66.4 | 61.6-70.3 | 108 | **<0.0001** |
| **Cav2.3: VS** | 76.0 | 15.2 | 1.4 | 73.4 | 70.7-77.9 | 117 | 79.5 | 19.4 | 1.9 | 77.6 | 74.8-80.4 | 108 | 0.2005 |

| | C) Stereological cell count data of NCS-1 wildtype vs. NCS-1 knockout under chronic MPTP | | | | | | | | | | | | |
|---|---|---|---|---|---|---|---|---|---|---|---|---|---|
| | **Substantia nigra (SN)** | | | | | | | | | | | | |
| | **control** | | | | | | **MPTP** | | | | | | |
| | **mean** | **±SD** | **±SEM** | **median** | **95% CI** | **n** | **mean** | **±SD** | **±SEM** | **median** | **95% CI** | **n** | **p-value*** |
| **NCS-1 WT** | 4351 | 780 | 208 | 4224 | 3693-4741 | 14 | 2070 | 688 | 191 | 1839 | 1501-2761 | 13 | **<0.0001** |
| **NCS-1 KO** | 3519 | 289 | 91 | 3454 | 3269-3900 | 10 | 1257 | 364 | 110 | 1332 | 726-1628 | 11 | **<0.0001** |
| | **Ventral tegmental area (VTA)** | | | | | | | | | | | | |
| | **control** | | | | | | **MPTP** | | | | | | |
| | **mean** | **±SD** | **±SEM** | **median** | **95% CI** | **n** | **mean** | **±SD** | **±SEM** | **median** | **95% CI** | **n** | **p-value*** |
| **NCS-1 WT** | 6499 | 1222 | 327 | 6414 | 5587-7301 | 14 | 5045 | 1250 | 347 | 4763 | 3978-6255 | 13 | **0.0091** |
| **NCS-1 KO** | 6217 | 911 | 288 | 6057 | 5327-6753 | 10 | 4076 | 1009 | 304 | 4333 | 2549-4851 | 11 | **<0.0001** |
| | **Remaining neurons [%]** | | | | | | | | | | | | |
| | **NCS-1 wildtype MPTP** | | | | | | **NCS-1 knockout MPTP** | | | | | | |
| | **mean** | **±SD** | **±SEM** | **median** | **95% CI** | **n** | **mean** | **±SD** | **±SEM** | **median** | **95% CI** | **n** | **p-value+** |
| **NCS-1: SN** | 47.6 | 15.8 | 4.4 | 42.2 | 34.5-63.4 | 13 | 35.7 | 10.0 | 3.0 | 36.6 | 21.4-45.7 | 11 | **0.0431** |
| **NCS-1: VTA** | 77.8 | 19.9 | 5.5 | 72.2 | 60.3-98.3 | 13 | 65.6 | 16.2 | 4.9 | 70.0 | 40.8-77.7 | 11 | 0.1169 |
| | **Remaining neurons [%] (bootstrapping approach)** | | | | | | | | | | | | |
| | **NCS-1 wildtype MPTP** | | | | | | **NCS-1 knockout MPTP** | | | | | | |
| | **mean** | **±SD** | **±SEM** | **median** | **95% CI** | **n** | **mean** | **±SD** | **±SEM** | **median** | **95% CI** | **n** | **p-value*** |
| **NCS-1: SN** | 49.0 | 17.9 | 1.3 | 45.0 | 41.1-49.8 | 182 | 35.9 | 10.2 | 1.0 | 37.5 | 33.5-40.6 | 110 | **<0.0001** |
| **NCS-1: VTA** | 80.1 | 24.1 | 1.8 | 76.5 | 73.1-84.4 | 182 | 66.7 | 18.0 | 1.7 | 70.8 | 68.6-74.7 | 110 | **<0.0001** |

| | D) Densitometric data for NCS-1 wildtype vs. NCS-1 knockout under chronic MPTP | | | | | | | | | | | | |
|---|---|---|---|---|---|---|---|---|---|---|---|---|---|
| | **Dorsal Striatum (DS)** | | | | | | | | | | | | |
| | **control** | | | | | | **MPTP** | | | | | | |
| | **mean** | **±SD** | **±SEM** | **median** | **95% CI** | **n** | **mean** | **±SD** | **±SEM** | **median** | **95% CI** | **n** | **p-value*** |
| **NCS-1 WT** | 32.8 | 5.1 | 1.4 | 32.1 | 28.1-36.4 | 14 | 13.8 | 6.9 | 1.9 | 15.2 | 5.8-20.6 | 13 | **<0.0001** |
| **NCS-1 KO** | 31.9 | 6.1 | 1.9 | 29.9 | 25.9-39.4 | 10 | 7.4 | 4.6 | 1.4 | 6.0 | 3.5-15.3 | 11 | **<0.0001** |
| | **Ventral Striatum (VS)** | | | | | | | | | | | | |
| | **control** | | | | | | **MPTP** | | | | | | |
| | **mean** | **±SD** | **±SEM** | **median** | **95% CI** | **n** | **mean** | **±SD** | **±SEM** | **median** | **95% CI** | **n** | **p-value*** |
| **NCS-1 WT** | 32.7 | 6.4 | 1.7 | 31.7 | 25.8-39.4 | 14 | 26.8 | 5.1 | 1.4 | 28.0 | 22.4-33.0 | 13 | **0.0193** |
| **NCS-1 KO** | 36.2 | 9.1 | 2.9 | 33.8 | 27.9-45.4 | 10 | 27.7 | 6.7 | 2.0 | 27.2 | 20.6-36.2 | 11 | **0.0357** |
| | **Remaining fibers [%]** | | | | | | | | | | | | |
| | **NCS-1 wildtype MPTP** | | | | | | **NCS-1 knockout MPTP** | | | | | | |
| | **mean** | **±SD** | **±SEM** | **median** | **95% CI** | **n** | **mean** | **±SD** | **±SEM** | **median** | **95% CI** | **n** | **p-value$^{+}$** |
| **NCS-1: DS** | 42.2 | 21.3 | 5.9 | 46.4 | 17.6-63.1 | 13 | 23.7 | 15.5 | 4.7 | 18.0 | 10.4-50.1 | 11 | **0.0253** |
| **NCS-1: VS** | 81.2 | 11.3 | 3.1 | 77.7 | 71.3-93.7 | 13 | 76.8 | 19.7 | 6.0 | 76.6 | 55.7-102.2 | 11 | 0.4987 |
| | **Remaining fibers [%] (bootstrapping approach)** | | | | | | | | | | | | |
| | **NCS-1 wildtype MPTP** | | | | | | **NCS-1 knockout MPTP** | | | | | | |
| | **mean** | **±SD** | **±SEM** | **median** | **95% CI** | **n** | **mean** | **±SD** | **±SEM** | **median** | **95% CI** | **n** | **p-value*** |
| **NCS-1: DS** | 43.0 | 21.7 | 1.6 | 43.3 | 33.7-51.8 | 182 | 24.0 | 15.1 | 1.4 | 19.6 | 17.8-21.3 | 110 | **<0.0001** |
| **NCS-1: VS** | 84.9 | 22.7 | 1.7 | 80.8 | 76.6-87.7 | 182 | 80.5 | 26.2 | 2.5 | 76.1 | 70.5-83.4 | 110 | 0.0730 |

**Supplementary Table 9A/B.**

**A) Details of human skin biopsy donors for generation of iPSC derived dopaminergic neurons of GBA-1 Parkinson's disease patients and controls.**

| Donor ID | iPSC clone | Genotype | Age & gender |
|---|---|---|---|
| SFC065 | 3 | Control | 65M |
| SFC856 | 4 | Control | 78F |
| SFC068 | 1 | Control | 67 M |
| SFC067 | 1 | Control | 72 M |
| MK088 | 1 | N370S/wt | 46 M |
| MK071 | 3 | N370S/wt | 81 F |
| SFC871 | 4 | N370S/wt | 70 F |

**B) Relative protein levels in iPSC derived dopaminergic neurons of human GBA-1 Parkinson's disease patients and controls.** Western blot derived data & statistics for graphs shown in Figure 5b given as mean, ± SD, ±SEM, median and 95% confidence interval (CI). n represents number of analyzed samples from 6 differentiation (derived from N=4 control and N=3 GBA-1 Parkinson's disease patients, see Suppl. Tab. S8A). Significances according to MWU and t-test (significant values in bold).

| | Normalized protein levels | | | | | | | | | | | | | |
|---|---|---|---|---|---|---|---|---|---|---|---|---|---|---|
| | Control subjects | | | | | | GBA-1 Parkinson's disease patients | | | | | | | |
| | mean | ±SD | ±SEM | median | 95% CI | n | mean | ±SD | ±SEM | median | 95% CI | n | p-value (MWU) | p-value (t-test) |
| **Cav2.3** | 1.00 | 0.11 | 0.04 | 1.00 | 0.79-1.21 | 8 | 1.02 | 0.36 | 0.12 | 0.92 | 0.61-1.41 | 10 | 0.6184 | 0.8835 |
| **NCS-1** | 1.00 | 0.15 | 0.05 | 1.00 | 0.73-1.27 | 8 | 0.58 | 0.34 | 0.11 | 0.52 | 0.28-0.99 | 10 | **0.0074** | **0.0053** |
| **Cav2.3/ NCS-1** | 1.00 | 0.05 | 0.02 | 1.00 | 0.94-1.07 | 8 | 2.18 | 1.06 | 0.33 | 1.86 | 0.93-3.43 | 10 | **0.0313** | **0.0064** |

## Supplementary References


1 Ng, E. *et al.* Neuronal calcium sensor-1 deletion in the mouse decreases motivation and dopamine release in the nucleus accumbens. *Behav Brain Res* **301**, 213-225 (2016).

2 Duda, J., Fauler, M., Grundemann, J. & Liss, B. Cell-Specific RNA Quantification in Human SN DA Neurons from Heterogeneous Post-mortem Midbrain Samples by UV-Laser Microdissection and RT-qPCR. *Methods Mol Biol* **1723**, 335-360 (2018).

3 Grundemann, J., Schlaudraff, F. & Liss, B. UV-laser microdissection and mRNA expression analysis of individual neurons from postmortem Parkinson's disease brains. *Methods Mol Biol* **755**, 363-374 (2011).

4 Pereverzev, A. *et al.* Disturbances in glucose-tolerance, insulin-release, and stress-induced hyperglycemia upon disruption of the Ca(v)2.3 (alpha 1E) subunit of voltage-gated Ca(2+) channels. *Mol Endocrinol* **16**, 884-895 (2002).

5 Liss, B. *et al.* Tuning pacemaker frequency of individual dopaminergic neurons by Kv4.3L and KChip3.1 transcription. *EMBO J* **20**, 5715-5724 (2001).

6 Liss, B. Improved quantitative real-time RT-PCR for expression profiling of individual cells. *Nucleic Acids Res* **30**, e89 (2002).

7 Eisener-Dorman, A. F., Lawrence, D. A. & Bolivar, V. J. Cautionary insights on knockout mouse studies: the gene or not the gene? *Brain Behav Immun* **23**, 318-324 (2009).

8 Dragicevic, E. *et al.* Cav1.3 channels control D2-autoreceptor responses via NCS-1 in substantia nigra dopamine neurons. *Brain* **137**, 2287-2302 (2014).

9 Ortner, N. J. *et al.* Lower affinity of isradipine for L-type Ca2+ channels during substantia nigra dopamine neuron-like activity: implications for neuroprotection in Parkinson's disease. *J Neurosci* **37**, 6761-6777 (2017).

10 Poetschke, C. *et al.* Compensatory T-type Ca2+ channel activity alters D2-autoreceptor responses of Substantia nigra dopamine neurons from Cav1.3 L-type Ca2+ channel KO mice. *Sci Rep* **5**, 13688 (2015).

11 Wennemuth, G., Westenbroek, R. E., Xu, T., Hille, B. & Babcock, D. F. CaV2.2 and CaV2.3 (N- and R-type) Ca2+ channels in depolarization-evoked entry of Ca2+ into mouse sperm. *J Biol Chem* **275**, 21210-21217 (2000).

12 Liss, B. *et al.* K-ATP channels promote the differential degeneration of dopaminergic midbrain neurons. *Nat Neurosci* **8**, 1742-1751 (2005).

13 Meredith, G. E., Totterdell, S., Beales, M. & Meshul, C. K. Impaired glutamate homeostasis and programmed cell death in a chronic MPTP mouse model of Parkinson's disease. *Exp Neurol* **219**, 334-340 (2009).

14 Sun, H. *et al.* Aquaporin-4 mediates communication between astrocyte and microglia: Implications of neuroinflammation in experimental Parkinson's disease. *Neuroscience* **317**, 65-75 (2016).

15 Janakiraman, U. *et al.* Influences of Chronic Mild Stress Exposure on Motor, Non-Motor Impairments and Neurochemical Variables in Specific Brain Areas of MPTP/Probenecid Induced Neurotoxicity in Mice. *PLoS One* **11**, e0146671 (2016).

16 Directives. Directive 2010/63/EU of the European Parliament and of the Council of 22 September 2010 on the protection of animals used for scientific purposes. *Official Journal of the European Union* (2010).

17 Meredith, G. E. & Rademacher, D. J. MPTP mouse models of Parkinson's disease: an update. *J Parkinsons Dis* **1**, 19-33 (2011).

18 Jackson-Lewis, V. & Przedborski, S. Protocol for the MPTP mouse model of Parkinson's disease. *Nat Protoc* **2**, 141-151 (2007).

19 Blesa, J. & Przedborski, S. Parkinson's disease: animal models and dopaminergic cell vulnerability. *Front Neuroanat* **8**, 155 (2014).

20 Tieu, K. A guide to neurotoxic animal models of Parkinson's disease. *Cold Spring Harb Perspect Med* **1**, a009316 (2011).

21 Meredith, G. E., Totterdell, S., Potashkin, J. A. & Surmeier, D. J. Modeling PD pathogenesis in mice: advantages of a chronic MPTP protocol. *Parkinsonism Relat Disord* **14 Suppl 2**, S112-115 (2008).

22 Schildknecht, S., Di Monte, D. A., Pape, R., Tieu, K. & Leist, M. Tipping Points and Endogenous Determinants of Nigrostriatal Degeneration by MPTP. *Trends Pharmacol Sci* **38**, 541-555 (2017).

23 Jackson-Lewis, V., Blesa, J. & Przedborski, S. Animal models of Parkinson's disease. *Parkinsonism Relat Disord* **18 Suppl 1**, S183-185 (2012).

24 Ilijic, E., Guzman, J. N. & Surmeier, D. J. The L-type channel antagonist isradipine is neuroprotective in a mouse model of Parkinson's disease. *Neurobiol Dis* **43**, 364-371 (2011).

25 Grandi, L. C., Di Giovanni, G. & Galati, S. Animal models of early-stage Parkinson's disease and acute dopamine deficiency to study compensatory neurodegenerative mechanisms. *J Neurosci Methods* **308**, 205-218 (2018).

26 Krabbe, S. *et al.* Increased dopamine D2 receptor activity in the striatum alters the firing pattern of dopamine neurons in the ventral tegmental area. *Proc Natl Acad Sci U S A* **112**, E1498-1506 (2015).

27 Paxinos, G. & Franklin, K. The Mouse Brain in Stereotaxic Coordinates (Academic. New York). (2007).

28 Hess, M. E. *et al.* The fat mass and obesity associated gene (Fto) regulates activity of the dopaminergic midbrain circuitry. *Nat Neurosci* **16**, 1042-1048 (2013).

29 Lammel, S. *et al.* Unique properties of mesoprefrontal neurons within a dual mesocorticolimbic dopamine system. *Neuron* **57**, 760-773 (2008).

30 Neuhoff, H., Neu, A., Liss, B. & Roeper, J. I(h) channels contribute to the different functional properties of identified dopaminergic subpopulations in the midbrain. *J Neurosci* **22**, 1290-1302 (2002).

31 Horn, R. & Marty, A. Muscarinic activation of ionic currents measured by a new whole-cell recording method. *J Gen Physiol* **92**, 145-159 (1988).

32 Akaike, N. & Harata, N. Nystatin perforated patch recording and its applications to analyses of intracellular mechanisms. *Jpn J Physiol* **44**, 433-473 (1994).

33 Lindau, M. & Fernandez, J. M. A patch-clamp study of histamine-secreting cells. *J Gen Physiol* **88**, 349-368 (1986).

34 Kyrozis, A. & Reichling, D. B. Perforated-patch recording with gramicidin avoids artifactual changes in intracellular chloride concentration. *J Neurosci Methods* **57**, 27-35 (1995).

35 Kimm, T. & Bean, B. P. Inhibition of A-type potassium current by the peptide toxin SNX-482. *J Neurosci* **34**, 9182-9189 (2014).
36 Subramaniam, M. *et al.* Mutant alpha-synuclein enhances firing frequencies in dopamine substantia nigra neurons by oxidative impairment of A-type potassium channels. *J Neurosci* **34**, 13586-13599 (2014).
37 Bourinet, E. *et al.* The alpha 1E calcium channel exhibits permeation properties similar to low-voltage-activated calcium channels. *J Neurosci* **16**, 4983-4993 (1996).
38 Schlaudraff, F. *et al.* Orchestrated increase of dopamine and PARK mRNAs but not miR-133b in dopamine neurons in Parkinson's disease. *Neurobiol Aging* **35**, 2302-2315 (2014).
39 Weiergraber, M. *et al.* Altered seizure susceptibility in mice lacking the Ca(v)2.3 E-type Ca2+ channel. *Epilepsia* **47**, 839-850 (2006).
40 Weiergraber, M. *et al.* Immunodetection of alpha1E voltage-gated Ca(2+) channel in chromogranin-positive muscle cells of rat heart, and in distal tubules of human kidney. *J Histochem Cytochem* **48**, 807-819 (2000).
41 Penttinen, A. M. *et al.* Implementation of Deep Neural Networks to Count Dopamine Neurons in Substantia Nigra. *Eur J Neurosci* (2018).
42 Wolfart, J., Neuhoff, H., Franz, O. & Roeper, J. Differential expression of the small-conductance, calcium-activated potassium channel SK3 is critical for pacemaker control in dopaminergic midbrain neurons. *J Neurosci* **21**, 3443-3456 (2001).
43 Schindelin, J. *et al.* Fiji: an open-source platform for biological-image analysis. *Nat Methods* **9**, 676-682 (2012).
44 O'Callaghan, D. W., Hasdemir, B., Leighton, M. & Burgoyne, R. D. Residues within the myristoylation motif determine intracellular targeting of the neuronal Ca2+ sensor protein KChIP1 to post-ER transport vesicles and traffic of Kv4 K+ channels. *J Cell Sci* **116**, 4833-4845 (2003).
45 Nakao, S., Wakabayashi, S. & Nakamura, T. Y. Stimulus-dependent regulation of nuclear Ca2+ signaling in cardiomyocytes: a role of neuronal calcium sensor-1. *PloS one* **10**, e0125050 (2015).
46 Fernandes, H. J. *et al.* ER Stress and Autophagic Perturbations Lead to Elevated Extracellular alpha-Synuclein in GBA-N370S Parkinson's iPSC-Derived Dopamine Neurons. *Stem Cell Reports* **6**, 342-356 (2016).
47 Beevers, J. E. *et al.* MAPT Genetic Variation and Neuronal Maturity Alter Isoform Expression Affecting Axonal Transport in iPSC-Derived Dopamine Neurons. *Stem Cell Reports* **9**, 587-599 (2017).
48 Kriks, S. *et al.* Dopamine neurons derived from human ES cells efficiently engraft in animal models of Parkinson's disease. *Nature* **480**, 547-551 (2011).
49 Grabsch, H. *et al.* Immunohistochemical detection of alpha1E voltage-gated Ca(2+) channel isoforms in cerebellum, INS-1 cells, and neuroendocrine cells of the digestive system. *J Histochem Cytochem* **47**, 981-994 (1999).
