# Supplementary material for: Cav2.3 channels contribute to dopaminergic neuron loss in a model of Parkinson’s disease
Source: Nat Commun. 2019 Nov 8;10:5094. doi: 10.1038/s41467-019-12834-x (PMC6841684; doi:10.1038/s41467-019-12834-x)
Supplement: Supplementary file 2 — Reporting Summary [file 41467_2019_12834_MOESM2_ESM.pdf]

nature research

Corresponding author(s): Prof. Birgit Liss, NCOMMS-17-28115B-Z

Last updated by author(s): Aug 30, 2019

Reporting Summary

Nature Research wishes to improve the reproducibility of the work that we publish. This form provides structure for consistency and transparency in reporting. For further information on Nature Research policies, see Authors & Referees and the Editorial Policy Checklist.

Statistics

For all statistical analyses, confirm that the following items are present in the figure legend, table legend, main text, or Methods section.

n/a	Confirmed	
☐	☒	The exact sample size (n) for each experimental group/condition, given as a discrete number and unit of measurement
☐	☒	A statement on whether measurements were taken from distinct samples or whether the same sample was measured repeatedly
☐	☒	The statistical test(s) used AND whether they are one- or two-sided<br>*Only common tests should be described solely by name; describe more complex techniques in the Methods section.*
☒	☐	A description of all covariates tested
☐	☒	A description of any assumptions or corrections, such as tests of normality and adjustment for multiple comparisons
☐	☒	A full description of the statistical parameters including central tendency (e.g. means) or other basic estimates (e.g. regression coefficient) AND variation (e.g. standard deviation) or associated estimates of uncertainty (e.g. confidence intervals)
☐	☒	For null hypothesis testing, the test statistic (e.g. F, t, r) with confidence intervals, effect sizes, degrees of freedom and P value noted<br>*Give P values as exact values whenever suitable.*
☒	☐	For Bayesian analysis, information on the choice of priors and Markov chain Monte Carlo settings
☒	☐	For hierarchical and complex designs, identification of the appropriate level for tests and full reporting of outcomes
☒	☐	Estimates of effect sizes (e.g. Cohen's d, Pearson's r), indicating how they were calculated

Our web collection on statistics for biologists contains articles on many of the points above.

Software and code

Policy information about availability of computer code

Data collection	MC_Rack (Multichannel Systems), PatchMaster (HEKA), StereoInvestigator (MBF Bioscience), Fiji, ImageJ (NIH), SDS2.4 (ThermoFisher Scientific), QuantStudio Design and Analysis Software (ThermoFisher Scientific), Aiforia Cloud, Wolution platform, ZEN (Carl Zeiss)
Data analysis	Data analysis and graphical illustrations were performed using FitMaster software (HEKA Electronics), GraphPad Prism 7 (GraphPad Software, Inc.), Adobe Illustrator CC2015.3 (Adobe Systems Software), Igor Pro 6 (Wavemetrics Inc.), Spike2 (CED), Neuroexplorer (Nex Technologies), SDS 2.4 (Applied Biosystems), StereoInvestigator (MBF Bioscience), and Fiji (https://imagej.net/Fiji)114 software.

For manuscripts utilizing custom algorithms or software that are central to the research but not yet described in published literature, software must be made available to editors/reviewers. We strongly encourage code deposition in a community repository (e.g. GitHub). See the Nature Research guidelines for submitting code & software for further information.

Data

Policy information about availability of data

All manuscripts must include a data availability statement. This statement should provide the following information, where applicable:

- Accession codes, unique identifiers, or web links for publicly available datasets
- A list of figures that have associated raw data
- A description of any restrictions on data availability

Most data presented are included in the article and supplementary information. The source data underlying Figures 1-5 and Supplementary Figures 1-6 are provided in the Source Data file. All datasets generated are available from the corresponding author on request.

Field-specific reporting

Please select the one below that is the best fit for your research. If you are not sure, read the appropriate sections before making your selection.

☒ Life sciences ☐ Behavioural & social sciences ☐ Ecological, evolutionary & environmental sciences

For a reference copy of the document with all sections, see nature.com/documents/nr-reporting-summary-flat.pdf

Life sciences study design

All studies must disclose on these points even when the disclosure is negative.

Sample size	Sample sizes for all mouse data were chosen in advised and in agreement with the Institute of Epidemiology and Medical Biometry, Ulm University, and in agreement with EU regulations (Principles of Replacement, Reduction and Refinement (3R's), Directive 2010/63/EU) and as approved by the German "Regierungspräsidium". Sample size for human iPSC-derived DA neuron data was limited by the respective samples that we could obtain, but of sufficient size.
Data exclusions	No animals were excluded from the study. Criteria for exclusion/termination were pre-established together with the respective animal experiment permit. Outliers were tested via the ROUT function of GraphPad Prism 7.
Replication	All biological replications were successful. At least two independent researchers performed respective experiments with similar results. For MPTP experiments, at least two independent cohorts each were analyzed, showing similar results.
Randomization	No particular procedure was applied for randomization / allocating WT and KO mice to the respective experimental groups.
Blinding	The experimentor was blinded only for the stereological analysis of data. Other experiments were only partly blinded, but in most cases, at least two independent researchers performed experiments.

Reporting for specific materials, systems and methods

We require information from authors about some types of materials, experimental systems and methods used in many studies. Here, indicate whether each material, system or method listed is relevant to your study. If you are not sure if a list item applies to your research, read the appropriate section before selecting a response.

Materials & experimental systems

n/a	Involved in the study	
☐	☒	Antibodies
☒	☐	Eukaryotic cell lines
☒	☐	Palaeontology
☐	☒	Animals and other organisms
☒	☐	Human research participants
☒	☐	Clinical data

Methods

n/a	Involved in the study	
☒	☐	ChIP-seq
☒	☐	Flow cytometry
☒	☐	MRI-based neuroimaging

Antibodies

Antibodies used	Anti-Tyrosine Hydroxylase Rabbit pAb (Cat# 657012) from Calbiochem. http://1degreebio.org/review/6699/? qid=1572432<br>Anti-Tyrosine Hydroxylase Mouse pAb (Cat# 657012) from Merck Millipore<br>Anti-Tyrosine Hydroxylase Rabbit pAb (Cat# MAB318) from Merck Millipore<br>Anti-Tyrosine Hydroxylase Rabbit pAb (Cat# Ab152) from Sigma<br>Anti-NCS-1 Rabbit pAb (Cat# 10506-2-AP) from Proteintech<br>Anti-Cav2.3 Rabbit pAb (Cat#27225-1-AP) from Proteintech<br>Anti-NCS-1 Rabbit pAb (Cat# ab129166) from Abcam<br>Anti-Cav2.3 Rabbit pAb (anti-Nast 197B common, self-designed) obtained from Toni Schneider<br>Anti-beta actin HRP conjugated Mouse pAB (Cat# as-15) from Abcam<br>Anti-Tuj1 Mouse pAb (Cat# 801202) from Biolegend
Validation	NCS-1 and Cav2.3 antibodies were validated on respective KO mouse brain section. TH, Tuj1 and beta actin are common, well-established commercial antibodies.

Animals and other organisms

Policy information about studies involving animals; ARRIVE guidelines recommended for reporting animal research

Laboratory animals	Juvenile, adult, and aged male mice of the following strains were used: C57BL/6J, NCS-1 WT, NCS-1 KO, Cav2.3 WT and Cav2.3 KO mice. Construct of the NCS-1 KO is described in Ng et al. 2016, and of the Cav2.3 KO in Pereverzev et al. 2002.
Wild animals	No wild animals were used.
Field-collected samples	The study did not involve samples collected from the field.
Ethics oversight	All animal procedures were approved by the German Regierungspräsidium Tübingen (Ref: 35/9185.81-3; TV-No. 921, 1043 and 1291, Reg. Nr. o.147) or the German Landesamt für Natur, Umwelt und Verbraucherschutz Nordrhein-Westfalen (Ref: 84-02.04.2016.A505) and carried out in accordance with the approved guidelines.

Note that full information on the approval of the study protocol must also be provided in the manuscript.
